# Synthesis, structure and aromaticity of carborane-fused carbo- and heterocycles†
†Electronic supplementary information (ESI) available: CCDC 1583053–1583058 for **2**, **4**, **8**, **10**, **17** and **20**. For ESI and crystallographic data in CIF or other electronic format see DOI: 10.1039/c7sc04722c


**DOI:** 10.1039/c7sc04722c

**Published:** 2018-01-16

**Authors:** Tek Long Chan, Zuowei Xie

**Affiliations:** a Department of Chemistry and State Key Laboratory of Synthetic Chemistry , The Chinese University of Hong Kong , Shatin, New Territories , Hong Kong , China . Email: zxie@cuhk.edu.hk

## Abstract

Conjugation between a 3-D icosahedral carborane and a fused 2-D π-ring system is ambiguous. To address this issue, we prepared several carborane-fused carbo- and heterocycles. Detailed studies on their molecular structures, NMR data, and NICS (nucleus-independent chemical shift) and ISE (isomerization stabilization energy) values as well as molecular orbital analyses clearly suggest the presence of (1) considerable aromatic character in the *exo* five-membered ring of carborane-fused carbo- and heterocycles and (2) considerable conjugation between a 3-D carborane and a fused 2-D π-ring system. These results will shed some light on the design of new carborane-based materials.

## Abstract 2

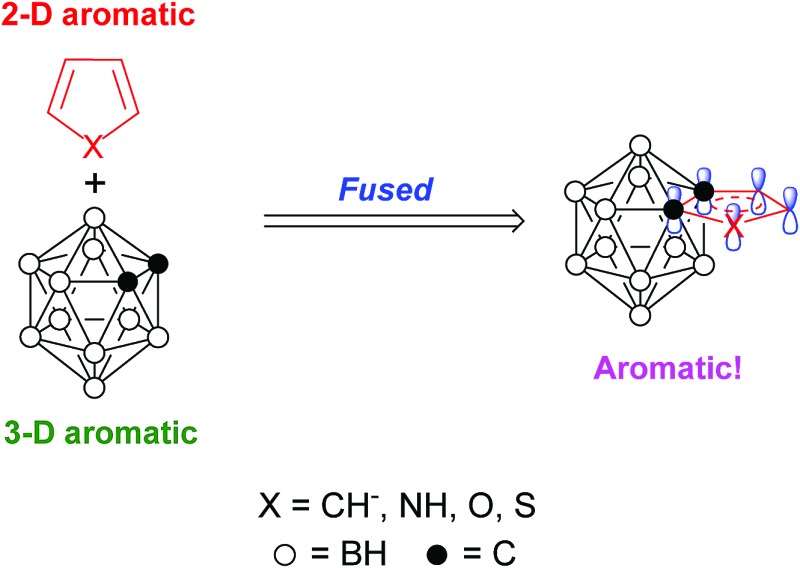
The results of molecular structures, NMR data, and NICS (nucleus-independent chemical shift) and ISE (isomerization stabilization energy) values as well as molecular orbital analyses clearly suggest the presence of considerable aromatic character in the *exo* five-membered ring of carborane-fused carbo- and heterocycles and considerable conjugation between a 3-D carborane and a fused 2-D π-ring system.

## Introduction

Icosahedral carboranes are carbon–boron molecular clusters, featuring a fully delocalized system of 26 skeletal electrons *via* 3c–2e bonding between tangential p orbitals and radial sp orbitals.^1^ Such clusters are aromatic molecules having 3-D aromaticity (σ-aromaticity),^2^ which are different from classical 2-D aromatic molecules such as benzenes (π-aromaticity).^3^ On the other hand, both classes of molecules share some common features such as thermal stability and ability to undergo electrophilic substitution reactions.^4^

**Fig. 1:**
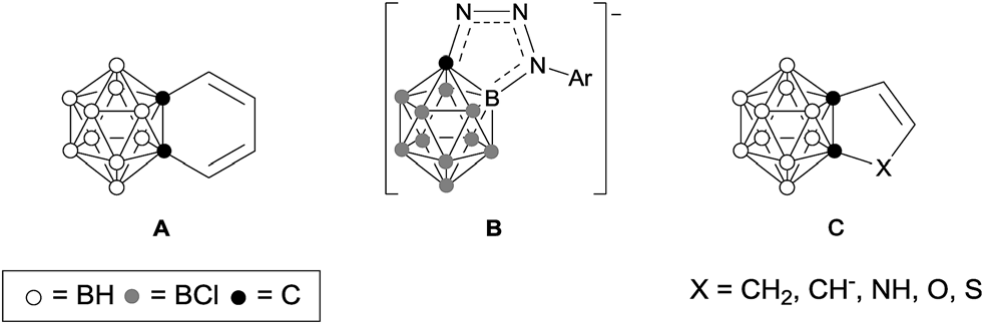
Carborane-fused carbo- and heterocycles.

Conjugation between a 3-D carborane and an *exo*-X atom *via* cage CX vertex (X = atom with π-donor ability) has been evidenced by experimental and theoretical results, and such a unique σ–π conjugation is enhanced as the π-donor ability of X atom increases.^5^ However, conjugation between a 3-D carborane and a fused 2-D π-ring system is ambiguous. No σ–π conjugation is observed between the C_2_B_10_ cage and the diene moiety in benzocarborane (**A** in Fig. 1), in which the C–C double bonds are localized with the NICS(0) (NICS: nucleus-independent chemical shift) values of –0.7 to –3.4 ppm for *exo*-C_6_H_4_ six-membered ring in benzocarborane derivatives.^6^ In contrast, a considerable σ–π conjugation is reported for CB_11_ cage-fused heterocyclic anion [1,2-N_3_R-1-CB_11_Cl_10_]^–^ (**B** in Fig. 1), leading to an aromatic *exo*-CBN_3_ five-membered ring with a NICS(0) value of –7.8 ppm.^7^

In view of wide applications of carboranes as a unique electronic sink and transmitter in optoelectronic materials,^8^ we initiated a research program to study the σ–π conjugation in *o*-carborane-fused five-membered carbo- and heterocycles, as well as the aromaticity of fused *exo* five-membered rings (**C** in Fig. 1). The results of this work would shed some light on the design of new carborane-fused π conjugated materials.

## Result and discussion

### Synthesis

**Scheme 1:**
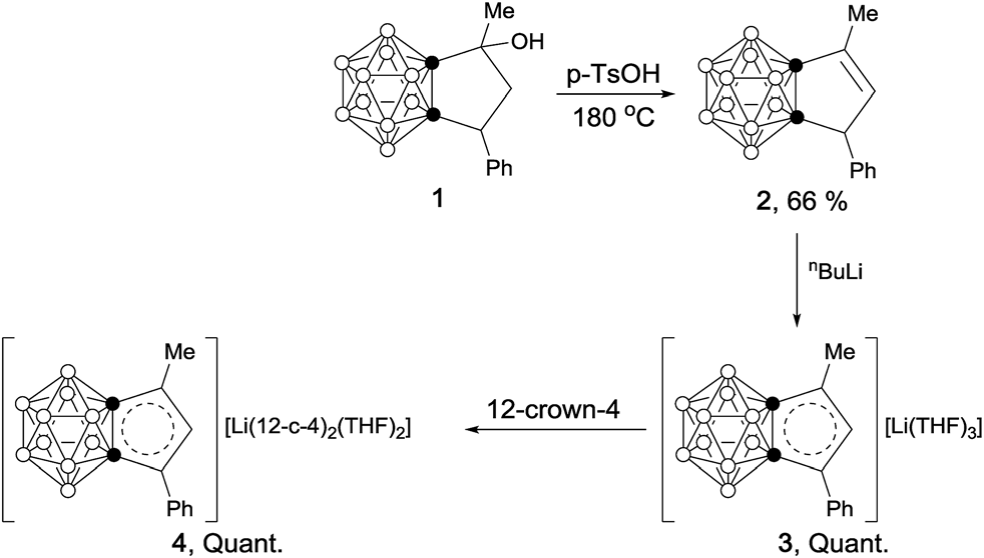
Synthesis of carborane-fused cyclopentadienyl anions.

Scheme 1 outlines the synthetic route to carborane-fused cyclopentadienyl anions. Compound **1** was synthesized according to the method given in the literature.^9^ Solvent-free dehydration of **1** in the presence of 1 equiv. of *p*-TsOH at 180 °C gave **2** as colorless crystals in 66% yield. Treatment of **2** with 1 equiv. of *n*-BuLi in THF afforded the corresponding lithium salt **3** as a yellow powder in quantitative yield. Recrystallization of **3** from a THF solution containing 12-crown-4 ether produced **4** as yellow crystals in quantitative yield.

**Scheme 2:**
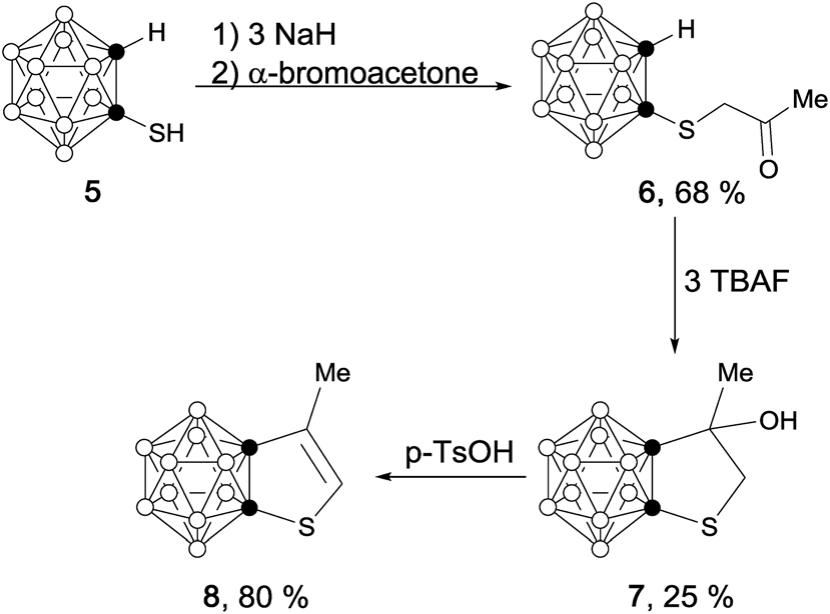
Synthesis of carborane-fused thiophene (**8**).

Treatment of 1-thio-*o*-carborane^10^ (**5**) with an excess amount of NaH followed by reaction with α-bromoacetone gave **6** as a white solid in 68% yield. Cyclization of **6** in the presence of tetrabutylammonium fluoride (TBAF) generated compound **7** as a white solid in 25% yield. The carborane-fused thiophene (**8**) was synthesized as colorless crystals in 80% yield *via* solvent-free dehydration in the presence of 1 equiv. of *p*-TsOH at 180 °C for 4 hours (Scheme 2).

**Scheme 3:**
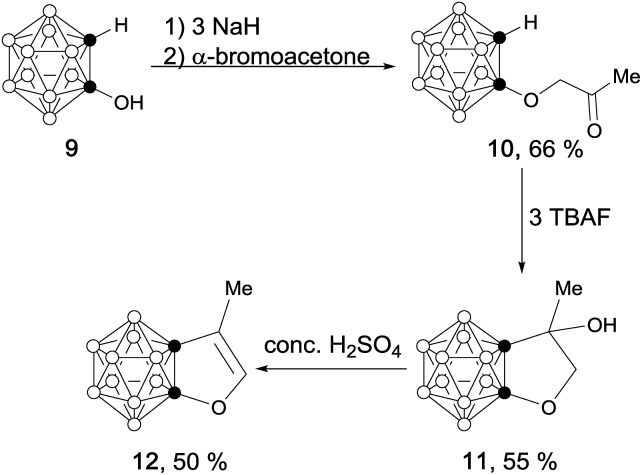
Synthesis of carborane-fused furan (**12**).

In a similar manner, carborane-fused furan (**12**) was prepared as a colorless liquid *via* concentrated H_2_SO_4_ mediated dehydration of **11** in 50% yield (Scheme 3).

**Scheme 4:**
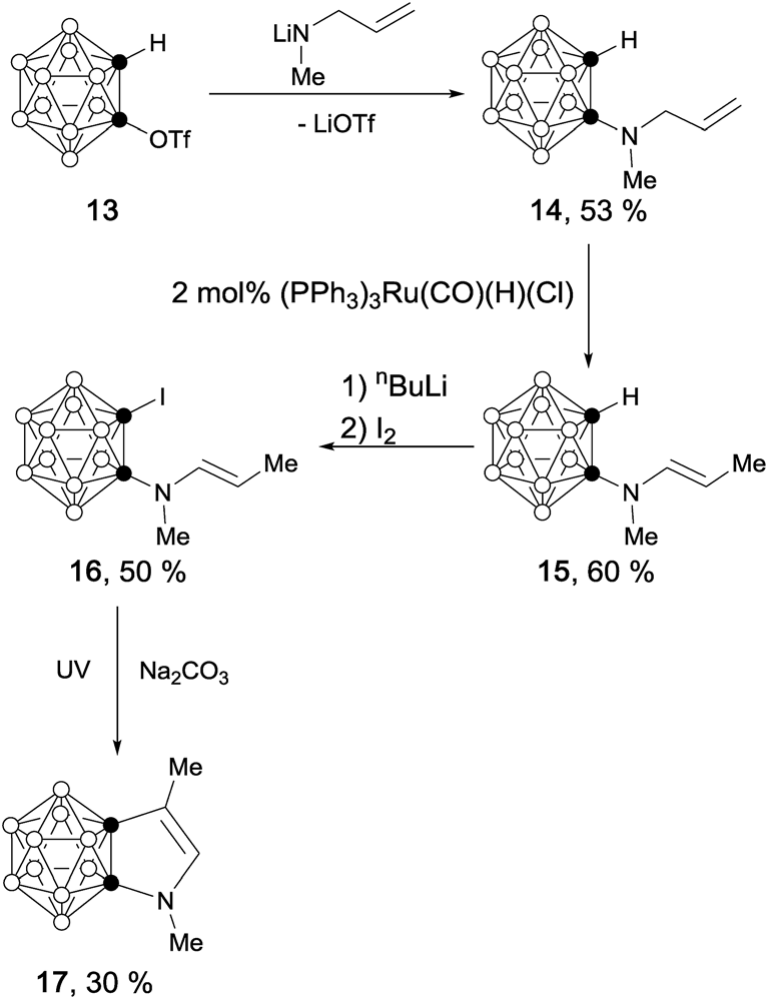
Synthesis of carborane-fused pyrrole (**17**).

The reaction of *o*-carboranyl triflate (**13**) with lithium *N*-allylmethylamide afforded the corresponding amine **14** as a colorless liquid in 53% yield.^11^ Ruthenium-catalyzed olefin isomerization^12^ and cage carbon iodination gave compound **16** as a light yellow solid in 50% yield. UV irradiation of **16** produced carborane-fused pyrrole (**17**) as colorless crystals in 30% yield (Scheme 4).^13^

**Scheme 5:**
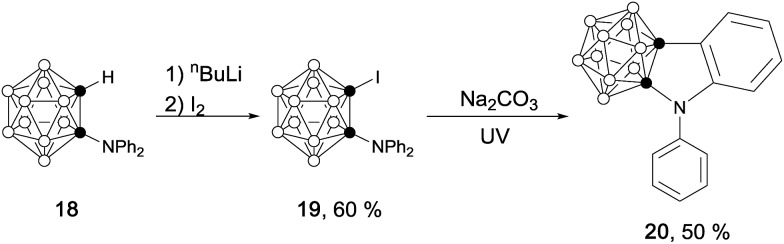
Synthesis of carborane-fused indole (**20**).

Similarly, iodination of **18**,^11^ followed by UV irradiation of **19** generated a carborane-fused indole (**20**) as pale yellow crystals in 50% yield (Scheme 5).^13^

### Structural characterization

NMR spectroscopy serves as a useful tool to determine whether a compound has aromatic character.^14^ The fused five-membered ring proton was observed at 6.53 ppm for **3** and 6.10 ppm for **4**, which was shifted downfield compared to that of 5.75 ppm in **2**, suggesting that the fused five-membered ring in **3** and **4** has some aromatic character. On the other hand, the CH proton in carborane-fused heterocycles was observed at 6.27 ppm in **8**, 6.58 ppm in **12** and 6.05 ppm in **17**, which was compared to that of 6.87 ppm in 3-methylthiophene,^15^ 7.16 ppm in 3-methylfuran^15^ and 6.35 ppm in 3-methyl-*N*-methylpyrrole,^16^ respectively. These measured proton chemical shifts were considerably downfield shifted in comparison to those observed in the corresponding dihydrothiophene,^17^ dihydrofuran,^18^ and dihydropyrrole,^19^ indicating that the fused five-membered rings in **8**, **12** and **17** have some aromatic character.

**Fig. 2:**
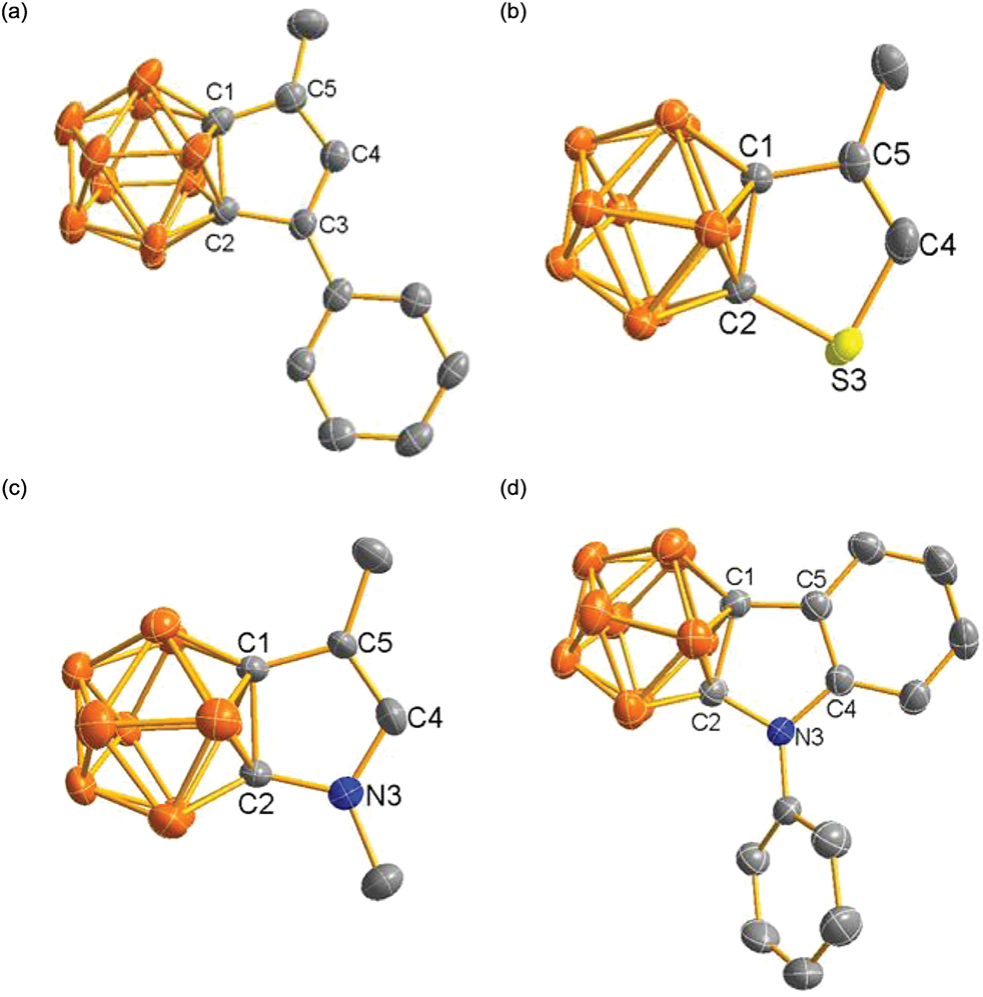
Molecular structures of (a) anion in **4**, (b) **8**, (c) **17** and (d) **20**.

Single-crystal X-ray analyses confirmed the molecular structures of **2**, **4**, **8**, **17** and **20**. Their representative structures are shown in Fig. 2. The results clearly indicate that the fused five-membered rings in **4**, **8**, **17** and **20** are co-planar with the sum of the internal pentagonal angles being 540°.

**Fig. 3:**
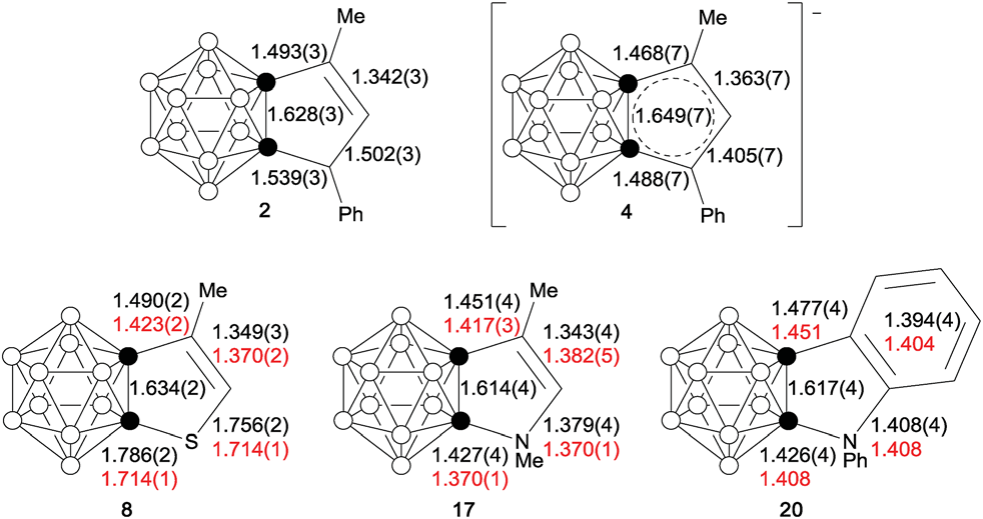
Selected bond distances (Å) of **2**, **4**, **8**, **17** and **20**. The distances in red are the experimental values (determined by the microwave spectroscopic method) of thiophene, pyrrole and indole.

As shown in Fig. 3, the bond distances of the fused five-membered ring in **4** are averaged in comparison with those observed in **2**, suggesting the presence of some degree of delocalization within the five-membered ring. Except for the cage C(1)–C(2) distance, the measured distances and angles of the fused five-membered heterocycles in **8**, **17** and **20** are comparable to those observed in thiophene,^20^ pyrrole21*a* and indole.21*b*

The UV-Vis spectra of **2**, **4**, **8**, **12**, **17** and **20** in THF were obtained, and they are shown in Fig. S7 and S8 in the ESI.† The UV-Vis spectrum of **4** (see Fig. S7 in the ESI†) displayed a new absorption band centered at 423 nm, which was assigned as π → π* absorption of the delocalized system of the *exo* five-membered ring.

The UV-Vis spectra of carborane-fused heterocycles (see Fig. S8 in the ESI†) showed absorptions between 287 and 290 nm attributable to n → σ* transition and between 306 to 330 nm corresponding to the π → π* transition of the delocalized *exo* five-membered ring system, which were red-shifted compared to those observed in heteroarenes (*λ*_max_ = 205–218 nm)^22^ and benzene-fused heterocycles (*λ*_max_ = 282–298 nm).^23^ These assignments were supported by time-dependent DFT (TD-DFT) calculations (see ESI†).

**Fig. 4:**
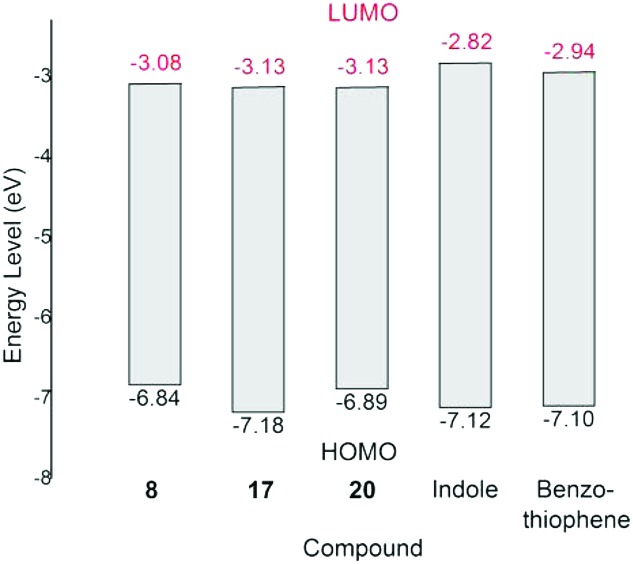
HOMO and LUMO energy levels of **8**, **17**, **20**, indole and benzothiophene.

The cyclic voltammograms of **8**, **17**, **20**, indole and benzothiophene obtained from solutions in THF are shown in Fig. S10 (ESI†). The absorption edge and reduction potentials of the aforementioned compounds are summarized in Table S1 in the ESI.† Based on these data, the energy levels of LUMO (the Lowest Unoccupied Molecular Orbital) and HOMO (the Highest Occupied Molecular Orbital) of these compounds were estimated and are shown in Fig. 4 (also see Table S1 in the ESI†). These results indicate that the electron-withdrawing nature of carboranyl moiety decreases the LUMO energy levels of **8**, **17** and **20** compared to those of benzo-fused heterocycles. The DFT-calculated (at the B3LYP/6-311++G(d,p) level of theory) HOMO energy levels are in general agreement with the corresponding experimental values, whereas the DFT-calculated LUMO energy levels are higher than the corresponding experimental values (Table S2 in ESI†). Such differences are often observed in aromatic π systems.^24^

### Computational studies

NICS values have been used extensively for the identification of aromatic properties of molecules.^25^ In this regard, NICS values of carborane-fused five-membered carbo- and heterocycles were calculated at the B3LYP/6-311++G(d,p) level of theory. For comparison, NICS values of cyclopentadiene, cyclopentadienide, thiophene, furan, pyrrole and their benzo derivatives were also calculated at the same level of theory. The results are compiled in Table 1. The data (–9.0 to –9.9 ppm) suggest that carborane-fused five-membered carbo- and heterocycles have considerable aromatic character. These results are consistent with those obtained from the aforementioned NMR data and structural parameters.

**Table 1:** Calculated NICS(0) values (ppm) of carborane-fused carbo- and heterocycles as well as the related typical five-membered aromatic ring at the B3LYP/6-311++G(d,p) level of theory

X	NICS(0)		
	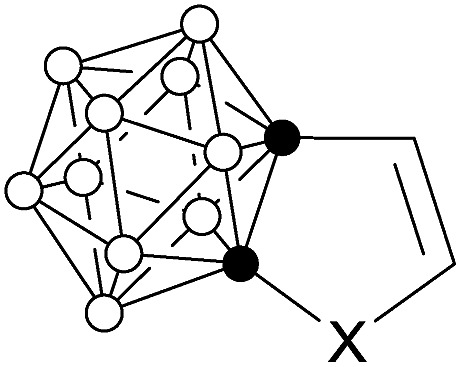	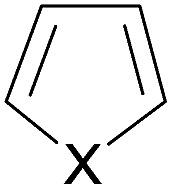	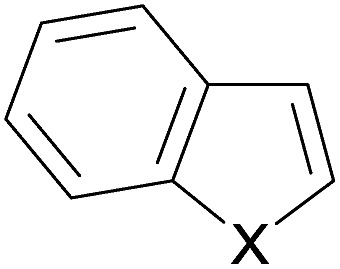
CH_2_	–5.8	–3.3	–0.7
CH^–^	–9.9	–12.6	–14.0
NH	–9.6	–13.7	–12.5
O	–9.3	–12.0	–10.9
S	–9.0	–14.2	–12.3

Another parameter used to estimate aromatic stabilization energy is the isomerization stabilization energy (ISE).^26^ The calculated ISE data for carborane-fused heterocycles and the related systems are summarized in Table 2. The results clearly show that the ISE values of carborane-fused thiophene, furan and pyrrole are about half (–6.4 to –8.8 kcal mol^–1^) of those calculated for benzo-thiophene, -furan and -pyrrole that are typical aromatic molecules.

**Table 2:** ISE values (kcal mol^–1^) of carborane-fused heterocycles and related five-membered heterocycles computed at the B3LYP/6-311+G(d,p) level of theory

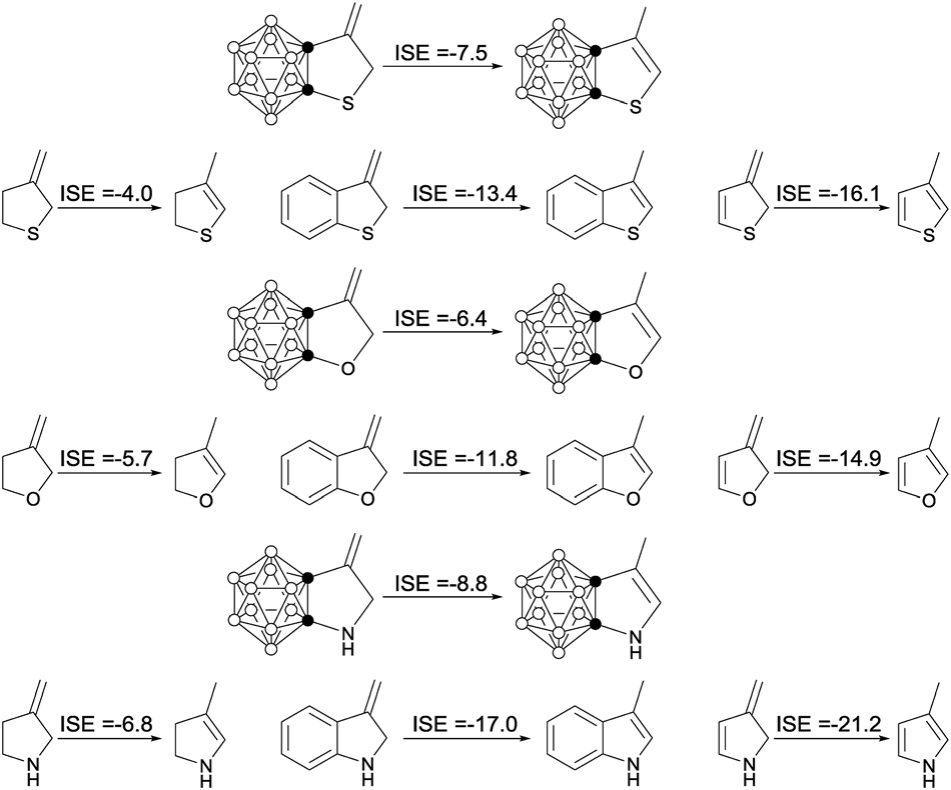

**Fig. 5:**
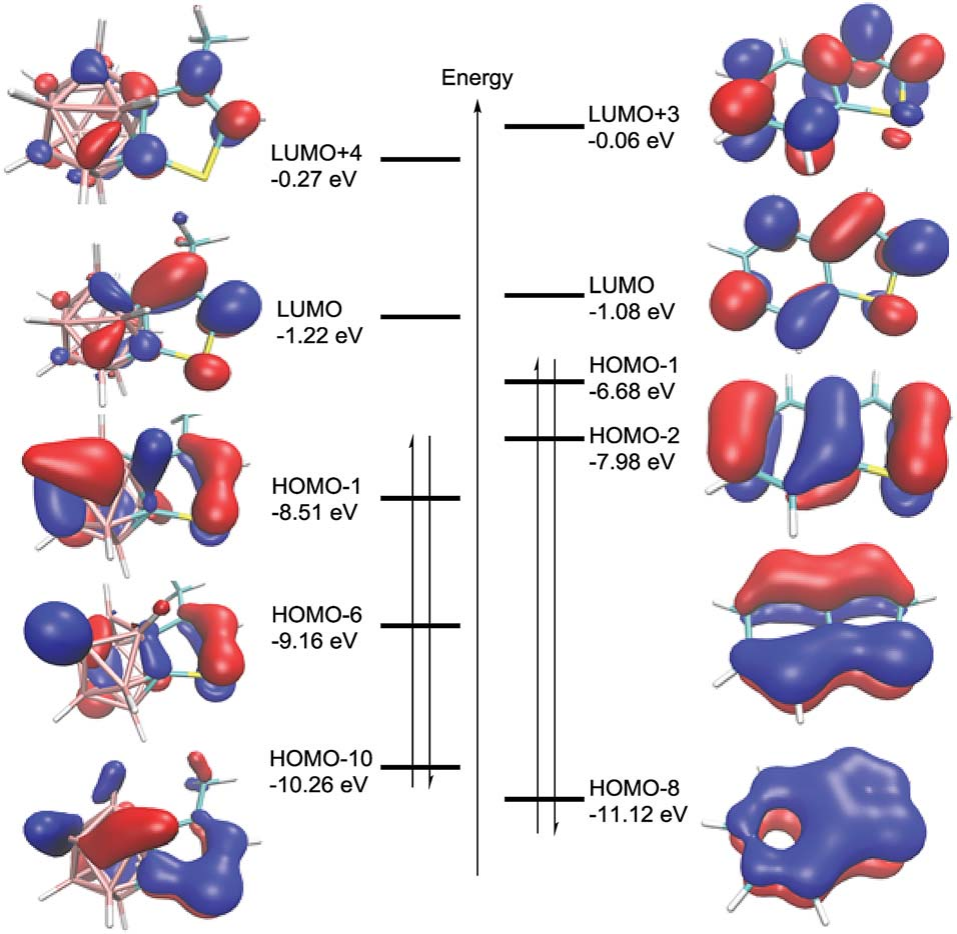
Plot of the LUMO+4, LUMO, HOMO–1, HOMO–6 and HOMO–10 orbitals in **8** (left) and the LUMO+3, LUMO, HOMO–1, HOMO–2 and HOMO–8 of benzothiophene (right) calculated at the B3LYP/6-311++G(d,p) level of theory, where red and blue indicate different phases of the wave functions.

To understand the electronic structures of carborane-fused heterocycles, DFT calculations at the B3LYP/6-311++G(d,p) level of theory were performed for both **8** and benzothiophene. The optimized bond distances and angles are in very good agreement with the experimental values of **8** (see Table S5 in the ESI†). Their selected molecular orbitals (MOs) are shown in Fig. 5, revealing the significant mixing between the carborane cage and the fused *exo* ring. It is noteworthy that the five MOs (HOMO–1, HOMO–6, HOMO–10, LUMO+4 and LUMO) in **8** resemble those found in benzothiophene, corresponding to π and π* MOs. These results again suggest that the carborane moiety can utilize its cage carbon p orbitals to participate in the *exo* π bonding interactions, resulting in considerable conjugation between a 3-D cage molecule and a 2-D π system.

## Conclusions

Several new carborane-fused cyclopentadienyl anion, furan, thiophene, pyrrole and indole have been prepared. NMR data, X-ray structural parameters, NICS and ISE values as well as molecular orbital analyses prove that the fused five-membered rings in the aforementioned carborane-fused cyclics have considerable aromatic character, and the conjugation between a 3-D cluster and a fused 2-D π system has been realized by the π donation of the *exo* heteroatom/carbanion. These results will shed some light on the design of new carborane-based π molecules for material applications.

## Conflicts of interest

There are no conflicts to declare.

## Supplementary Material

Supplementary information

Crystal structure data
