# Supplementary material for: Synthesis, structure and aromaticity of carborane-fused carbo- and heterocycles
Source: Chem Sci. 2018 Jan 16;9(8):2284–9. doi: 10.1039/c7sc04722c (PMC5897843; doi:10.1039/c7sc04722c)
Supplement: Supplementary file 1 [file SC-009-C7SC04722C-s001.pdf]

Electronic Supplementary Material (ESI) for Chemical Science.
This journal is © The Royal Society of Chemistry 2018

Electronic Supplementary Information

Synthesis, Structure and Aromaticity of Carborane-Fused Carbo- and Heterocycles

Tek Long Chan and Zuowei Xie*

Department of Chemistry and State Key Laboratory of Synthetic Chemistry,
The Chinese University of Hong Kong, Shatin, N. T., Hong Kong, China

Table of Contents

Experimental Section S2
Crystal Data and Summary of Data Collection and Refinement S14
Computational Details S17
References S29
^{1}H, ^{13}C and ^{11}B NMR spectra S31

General Procedures. All operations were carried out under a dry argon atmosphere using standard Schlenk or glovebox techniques. ^{1}H, ^{13}C and ^{11}B NMR spectra were recorded on a Bruker DPX 400 spectrometer at 400, 100 and 128 MHz, respectively. All chemical shifts were reported in δ units with references to the residual solvent resonances of the deuterated solvents for proton and carbon chemical shifts, and to external $BF_3 \cdot OEt_2$ (0.00 ppm) for boron chemical shifts. NMR multiplicities were abbreviated as follows: s = singlet, d = doublet, t = triplet, m = multiplet, br = broad signal. UV-Visible absorption spectra were recorded on a Varian Cary5G UV-Vis-NIR spectrophotometer (in the range of 200-800 nm) or Hitachi UH5300 spectrometer (in the range of 200-800 nm) using 1 cm quartz cells under argon atmosphere. Mass spectra were obtained on a Thermo Finnigan MAT 95 XL spectrometer. All organic solvents were freshly distilled from sodium benzophenone ketyl immediately prior to use. Compounds **1**,[1] **5**,[2] **9**,[3] **13**[4] and **18**[4] were prepared according to literature procedures. All other chemicals were purchased from Aldrich, J&K or Acros Chemical Co. and used as received unless otherwise specified.

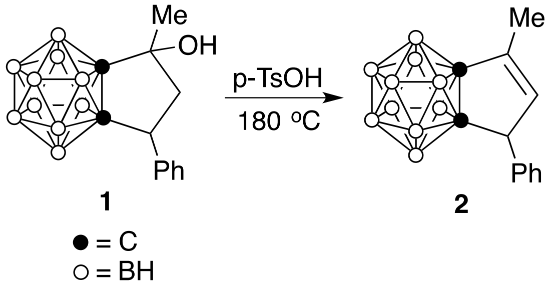

Preparation of 2. Compounds **1** (2.90 g, 10.0 mmol) and *p*-TsOH•H_2O (1.90 g, 10.0 mmol) were mixed in a sealed glass tube. The reaction mixture was heated at 180 °C for 6 h. The resulting black residue was dissolved in diethyl ether (20 mL). The solution was washed with water, saturated $NaHCO_3$ aqueous solution and brine, and then dried over anhydrous $MgSO_4$. Removal of the solvent afforded a pale yellow brown solid. Column chromatographic separation (silica gel, 230 – 400 mesh) using hexane as eluent afforded **2** as colorless crystals (1.80 g, 66%). ^{1}H NMR (400 MHz, $CDCl_3$): δ 1.99 (s, 3H; C*Me*), 4.60 (s, 1H; PhC*H*), 5.75 (s, 1H; C*H*=C), 7.15 (m, 2H; C_6H_5), 7.36 (m, 3H; C_6H_5). ^{13}C{^{1}H} NMR (100 MHz, $CDCl_3$): δ 14.4 (*C*H_3), 56.8 (Ph*C*H), 82.1 (cage *C*), 87.0 (cage *C*), 128.5, 128.6, 128.8 (aromatic *C*), 135.3, 135.8 (*C*H=*C*), 140.9 (aromatic *C*). ^{11}B{^{1}H} NMR (128 MHz, $CDCl_3$) δ -15.3 (1B), -11.7 (2B), -14.4 (1B), -10.5 (1B), -9.6 (2B), -7.4 (1B), -6.0 (2B). HRMS: *m/z* calcd for $C_{12}H_{20}{}^{10}B_2{}^{11}B_8{}^{+}$: 272.2563. Found: 272.2568.

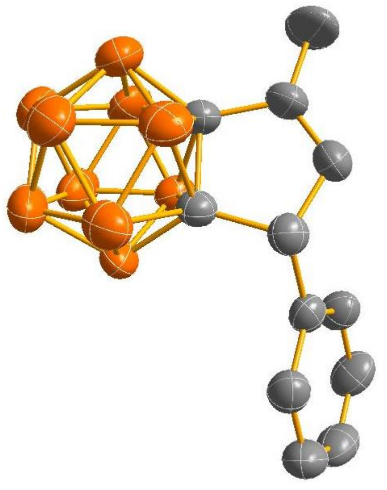

Figure S1. Molecular structure of **2**.

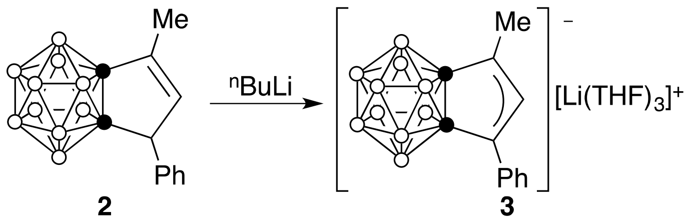

Preparation of 3. To a THF solution (20 mL) of **2** (131 mg, 0.48 mmol) was slowly added via syringe a hexane solution of n-BuLi (1.6 M, 0.3 mL, 0.48 mmol) at 0 ^{o}C under stirring. The reaction mixture was allowed to warm up to room temperature and stirred overnight. After removal of the solvent, **3** was isolated as a yellow powder (237 mg, 100 %). ^{1}H NMR (400 MHz, d_5-pyridine): δ 1.60 (m, 12H; Li(*THF*)$_3$), 2.42 (s, 3H; C*H*$_3$), 3.64 (m, 12H; Li(*THF*)$_3$), 6.53 (t, *J* = 7.1 Hz, 1H; C*H*=C), 7.17 (s, 1H; aromatic C*H*), 7.21 (s, 1H; aromatic C*H*), 7.26 (s, 1H; aromatic C*H*), 7.89 (d, *J* = 7.8 Hz, 2H; aromatic C*H*). ^{13}C{^{1}H} NMR (400 MHz, d_5-pyridine): δ 15.7 (*C*H$_3$), 25.9 (Li(*THF*)$_3$), 67.9 (Li(*THF*)$_3$), 85.8 (cage *C*), 88.1 (cage *C*), 102.2 (Ph*C*), 105.2 (Me*C*=CH), 114.4 (MeC=*C*H), 117.2, 129.0, 137.6, 141.2 (aromatic *C*).^{11}B{^{1}H} NMR (128 MHz, , d_5-pyridine): δ -18.4 (2B), -12.0 (7B), -3.0 (1B). HRMS: *m/z* calcd for $[C_{12}H_{19}{}^{10}B_2{}^{11}B_8]^-$: 271.2496. Found: 271.2490.

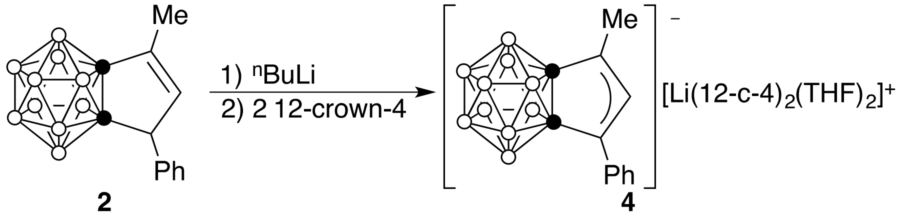

Preparation of 4. To a THF solution (20 mL) of **2** (131 mg, 0.48 mmol) was slowly added via syringe a hexane solution of n-BuLi (1.6 M, 0.3 mL, 0.48 mmol) at 0 ^{o}C under stirring. The reaction mixture was allowed to warm up to room temperature and stirred overnight. After removal

of solvent and washing with n-hexane, the yellow residue was mixed with 12-crown-4 ether (156 μL, 0.96 mmol) in THF under stirring for 24 h to give a brown solution. Removal of solvent afforded an orange-yellow solid. Recrystallization from a mixed THF/hexane solution (v/v=1/1, 5 mL) at room temperature produced **4** as yellow crystals (337 mg, 100 %). ^{1}H NMR (400 MHz, d_8-THF): δ 1.77 (s, 4H; Li(*THF*)), 2.05 (s, 3H; CH_3), 3.64 (br s, 20H; Li(*THF*) and 12-crown-4), 6.10 (t, *J* = 7.2 Hz, 1H; C*H*=C), 6.59 (s, 1H; aromatic C*H*), 6.75 (t, *J* = 7.6 Hz, 2H; aromatic C*H*), 7.17 (d, *J* = 8.0 Hz, 2H; aromatic C*H*). ^{13}C{^{1}H} NMR (400 MHz, d_8-THF): δ 15.2 (*C*H_3), 26.2 (Li(*THF*)), 68.0 (Li(*THF*)), 68.6 (12-crown-4), 85.6 (cage *C*), 87.8 (cage *C*), 101.4 (Ph*C*), 104.8 (Me*C*=CH), 113.3 (MeC=*C*H), 116.7, 128.5, 136.6, 141.2 (aromatic *C*). ^{11}B{^{1}H} NMR (128 MHz, d_8-THF): δ -18.9 (2B), -13.0 (6B), -4.0 (2B). HRMS: *m/z* calcd for $[C_{12}H_{19}{}^{10}B_2{}^{11}B_8]^-$: 271.2496. Found: 271.2490.

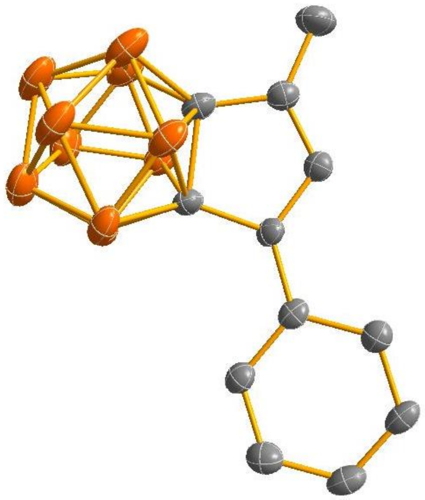

Figure S2. Molecular structure of the anion in **4**.

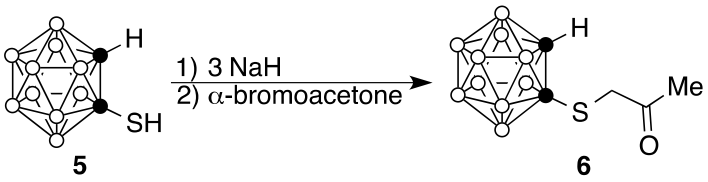

Preparation of 6. To a THF solution (20 mL) of **5** (1.76 g, 10.0 mmol) was added an excess amount of NaH (720 mg, 30.0 mmol), and the reaction mixture was stirred for 12 h at room temperature. After removal of the excess NaH by filtration, α-bromoacetone was added to the clear yellow solution with stirring for 12 h at room temperature. After removal of the solvent, the white residue was dissolved in diethyl ether (20 mL). The solution was washed with water and brine, and then dried over anhydrous $MgSO_4$. Removal of the solvent afforded a white solid. Column chromatographic separation (silica gel, 230 – 400 mesh) using hexane/diethyl ether (4:1) as eluent afforded **6** as a white solid (1.58 g, 68 %). ^{1}H NMR (400 MHz, $CDCl_3$): δ 2.29 (s, 3H; CH_3), 3.83

(s, 2H; SCH_2), 3.93 (br s, 1H; cage C*H*). $^{13}C\{^1H\}$ NMR (100 MHz, $CDCl_3$): δ 29.0 (CH_3), 47.8 (SCH_2), 67.7 (cage *C*), 73.7 (cage *C*), 200.6 (*C*O). $^{11}B\{^1H\}$ NMR (128 MHz, $CDCl_3$) δ -12.5 (3B), -9.2 (3B), -4.8 (2B), -1.3 (2B). HRMS: m/z calcd for $C_5H_{16}{}^{10}B_2{}^{11}B_8SO^+$: 232.1923. Found: 232.1929.

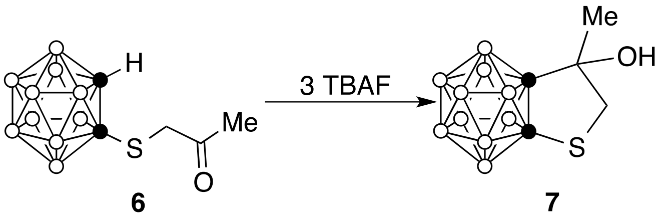

6 **7**

Preparation of 7. To a THF solution (20 mL) of **6** (1.16 g, 5.0 mmol) was slowly added via syringe a THF solution of TBAF (1.0 M, 15 mL, 15.0 mmol) at room temperature under stirring for 35 min. The reaction was quenched by a saturated aqueous NH_4Cl solution, and the mixture was extracted with ether, washed with saturated aqueous NaCl, dried over anhydrous $MgSO_4$, and then concentrated. Column chromatographic separation (silica gel, 230 – 400 mesh) using hexane/diethyl ether (8:1) as eluent afforded **7** as a white solid (290 mg, 25 %). ^1H NMR (400 MHz, $CDCl_3$): δ 1.70 (s, 3H; CH_3), 3.47 (d, *J* = 12.7 Hz, 1H; SCH_2), 3.87 (d, *J* = 12.7 Hz, 1H; SCH_2). $^{13}C\{^1H\}$ NMR (100 MHz, $CDCl_3$): δ 27.8 (CH_3), 47.8 (SCH_2), 91.3 (HO*C*Me), cage *C* were not observed. $^{11}B\{^1H\}$ NMR (128 MHz, $CDCl_3$) δ -14.4 (1B), -10.3 (2B), -7.4 (1B), -5.9 (1B). HRMS: m/z calcd for $C_5H_{16}{}^{10}B_2{}^{11}B_8SO^+$: 232.1923. Found: 232.1929.

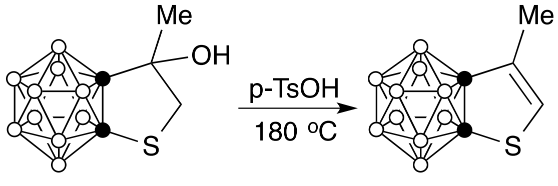

7 **8**

Preparation of 8. Compound **7** (232 mg, 1.0 mmol) and *p*-TsOH•H_2O (190 mg, 1.0 mmol) were mixed in a sealed glass tube. The reaction mixture was heated at 180 °C for 6 h. The resulting black residue was dissolved in diethyl ether (10 mL). The solution was washed with water, saturated $NaHCO_3$ aqueous solution and brine, and then dried over anhydrous $MgSO_4$. Removal of the solvent afforded a pale yellow brown solid. Column chromatographic separation (silica gel, 230 – 400 mesh) using hexane as eluent afforded **8** as a white solid (173 mg, 80 %). Recrystallization from n-hexane at -30 °C gave X-ray-quality crystals of **8**. ^1H NMR (400 MHz, $CDCl_3$): δ 2.09 (d, *J* = 1.40 Hz, 3H; CH_3), 6.27 (d, *J* = 1.36 Hz, 1H; C*H*=CMe). $^{13}C\{^1H\}$ NMR (100 MHz, $CDCl_3$): δ 16.0 (CH_3), 78.0 (cage *C*), 89.9 (cage *C*), 129.8 (H*C*=CMe), 132.3 (HC=*C*Me). $^{11}B\{^1H\}$ NMR (128

MHz, $CDCl_3$) δ -12.9 (2B), -10.5 (4B), -9.5 (1B), -8.7 (1B), -7.6 (2B). HRMS: m/z calcd for $C_5H_{14}{}^{10}B_2{}^{11}B_8S^+$: 214.1817. Found: 214.1818.

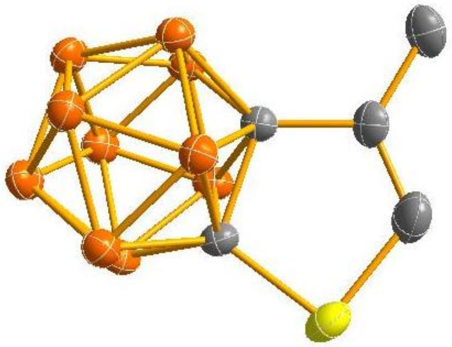

Figure S3. Molecular structure of **8**.

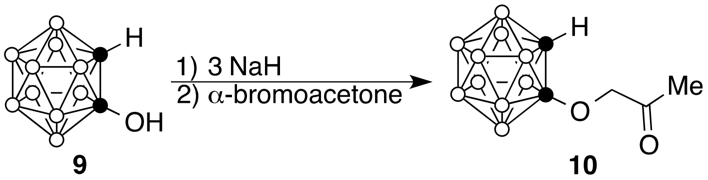

Preparation of 10. To a THF solution (20 mL) of **9** (1.60 g, 10.0 mmol) was added an excess amount of NaH (720 mg, 30.0 mmol), and the reaction mixture was stirred for 12 h at room temperature. After removal of the excess NaH by filtration, the α-bromoacetone was added to the clear yellow solution with stirring over 12 h at room temperature. After removal of the solvent, the white residue was dissolved in diethyl ether (20 mL). The solution was washed with water and brine, and then dried over anhydrous $MgSO_4$. Removal of the solvent afforded a white solid. Column chromatographic separation (silica gel, 230 – 400 mesh) using hexane/diethyl ether (4:1) as eluent afforded **10** as a white solid (1.43 g, 66 %). ^{1}H NMR (400 MHz, $CDCl_3$): δ 2.13 (s, 3H; CH_3), 4.24 (br s, 1H; cage C*H*), 4.39 (s, 2H; OCH_2). ^{13}C{^{1}H} NMR (100 MHz, $CDCl_3$): δ 26.0 (*C*H_3), 64.0 (O*C*H_2), 78.1 (cage *C*), 102.3 (cage *C*), 201.5 (*C*O). ^{11}B{^{1}H} NMR (128 MHz, $CDCl_3$) δ -10.3 (2B), -8.8 (4B), -7.8 (2B), -6.6 (1B), 0.2 (1B). HRMS: m/z calcd for $C_5H_{16}{}^{10}B_2{}^{11}B_8O_2^+$: 216.2151. Found: 216.2145.

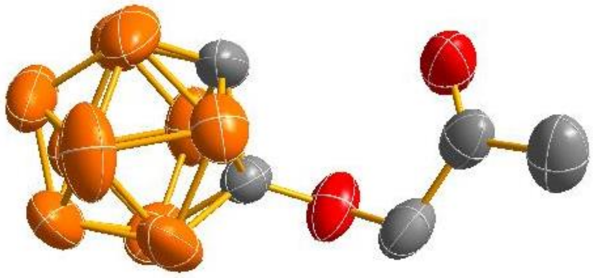

Figure S4. Molecular structure of **10**.

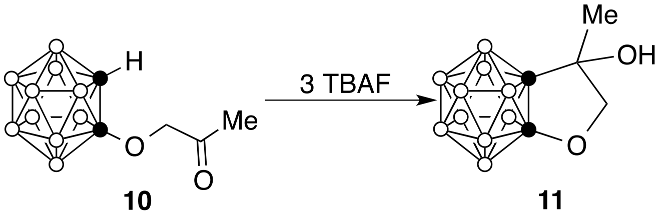

10 **11**

Preparation of 11. To a THF solution (20 mL) of **10** (1.08 g, 5.0 mmol) was slowly added via syringe a THF solution of TBAF (1.0 M, 15 mL, 15.0 mmol) at room temperature under stirring for 5 min. The reaction was quenched by a saturated aqueous NH_4Cl solution, and the mixture was extracted with ether, washed with saturated aqueous NaCl, dried over anhydrous $MgSO_4$, and then concentrated. Column chromatographic separation (silica gel, 230 – 400 mesh) using hexane/diethyl ether (8:1) as eluent afforded **11** as a white solid (594 mg, 55 %). ^{1}H NMR (400 MHz, $CDCl_3$): δ 1.71 (s, 3H; C*H*$_3$), 4.42 (d, *J* = 10.4 Hz, 1H; O*C*H$_2$), 4.47 (d, *J* = 10.4 Hz, 1H; OC*H*$_2$). ^{13}C{^{1}H} NMR (100 MHz, $CDCl_3$): δ 25.6 (*C*H$_3$), 78.1 (HO*C*Me), 93.4 (O*C*H$_2$), cage *C* were not observed. ^{11}B{^{1}H} NMR (128 MHz, $CDCl_3$) δ -15.8 (1B), -14.2 (2B), -12.4 (3B), -11.0 (1B), -10.2 (1B), -7.3 (2B). HRMS: m/z calcd for $C_5H_{16}{}^{10}B_2{}^{11}B_8O_2^+$: 216.2151. Found: 216.2145.

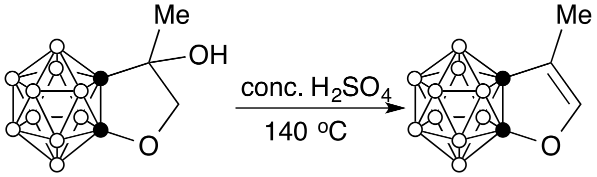

11 **12**

Preparation of 12. Compound **11** (432 mg, 2.0 mmol) and conc. H_2SO_4 (1.1 mL, 20 mmol) were mixed in a sealed glass tube. The reaction mixture was heated at 140 °C for 4 h. The resulting black residue was dissolved in diethyl ether (10 mL). The solution was washed with water, saturated $NaHCO_3$ aqueous solution and brine, and then dried over anhydrous $MgSO_4$. Removal of the solvent afforded a pale yellow brown solid. Column chromatographic separation (silica gel, 230 – 400 mesh) using hexane as eluent afforded **12** as an oil (218 mg, 50 %). ^{1}H NMR (400 MHz, $CDCl_3$): δ 1.91 (d, *J* = 1.48 Hz, 3H; C*H*$_3$), 6.58 (d, *J* = 1.4 Hz, 1H; C*H*=CMe). ^{13}C{^{1}H} NMR (100 MHz, $CDCl_3$): δ 9.0 (*C*H$_3$), 82.8 (cage *C*), 102.8 (cage *C*), 120.9 (H*C*=CMe), 152.8 (HC=*C*Me). ^{11}B{^{1}H} NMR (128 MHz, $CDCl_3$) δ -16.2 (2B), -13.0 (5B), -11.8 (1B), -8.5 (2B). HRMS: m/z calcd for $C_5H_{14}{}^{10}B_2{}^{11}B_8O^+$: 198.2045. Found: 198.2043.

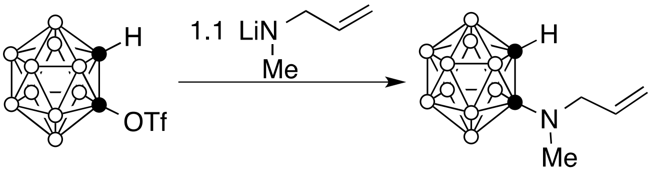

13 **14**

Preparation of 14. To a hexane solution (10 mL) of LiNMe($CH_2CH=CH_2$) (5.3 mmol), prepared in situ from the reaction of n-BuLi (1.6 M, 3.3 mL, 5.3 mmol) with HNMe($CH_2CH=CH_2$) (509 μL, 5.3 mmol) at 0 ^oC, was added a hexane solution (10 mL) of **13** (1.40 g, 4.8 mmol). The reaction mixture was stirred at room temperature for 10 min and then quenched with wet hexane. After removal of the solvents, the residue was subjected to flash column chromatography on silica gel (230 – 400 mesh) using n-hexane/Et_3N (100:1) as eluent afforded **14** as a light yellow oil (542 mg, 55 %). ^1H NMR (400 MHz, $CDCl_3$): δ 2.67 (s, 3H; CH_3), 3.62 (d, J = 5.6 Hz, 2H; NCH_2), 3.80 (br s, 1H; cage C*H*), 5.17 (t, J_1 = 16.8 Hz, J_2 = 10 Hz, 2H; $CH_2CH=CH_2$), 5.70 (m, 1H; $CH_2CH=CH_2$). $^{13}C\{^1H\}$ NMR (100 MHz, $CDCl_3$): δ 42.2 (NCH_3), 60.4 (NCH_2), 71.4 (cage *C*), 104.8 (cage *C*), 118.0 ($CH_2CH=CH_2$), 133.6 ($CH_2CH=CH_2$). $^{11}B\{^1H\}$ NMR (128 MHz, $CDCl_3$) δ -14.8 (3B), -12.9 (3B), -10.5 (2B), -3.9 (2B). HRMS: m/z calcd for $C_6H_{19}B_{10}N^+$: 213.2518. Found: 213.2513.

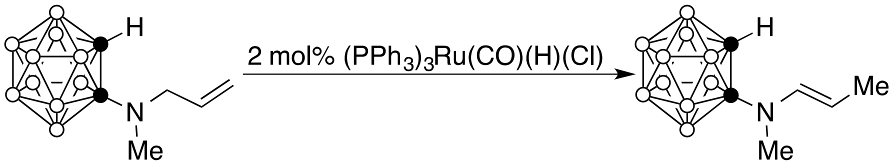

14 **15**

Preparation of 15. To a toluene solution of **14** (426 mg, 2.0 mmol) was added $(PPh_3)_3$(CO)(Cl)RuH (38.1 mg, 0.04 mmol). The resulting mixture was then sealed and allowed to stir at 85 ^oC for 12 h. After removal of solvent, the brown residue was dissolved in diethyl ether (10 mL). The solution was washed with water, and brine, and then dried over anhydrous $MgSO_4$. Removal of the solvent afforded a brown solid. Column chromatographic separation (silica gel, 230 – 400 mesh) using hexane/Et_3N (100:1) as eluent afforded **15** as a colorless liquid (256 mg, 60 %). ^1H NMR (400 MHz, $CDCl_3$): δ 1.66 (d, J = 9.6 Hz, 3H; CH_3), 2.91 (s, 3H; NCH_3), 3.79 (br s, 1H; cage C*H*), 4.65 (m, 1H; NCH=C*H*CH_3), 6.39 (d, J = 13.6 Hz, 1H; NC*H*=CHCH_3). $^{13}C\{^1H\}$ NMR (100 MHz, $CDCl_3$): δ 15.2 (CH_3), 40.0 (NCH_3), 68.0 (cage *C*), 99.6 (cage *C*), 103.5 (NCH=*C*HCH_3), 135.4 (N*C*H=CHCH_3). $^{11}B\{^1H\}$ NMR (128 MHz, $CDCl_3$) δ -13.8 (4B), -11.8 (4B), -9.1 (1B), -2.7 (1B). HRMS: m/z calcd for $C_6H_{19}{}^{10}B_2{}^{11}B_8N^+$: 213.2518. Found: 213.2513.

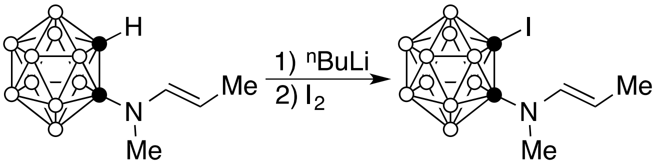

15 **16**

Preparation of 16. To an ether solution (10 mL) of **15** (341 mg, 1.6 mmol) was slowly added via syringe a hexane solution of n-BuLi (1.6 M, 1.0 mL, 1.6 mmol) at 0 ^oC under stirring.

The reaction mixture was allowed to warm up to room temperature and stirred for 2 h. I_2 (406 mg, 1.6 mmol) was added to the resulting mixture at room temperature and stirred overnight. The reaction mixture was quenched with saturated $Na_2S_2O_3$ aqueous solution. The solution was washed with water and brine, and then dried over anhydrous $MgSO_4$. Removal of the solvent afforded a yellow solid. Column chromatographic separation (silica gel, 230 – 400 mesh) using hexane/Et_3N (100:1) as eluent afforded **16** as a yellow solid (271 mg, 50%). ^{1}H NMR (400 MHz, $CDCl_3$): δ 1.75 (d, *J* = 6.6 Hz, 3H; C*H*$_3$), 3.26 (s, 3H; NC*H*$_3$), 4.99 (m, 1H; NCH=C*H*CH$_3$), 6.55 (d, *J* = 13.6 Hz, 1H; NC*H*=CHCH$_3$). ^{13}C{^{1}H} NMR (100 MHz, $CDCl_3$): δ 15.3 (*C*H$_3$), 34.1 (cage *C*), 40.7 (N*C*H$_3$), 106.7 (NCH=*C*HCH$_3$), 113.6 (cage *C*), 134.8 (N*C*H=CHCH$_3$). ^{11}B{^{1}H} NMR (128 MHz, $CDCl_3$) δ -11.6 (4B), -8.8 (3B), -7.6 (2B), -3.3 (1B). HRMS: m/z calcd for $C_6H_{18}{}^{10}B_2{}^{11}B_8NI^+$: 339.1485. Found: 339.1486.

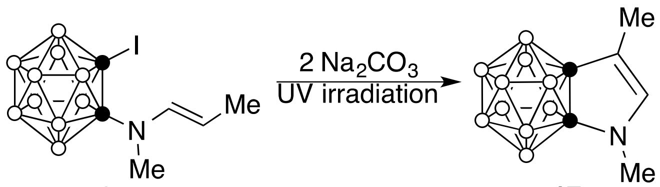

16 **17**

Preparation of 17. Compound **16** (170 mg, 0.5 mmol) and Na_2CO_3 (63.6 mg, 0.6 mmol) were mixed in CH_2Cl_2 (5 mL) in a sealed tube equipped with a magnetic stirring bar. The reaction mixture was irradiated with an UV lamp (365 nm, 36 W) at room temperature with stirring for 48 h. After removal of the solvent in vacuo, the residue was subjected to flash column chromatography on silica gel (230-400 mesh) using n-hexane as eluent to afford **17** as colorless crystals (50.6 mg, 30 %). ^{1}H NMR (400 MHz, $CDCl_3$): δ 1.87 (d, *J* = 1.2 Hz, 3H; C*H*$_3$), 2.96 (s, 3H; NC*H*$_3$), 6.05 (d, *J* = 1.2 Hz, 1H; CMe=C*H*). ^{13}C{^{1}H} NMR (100 MHz, $CDCl_3$): δ 11.0 (*C*H$_3$), 37.0 (N*Me*), 86.2 (cage *C*), 93.2 (cage *C*), 116.3 (NCH=*C*Me), 143.3 (N*C*H=CMe). ^{11}B{^{1}H} NMR (128 MHz, $CDCl_3$) δ -15.0 (1B), -13.2 (3B), -8.5 (1B). HRMS: m/z calcd for $C_6H_{17}{}^{10}B_2{}^{11}B_8N^+$: 211.2362. Found: 211.2362.

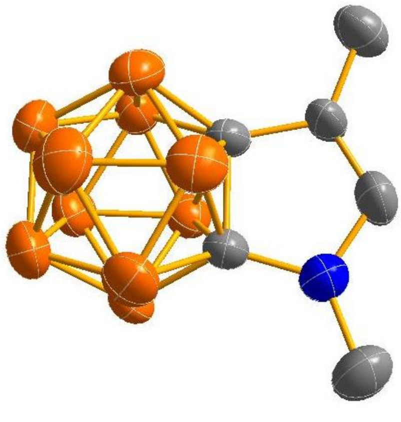

Figure S5. Molecular structure of **17**.

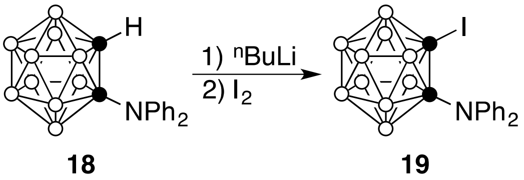

Preparation of 19. To an ether (10 mL) solution of **18** (498 mg, 1.6 mmol) was slowly added via syringe a hexane solution of n-BuLi (1.6 M, 1.0 mL, 1.6 mmol) at 0 °C under stirring. The reaction mixture was allowed to warm up to room temperature and stirred for 2 h. I_2 (406 mg, 1.6 mmol) was added to the resulting mixture at room temperature and stirred overnight. The reaction mixture was quenched with saturated $Na_2S_2O_3$ aqueous solution. The solution was washed with water and brine, and then dried over anhydrous $MgSO_4$. Removal of the solvent afforded a yellow solid. Column chromatographic separation (silica gel, 230 – 400 mesh) using hexane/Et_3N (100:1) as eluent afforded **19** as a yellow solid (419 mg, 60%). ^{1}H NMR (400 MHz, $CDCl_3$): δ 7.30 (m, 1H; C_6H_5), 7.38 (m, 1H; C_6H_5), 7.69 (m, 1H; C_6H_5). ^{13}C{^{1}H} NMR (100 MHz, $CDCl_3$): δ 128.2, 129.6, 130.4, 146.4 (aromatic *C*), cage *C* were not observed. ^{11}B{^{1}H} NMR (128 MHz, $CDCl_3$) δ -10.7 (4B), -8.8 (2B), -6.8 (2B), -2.8 (2B). HRMS: m/z calcd for $C_{14}H_{20}{}^{10}B_2{}^{11}B_8NI^+$: 437.1645. Found: 437.1648.

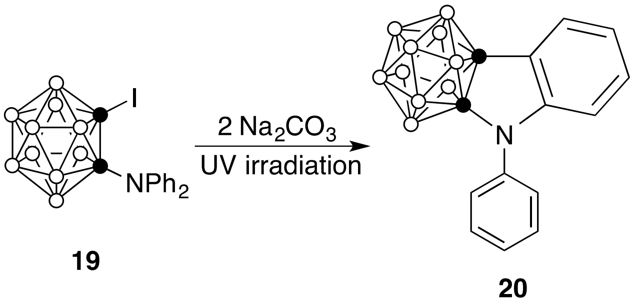

Preparation of 20. Compound **19** (219 mg, 0.5 mmol) and Na_2CO_3 (63.6 mg, 0.6 mmol) were mixed in CH_2Cl_2 (5 mL) in a sealed tube equipped with a magnetic stirring bar. Under an atmosphere of dry nitrogen, the reaction mixture was irradiated using an UV lamp (365 nm, 36 W) at room temperature with stirring for 48 h. After removal of the solvent in vacuo, the residue was subjected to flash column chromatography on silica gel (230-400 mesh) using n-hexane as eluent, affording **20** as colorless crystals (77.3 mg, 50 %). ^{1}H NMR (400 MHz, $CDCl_3$): δ 6.75 (d, *J* = 8.4 Hz, 1H; C_6H_4), 7.05 (t, *J* = 7.6 Hz, 1H; C_6H_4), 7.28 (merge with $CDCl_3$, 2H; C_6H_4), 7.32 (d, *J* = 8.0 Hz, 2H; C_6H_5), 7.48 (t, J = 7.2 Hz, 1H; C_6H_5), 7.51 (d, *J* = 6.2 Hz, 2H; C_6H_5). ^{13}C{^{1}H} NMR (100 MHz, $CDCl_3$): δ 78.9 (cage C), 92.5 (cage C), 112.9, 121.4, 121.6, 126.6, 126.9, 128.6, 129.6, 130.2, 139.0, 152.5 (aromatic *C*). ^{11}B{^{1}H} NMR (128 MHz, $CDCl_3$) δ -13.2 (3B), -11.8 (3B), -10.4 (2B), -8.6 (2B). HRMS: m/z calcd for $C_{14}H_{19}{}^{10}B_2{}^{11}B_8N^+$: 309.2522. Found: 309.2521.

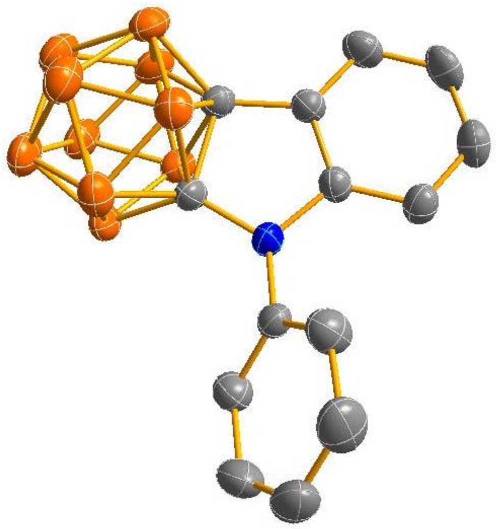

Figure S6. Molecular structure of **20**.

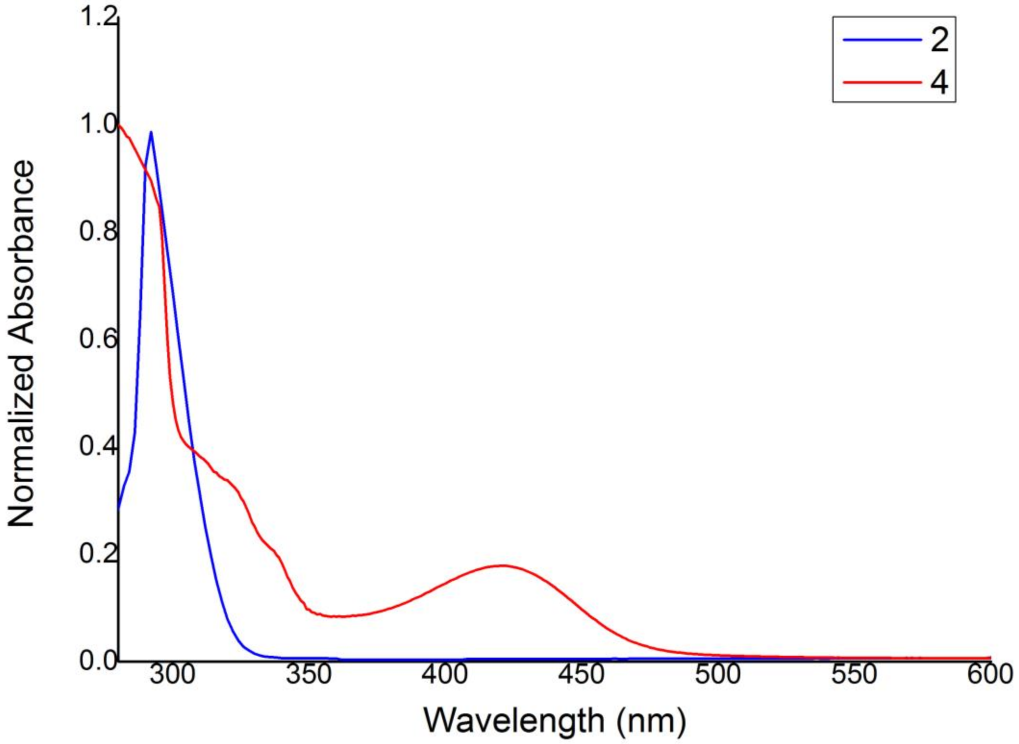

Figure S7. UV/Visible spectra of compounds **2** and **4** in THF.

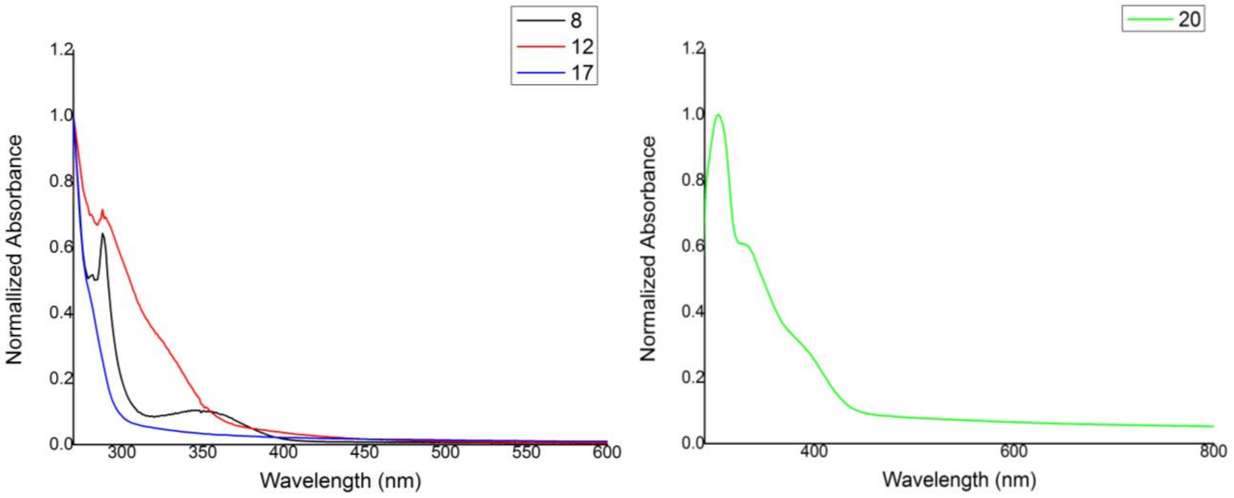

Figure S8. UV/Visible spectra of compounds **8**, **12**, **17** and **20** in THF.

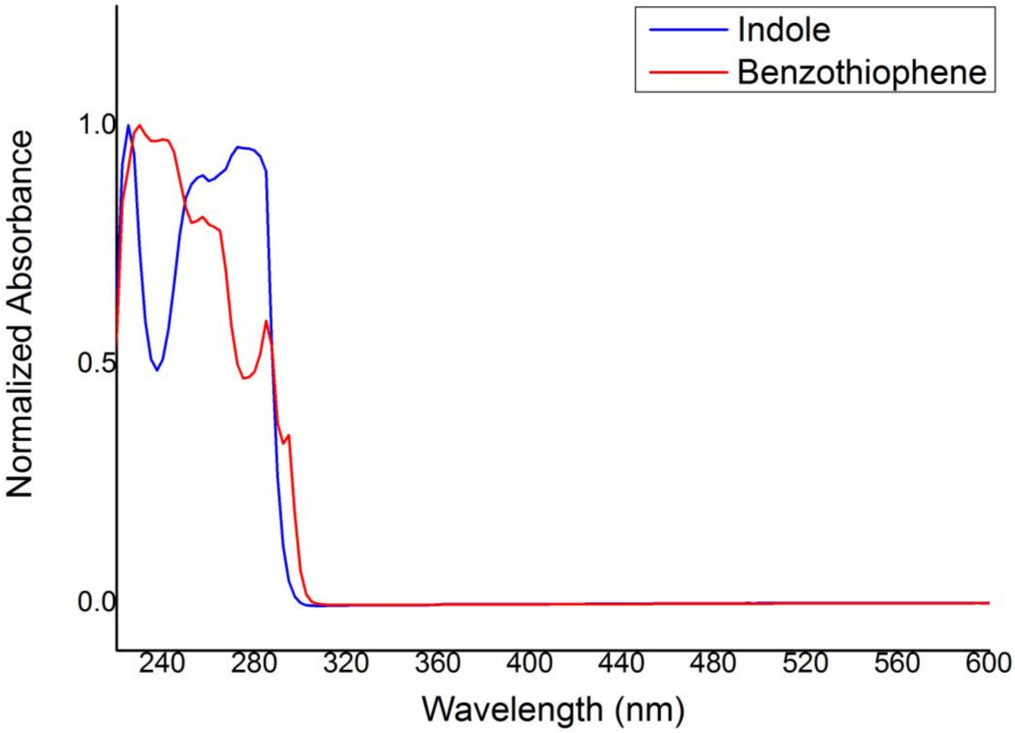

Figure S9 UV/Visible spectra of compounds thiophene and indole in THF.

Cyclic Voltammetry. Cyclic Voltammetry was performed under N_2 atmosphere with a CHI 600D potentiostation in a solution of anhydrous THF with 0.1 M tetrabutylammonium hexafluorophosphate as supporting electrolyte, at a scan rate of 50 mV s^{-1}. A glassy carbon was used as working electrode; a platinum wire was used as the auxiliary electrode, and an SCE was used as reference electrode. Under these conditions, $E_{1/2}$ = 0.56 V for the $FeCp_2^+/FeCp_2$ couple.

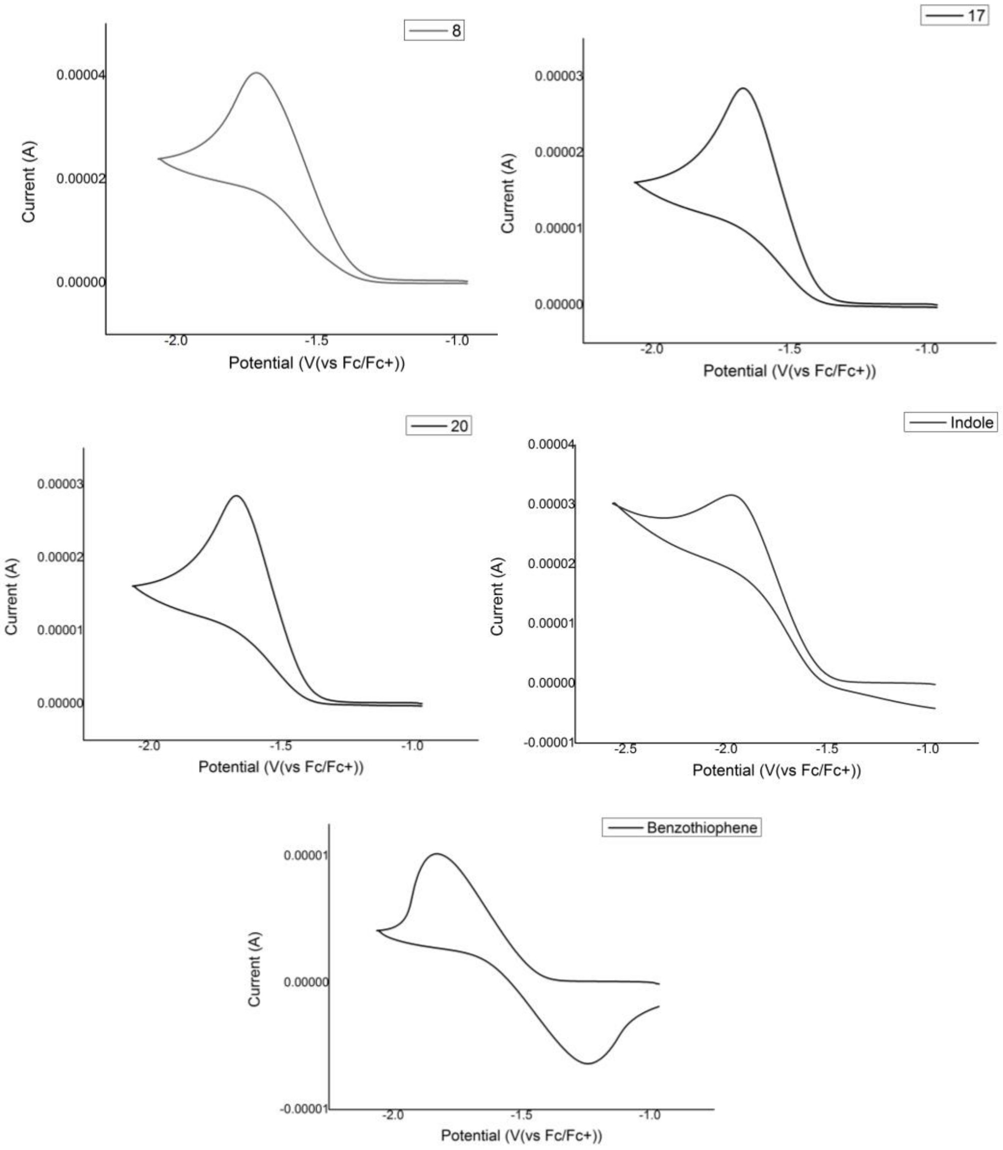

Figure S10. Cyclic voltammograms of **8**, **17**, **20**, indole and benzothiophene recorded in THF.

Table S1. Electrochemical and optical data of **8**, **17**, **20**, benzothiophene and indole.

Compound	Electrochemical		LUMO	HOMO	Optical	
	E_{red} (V)[a]	E_{ox} (V)[a]	(eV)[b]	(eV)[c]	λ_{max} (nm)	Egap (eV)
**8**	-1.72	--	-3.08	-6.84	330	3.76
**17**	-1.67	--	-3.13	-7.18	306	4.05
**20**	-1.67	--	-3.13	-6.89	330	3.76
Benzothiophene	-1.83	--	-2.94	-7.10	298	4.16
Indole	-1.98	--	-2.82	-7.12	288	4.30

[a] Potentials are reported versus ferrocenium/ferrocene as the peak wave for irreversible waves.

[b] The LUMO energy levels are estimated from LUMO = $-(E_{red} + 4.80)$ (eV).

[c] The HOMO energy levels of above compounds are estimated from their optical energy gap and LUMO energy levels

Table S2. The calculated data of **8**, **17**, **20**, benzothiophene and indole at the B3LYP/6-311++G(d,p) level of theory.

Compound	LUMO (eV)	HOMO (eV)	HOMO-LUMO *E*gap	Optical	
				λ_{max} (nm)	*E*gap (eV)
**8**	-1.22	-6.76	5.54	270	4.59
**17**	-0.73	-6.26	5.53	252	4.92
**20**	-1.33	-6.28	4.95	291	4.26
Benzothiophene	-1.08	-6.17	5.09	270	4.59
Indole	-0.64	-5.78	5.14	265	4.68

X-ray Structure Determination. Single crystals were immersed in Paraton-N oil. All data were collected at 296 K or 173 K on a Bruker D8 venture diffractometer or a Bruker Kappa ApexII Duo diffractometer using Mo-Kα radiation. An empirical absorption correction was applied using the SADABS program.[5] All structures were solved by direct methods and subsequent Fourier difference techniques and refined anisotropically for all non-hydrogen atoms by full-matrix least squares calculations on F^2 using the SHELXTL program package.[6] All hydrogen atoms were geometrically fixed using the riding model. Crystal data and details of data collection and refinement are given in Table S3. Details of the crystal structures were deposited in the Cambridge Crystallographic Data Centre with CCDC 1583053-1583058 for **2**, **4**, **8**, **10**, **17** and **20**.

Table S3. Crystal Data and Summary of Data Collection and Refinement for **2**, **4**, **8**, **10**, **17** and **20**.

Compound	**2**	**4**	**8**	**10**
Formula	$C_{12}H_{20}B_{10}$	$C_{36}H_{67}B_{10}LiO_{10}$	$C_5H_{14}B_{10}S$	$C_5H_{16}B_{10}O_2$
MW	272.38	774.93	214.32	216.28
Crystal size (mm^3)	0.50×0.50×0.40	0.40×0.30×0.20	0.50×0.40×0.30	0.50×0.40×0.30
Crystal system	Monoclinic	Triclinic	Monoclinic	Orthorhombic
Space Group	$P2_1$/n	P-1	$P2_1$/n	$P2_12_12_1$
a, Å	10.039(3)	11.967(2)	11.744(1)	6.955(1)
b, Å	14.627(4)	12.495(2)	8.504(1)	10.807(2)
c, Å	11.432(3)	14.935(2)	12.549(1)	16.754(2)
α, deg	90	87.27(1)	90	90
β, deg	96.92(1)	73.36(1)	108.23(1)	90
γ, deg	90	88.64(1)	90	90
V, $Å^3$	1666.4(9)	2158.0(4)	1190.3(2)	1259.3(3)
Z	4	2	4	4
D_{calcd} mg/m^3	1.086	1.193	1.196	1.141
Radiation (Å)	0.71073	0.71073	0.71073	0.71073
2θrange, deg	4.54 to 50.49	3.26 to 50.50	4.14 to 50.50	4.48 to 50.50
μ, mm^{-1}	0.052	0.079	0.223	0.063
F(000)	568	832	440	448
No. of obsd reflns	3016	7589	2126	2267
No. of params refnd	199	514	145	155
Goodness of fit	1.020	1.055	1.057	1.050
R1	0.0629	0.1105	0.0365	0.0830
wR2	0.1704	0.2813	0.1010	0.2047

Compound	**17**	**20**
Formula	$C_6H_{17}B_{10}N$	$C_{14}H_{19}B_{20}N$
MW	211.30	309.40
Crystal size (mm^3)	0.40×0.30×0.20	0.50×0.40×0.30
Crystal system	Monoclinic	Triclinic
Space Group	$P2_1/n$	P-1
a, Å	11.942(2)	8.998(1)
b, Å	8.526(1)	9.806(1)
c, Å	13.336(2)	11.248(1)
α, deg	90	87.61(1)
β, deg	108.73(1)	66.47(2)
γ, deg	90	73.90(2)
V, Å^3	1286.0(2)	871.5(1)
Z	4	2
D_{calcd} mg/m^3	1.091	1.179
Radiation (Å)	0.71073	0.71073
2θrange, deg	3.99 to 50.47	4.34 to 50.50
μ, mm^{-1}	0.051	0.059
F(000)	440	320
No. of obsd reflns	2315	3166
No. of params refnd	154	226
Goodness of fit	1.065	1.179
R1	0.0768	0.0701
wR2	0.2184	0.2086

Computational Details. The geometry optimizations of all compounds were performed using the Gaussian09 program, Revision D.01,[7] at the B3LYP[8] theoretical level using 6-311+G(d,p) basis set. Frequency calculations were made to determine the characteristics of all stationary points as energy minima. Orbital energies of compound **8** were calculated at the B3LYP/6-311++G(d,p) level of theory. Nucleus-independent chemical shifts (NICS) values were calculated at the B3LYP-GIAO/6-311++G(d,p) level. The ISE discussed were chosen to be the purely electronic energies.[9] Vertical electronic excitation energies were determined at the B3LYP/6-311++G(d,p) level by means of the time-dependent DFT (TDDFT) approach.[10] The graphics of the molecular orbitals were produced by using the visualizing software VMD.[11]

Table S4. The Cartesian coordinates for all the compounds calculated in this study.

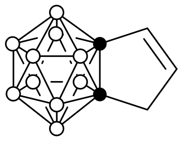

B	1.58659200	-0.01629900	1.44506300
B	0.66602500	-1.43926700	0.89007300
B	1.58706400	-0.01674200	-1.44476600
B	2.13981900	0.87060900	0.00018100
B	-0.15824800	0.01316100	-1.45201200
B	0.71416800	1.44228200	-0.89295800
B	0.71381700	1.44238800	0.89270500
B	0.66617100	-1.43946800	-0.88951700
B	2.11287500	-0.91608300	0.00035500
B	-0.15880400	0.01326000	1.45161500
C	-0.62742000	-0.79156100	-0.00002700
H	0.47829000	-2.45321200	-1.48220200
H	2.17894400	-0.02512900	-2.47702300
H	3.10237700	-1.57754400	0.00051800
H	0.47750600	-2.45286500	1.48280700
H	-0.96027700	0.02881400	2.32604600
H	0.56945200	2.46262700	1.48551100
H	0.56986200	2.46239100	-1.48600500
H	3.15124000	1.49803400	0.00015600
H	-0.95977200	0.02735400	-2.32643900
H	2.17799500	-0.02399200	2.47760400
C	-0.59594400	0.84238100	-0.00049800
C	-2.03695300	1.22407400	-0.00041800
H	-2.35087200	2.26262400	0.00015500
C	-2.83514300	0.14092400	0.00039700
H	-3.92049700	0.18744000	0.00083100
C	-2.10181500	-1.18222600	-0.00015000
H	-2.54577443	-1.89753314	-0.70295875
H	-2.09457317	-1.64901831	0.99213321

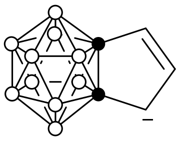

B	1.58112300	-0.00019400	-1.43190900
B	0.66211000	1.42765300	-0.90101800
B	1.58106500	0.00016200	1.43196500
B	2.10069100	-0.90765400	0.00014900
B	-0.15433600	0.00019200	1.47009400
B	0.66207000	-1.42765400	0.90103100
B	0.66209100	-1.42787200	-0.90064800
B	0.66206600	1.42787500	0.90067700
B	2.10069100	0.90760400	-0.00007600
B	-0.15428200	-0.00016300	-1.47009700
C	-0.69048800	0.82782600	-0.00012100
H	0.52983400	2.46019800	1.48546100
H	2.18081800	0.00033200	2.46647700
H	3.10533200	1.55589500	-0.00013200
H	0.52990300	2.45983400	-1.48605600
H	-0.93683800	-0.00035300	-2.36466900
H	0.52968000	-2.46015800	-1.48545500
H	0.52965200	-2.45980300	1.48607800
H	3.10536000	-1.55590100	0.00025200
H	-0.93693300	0.00023400	2.36463100
H	2.18089900	-0.00028800	-2.46640700

C	-0.69044500	-0.82773900	0.00009100
C	-2.09275700	-1.17759300	0.00011600
H	-2.45482900	-2.19778100	0.00022600
C	-2.09281200	1.17758800	-0.00021800
C	-2.84783900	-0.00002200	-0.00004100
H	-2.45494700	2.19775400	-0.00016100
H	-3.93832800	-0.00006000	-0.00005300

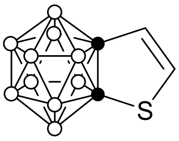

B	-1.78397100	-0.14242400	-1.44360800
B	-0.69422400	-1.44366400	-0.89643100
B	-1.78383300	-0.14280100	1.44374200
B	-2.43855100	0.66884000	0.00020400
B	-0.05465300	0.10031400	1.46340200
B	-1.09365100	1.40616200	0.89446700
B	-1.09373700	1.40639500	-0.89399500
B	-0.69413700	-1.44389700	0.89612000
B	-2.18950600	-1.10437700	-0.00003900
B	-0.05479300	0.10069600	-1.46336900
C	0.50521100	-0.64085700	-0.00010800
H	-0.37950300	-2.42750300	1.48379600
H	-2.36763500	-0.22446800	2.47715700
H	-3.08687700	-1.88589500	-0.00009700
H	-0.37964400	-2.42711500	-1.48439200
H	0.74016600	0.21056800	-2.33551200
H	-1.06857700	2.43837000	-1.48352900
H	-1.06843500	2.43798300	1.48426700
H	-3.51809000	1.16951900	0.00032200
H	0.74039100	0.20995800	2.33549700
H	-2.36787100	-0.22382100	-2.47699000
C	0.28985800	0.97628800	0.00011400
C	1.63107100	1.59877200	0.00013000
H	1.74930800	2.67737200	0.00026400
C	2.65406600	0.71874100	-0.00003400
H	3.70616100	0.98645600	-0.00005100
S	2.26404100	-0.99283200	-0.00023800

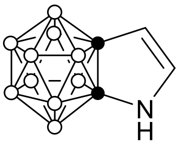

B	1.58342000	-0.01416100	-1.44152000
B	0.70344200	1.44173700	-0.89734400
B	1.58337600	-0.01381200	1.44156700
B	2.08697900	-0.92758500	0.00014100
B	-0.15185700	0.02520300	1.47500600
B	0.64369300	-1.43363500	0.90182700
B	0.64372100	-1.43385300	-0.90146400
B	0.70341600	1.44195400	0.89701300
B	2.12730600	0.87083600	-0.00007600
B	-0.15181400	0.02484600	-1.47501500
C	-0.62578800	0.84379700	-0.00011000
H	0.56587700	2.46825100	1.48109000
H	2.17641500	-0.02740500	2.47328400
H	3.14163400	1.49333100	-0.00013300
H	0.56591700	2.46789400	-1.48166900
H	-0.96501600	0.03271800	-2.33740900
H	0.45462600	-2.45540100	-1.48076800
H	0.45458300	-2.45504500	1.48137000
H	3.06959700	-1.59929100	0.00023900
H	-0.96508600	0.03328600	2.33737300
H	2.17648400	-0.02800300	-2.47322000
C	-0.64342200	-0.77862900	0.00008300
H	-2.35487800	-2.05413900	0.00028400
C	-2.06348700	1.18911500	-0.00017100
C	-2.79314800	0.04340500	-0.00004000
H	-2.44459200	2.20087100	-0.00029900
H	-3.87275400	-0.05342000	-0.00004000
N	-2.01516400	-1.10248900	0.00009300

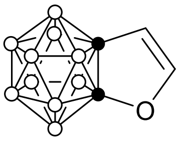

B	-1.56686500	-0.01652200	-1.44352400
B	-0.62672600	-1.43636500	-0.90041700
B	-1.56678300	-0.01691400	1.44360800
B	-2.11640400	0.86722200	0.00017800
B	0.16826400	0.02884500	1.47205200
B	-0.69793800	1.44961300	0.89777300
B	-0.69798900	1.44985700	-0.89734100
B	-0.62667400	-1.43660800	0.90006300
B	-2.07187600	-0.92625700	-0.00006700
B	0.16818000	0.02924300	-1.47205100
C	0.64281100	-0.76155300	-0.00012100
H	-0.42491700	-2.45335400	1.47921300

H	-2.16062600	-0.03131600	2.47424000
H	-3.05306600	-1.59923000	-0.00012900
H	-0.42500000	-2.45295400	-1.47985300
H	0.98689400	0.04192500	-2.32837300
H	-0.56470400	2.47426100	-1.48473400
H	-0.56462000	2.47385900	1.48543600
H	-3.13334700	1.48462900	0.00029100
H	0.98702700	0.04129400	2.32833000
H	-2.16076600	-0.03064500	-2.47412600
C	0.62115700	0.85550300	0.00009900
C	2.07170800	1.15511200	0.00009700
H	2.49635200	2.14997500	0.00021900
C	2.75212900	-0.00792600	-0.00008100
H	3.81708800	-0.19975100	-0.00013800
O	1.98086400	-1.16326000	-0.00021300

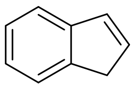

C	0.23230100	-0.68714500	0.00064500
C	0.21283900	0.72281500	0.00060100
C	-0.99932500	1.41773700	0.00004600
C	-2.18846600	0.68511700	-0.00006200
C	-2.16822700	-0.71581900	-0.00038200
C	-0.95490000	-1.41293900	0.00000400
C	2.43807200	0.13920100	-0.00081800
C	1.59684500	1.19821000	-0.00002800
H	-1.02097200	2.50603100	0.00000500
H	-3.14283700	1.20683200	-0.00063900
H	-3.10637800	-1.26603200	-0.00037600
H	-0.94720700	-2.50183500	-0.00035600
H	3.52259000	0.18829800	-0.00122400
H	1.88941167	2.24441820	0.00114126
C	1.66547700	-1.15398600	0.00015800
H	1.89955300	-1.76747800	0.88124700
H	1.89814200	-1.76938000	-0.87990200

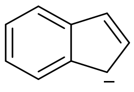

C	0.28705300	-0.73286600	-0.00001100
C	0.28705300	0.73287300	-0.00004800
C	-0.94772000	1.41686100	0.00002600
C	-2.14438900	0.70859400	0.00022100
C	-2.14438800	-0.70859700	0.00026300
C	-0.94771500	-1.41685900	0.00018400
C	1.63564700	-1.15682300	-0.00014600
C	2.44158500	-0.00000300	-0.00022400
C	1.63564800	1.15682100	-0.00027700
H	-0.96631100	2.51045400	-0.00002100
H	-3.09374300	1.24676200	0.00026100
H	-3.09374000	-1.24676900	0.00042100
H	-0.96630200	-2.51045100	0.00022100
H	1.98535700	-2.18736600	-0.00012700
H	3.53270400	0.00000600	-0.00033000
H	1.99028357	2.17167795	-0.16943548

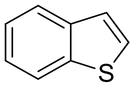

C	-0.06979100	-0.55093300	0.00004700
C	0.10908800	0.85552000	-0.00001400
C	1.41880600	1.37567100	-0.00007600
C	2.49854400	0.50558500	-0.00009300
C	2.30211400	-0.89004500	-0.00004700
C	1.02251800	-1.42969000	0.00001400
C	-2.21368200	0.69974300	0.00003300
C	-1.14535200	1.55014700	-0.00004600
H	1.57508900	2.45274600	-0.00011600
H	3.51082800	0.90245900	-0.00015800
H	3.16315200	-1.55402400	-0.00007500
H	0.87271100	-2.50704600	0.00006100
H	-3.26262793	0.96062681	-0.07637941
H	-1.24258400	2.63171700	-0.00007403
S	-1.75924500	-0.97407100	0.00008800

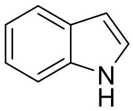

C	-0.24789400	-0.67063700	-0.00000100
C	-0.25231400	0.75245400	-0.00003600
C	0.98114000	1.43179400	0.00006300
C	2.15530100	0.69238300	0.00002600
C	2.13287600	-0.72006200	0.00002000
C	0.93470900	-1.42196000	-0.00004400
C	-2.38893000	0.02665000	-0.00003800
C	-1.62087800	1.16772700	-0.00002400
H	1.01134700	2.51976200	0.00003900
H	3.11375000	1.20607600	0.00009300
H	3.07208200	-1.26821000	-0.00000700

H	0.91891100	-2.51021900	-0.00003500
H	-3.46501000	-0.09120600	-0.00003500
H	-1.99677100	2.18282400	-0.00008900
N	-1.56305600	-1.07849600	0.00002600
H	-1.87448813	-2.03777056	0.07184046

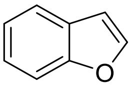

C	-0.25080400	-0.64816000	0.00009100
C	-0.25932200	0.75845100	-0.00012100
C	0.96894600	1.44126100	-0.00012400
C	2.13975300	0.69131300	-0.00012600
C	2.11132400	-0.71898400	0.00004600
C	0.90861500	-1.41981100	0.00007900
C	-2.35114200	-0.03001200	0.00004100
C	-1.64715100	1.13481500	-0.00000900
H	1.00375500	2.52840000	-0.00026300
H	3.10075400	1.19969000	-0.00012500
H	3.04840000	-1.27008600	0.00003600
H	0.86751300	-2.50517400	0.00020000
H	-3.40984500	-0.24814900	0.00012669
H	-2.06613699	2.13244199	-0.00019549
O	-1.53321900	-1.13629500	0.00010800

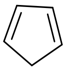

C	0.28574200	1.17775200	0.00005600
C	-0.99335400	0.73259700	0.00006100
C	-0.99362100	-0.73223500	-0.00005800
C	0.28531200	-1.17785600	-0.00006300
H	0.61262100	2.21179900	0.00011200
H	-1.88561816	1.35048249	0.00635509
H	-1.88613300	-1.34979600	-0.00007077
H	0.61181600	-2.21202100	-0.00012500
C	1.21545600	-0.00022100	0.00000000
H	1.87508100	-0.00029900	-0.87945600
H	1.87504200	-0.00038400	0.87948700

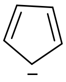

C	0.96911800	0.71121100	-0.00006000
C	0.97590800	-0.70186800	0.00031600
C	-0.37690100	1.14133200	0.00002000
C	-1.20208500	-0.00577600	-0.00016800
C	-0.36591700	-1.14489900	-0.00010700
H	-0.71917400	2.17643600	0.00004700
H	-2.29227501	-0.01101102	-0.00016522
H	-0.69822762	-2.18323972	0.00282094
H	1.84799200	1.35622800	-0.00011600
H	1.86093796	-1.33841111	0.00018804

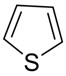

C	0.01529351	1.32368158	-0.00007790
C	1.28735551	0.79844758	-0.00005290
C	1.28735551	-0.62162042	0.00003810
C	0.01529351	-1.14685442	0.00006810
H	-0.27449899	2.36077955	0.10912813
H	2.18230451	1.41173458	-0.00009490
H	2.18230451	-1.23490642	0.00007090
H	-0.27910449	-2.18839542	0.00012910
S	-1.17818049	0.08841358	-0.00000290

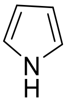

C	-0.30577947	-1.12569337	-0.00029816
C	1.01291253	-0.70912637	0.00037684
C	1.01295253	0.70934463	-0.00027416
C	-0.30571447	1.12598863	0.00015684
H	-0.74250915	-2.11461251	0.00079035
H	1.87814574	-1.35873682	-0.00051777
H	1.87822374	1.35890655	-0.00009035
H	-0.74238447	2.11493463	0.00067884
N	-1.09173847	0.00017063	-0.00004816
H	-2.10247395	0.00023931	0.01238938

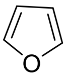

C	1.09613200	0.34829400	-0.00001500
C	0.71414700	-0.96315700	-0.00006800
C	-0.71414700	-0.96315800	0.00006600
C	-1.09613200	0.34829300	0.00008100
H	2.05183100	0.85073100	-0.00002400
H	1.37189249	-1.82117746	0.00663335
H	-1.37183847	-1.82118859	-0.00970895
H	-2.05188044	0.85054711	-0.00936673
O	-0.00008045	1.16489073	-0.01456058

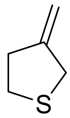

H	0.32183600	1.31725100	-1.50661900
C	0.47701500	1.23528400	-0.42389400
C	1.12394200	-0.08997500	-0.10695200
C	0.13036300	-1.21371700	-0.28022600
C	-0.88459000	1.22849200	0.28089800
H	1.09499800	2.07879900	-0.10690000
H	-1.59840700	1.91825000	-0.16947700
H	-0.78020400	1.46489100	1.34107900
C	2.37317600	-0.25566600	0.32357500
H	2.76652800	-1.23621700	0.56886100
H	3.04769300	0.58604000	0.43751700
H	0.33810200	-2.05226100	0.38398800
H	0.11817300	-1.57472100	-1.31188200
S	-1.53926000	-0.49553400	0.10019000

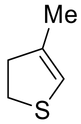

H	-0.48826300	1.55667900	1.24760900
C	-0.39210300	1.26889200	0.19062300
C	-1.01933300	-0.09782800	-0.00940100
C	-0.12583700	-1.09050900	-0.04794400
C	1.08589600	1.16034300	-0.21540800
H	-0.89700500	2.04424000	-0.39498700
H	1.74267700	1.81085400	0.36052100
S	1.57848300	-0.60369500	0.06975100
H	-0.35102000	-2.14703400	-0.11716800
H	1.21702800	1.37181500	-1.27848300
C	-2.50912500	-0.25814000	-0.04667600
H	-2.79913700	-1.30730700	-0.13185000
H	-2.96933800	0.14782700	0.86268500
H	-2.94766000	0.28549500	-0.89151000

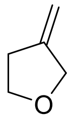

H	0.11041500	-1.55578700	-1.19638500
C	0.08048400	-1.21661200	-0.15537700
C	-0.85300900	-0.02946100	-0.02169300
C	0.03277900	1.20469100	0.03855100
C	1.43267700	-0.60640700	0.23513300
H	-0.21449700	-2.06116600	0.47062200
H	2.29304800	-1.07166200	-0.24630700
H	1.57672800	-0.63714000	1.32526800
C	-2.18042000	-0.04816600	0.05192300
H	-2.75853700	0.86570000	0.14338000
H	-2.73950800	-0.97692000	0.02137700
H	-0.02476100	1.67467800	1.03176100
H	-0.21609700	1.95548700	-0.71440200
O	1.36226800	0.74781800	-0.21581700

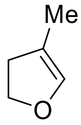

H	-0.04257000	-1.67945800	1.06361000
C	-0.15508800	-1.18678800	0.08880100
C	0.73850300	0.03743900	-0.00885300
C	-0.04741500	1.11411400	0.00647500
C	-1.55556800	-0.56769800	-0.10227900
H	0.06486600	-1.93673300	-0.67752000
H	-2.29817700	-0.90503300	0.62064700
H	0.21832900	2.16226900	-0.00230300
H	-1.93743000	-0.73834500	-1.11344800
C	2.23099300	-0.01827500	-0.03381000
H	2.66650300	0.98320400	-0.06857600
H	2.62611700	-0.52508100	0.85523900
H	2.59922200	-0.57395400	-0.90426300
O	-1.39567600	0.86754700	0.06557600

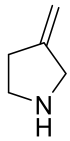

H	0.05796700	-1.56439200	-1.18795500
C	0.04367100	-1.22365000	-0.14731500
C	-0.86428100	-0.01302100	-0.01772700
C	0.02670400	1.21717900	0.05769900
C	1.42351900	-0.66924100	0.23354500
H	-0.27453700	-2.05954400	0.47881200
H	2.25048100	-1.21858100	-0.22197000
H	1.54984100	-0.70533600	1.32948300
C	-2.19293800	-0.01811100	0.04215600
H	-2.76383200	0.90106000	0.12478600
H	-2.75927700	-0.94270100	0.00882300
H	-0.01897500	1.63515600	1.07826100

H	-0.28226900	2.00205400	-0.63794100
N	1.35897000	0.70057800	-0.29091800
H	2.10776300	1.28930800	0.05398600

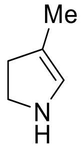

H	0.05796700	-1.56439200	-1.18795500
C	0.04367100	-1.22365000	-0.14731500
C	-0.86428100	-0.01302100	-0.01772700
C	0.02670400	1.21717900	0.05769900
C	1.42351900	-0.66924100	0.23354500
H	-0.27453700	-2.05954400	0.47881200
H	2.25048100	-1.21858100	-0.22197000
H	1.54984100	-0.70533600	1.32948300
C	-2.19293800	-0.01811100	0.04215600
H	-2.76383200	0.90106000	0.12478600
H	-2.75927700	-0.94270100	0.00882300
H	-0.01897500	1.63515600	1.07826100
H	-0.28226900	2.00205400	-0.63794100
N	1.35897000	0.70057800	-0.29091800
H	2.10776300	1.28930800	0.05398600

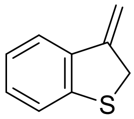

C	-2.48912500	0.98099000	0.00023100
C	-2.70028500	-0.40130800	-0.00004700
C	-1.62220600	-1.28215700	-0.00020400
C	-0.32766700	-0.76323700	-0.00009000
C	-0.09673000	0.62248000	0.00015500
C	-1.19462400	1.48932800	0.00033300
H	-3.33561300	1.65757800	0.00039500
H	-3.71093700	-0.79380800	-0.00011300
H	-1.78700700	-2.35325000	-0.00039300
H	-1.03697100	2.56197800	0.00058600
S	1.15012000	-1.74165500	-0.00039100
C	2.22594700	-0.23868600	0.00113400
H	2.86226900	-0.26812900	-0.88400400
C	1.32653100	0.99208800	0.00004900
C	1.82366400	2.23472700	-0.00085700
H	2.89185000	2.41783600	-0.00095500
H	1.18166100	3.10715600	-0.00153400

H	2.85980200	-0.26824100	0.88805600

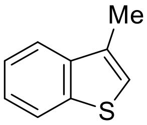

C	-2.35108600	-1.19005500	-0.00006600
C	-2.72131400	0.16495500	0.00010500
C	-1.75702300	1.16407200	0.00016200
C	-0.41138400	0.79024000	0.00004200
C	-0.01680500	-0.56814500	-0.00013700
C	-1.01433600	-1.55874900	-0.00018500
H	-3.12032900	-1.95380800	-0.00009600
H	-3.77102700	0.43546100	0.00018400
H	-2.04401000	2.20924000	0.00026700
H	-0.73762900	-2.60726900	-0.00028700
S	0.98497500	1.85253200	-0.00003300
C	1.42226300	-0.72871800	-0.00014500
C	2.05983600	0.47063000	-0.00012200
H	3.12668200	0.64215300	0.00008900
C	2.11011600	-2.06354200	0.00024100
H	1.83476300	-2.65156800	0.88202000
H	1.83509900	-2.65185900	-0.88144800
H	3.19525900	-1.94699800	0.00043100

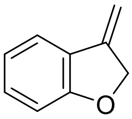

C	2.15648600	-1.17604800	0.00010200
C	2.56500900	0.16358000	-0.00017200
C	1.63811400	1.20587200	-0.00023300
C	0.29247000	0.86086000	-0.00004800
C	-0.13982500	-0.47031200	0.00018200
C	0.80139600	-1.49983700	0.00027300
H	2.90040900	-1.96381700	0.00019100
H	3.62365400	0.39764500	-0.00028500
H	1.94422800	2.24441800	-0.00038500
H	0.48434000	-2.53678000	0.00046500
O	-0.73012000	1.76109300	-0.00000600
C	-1.97659400	1.01813800	0.00027000
H	-2.53985200	1.31045500	-0.88937100
C	-1.60314300	-0.45774000	0.00004700
C	-2.46336000	-1.48100600	-0.00040400
H	-3.53585100	-1.32322600	-0.00068100
H	-2.12013000	-2.50860000	-0.00049300

H	-2.53915600	1.31010900	0.89049800

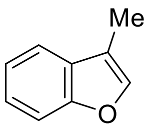

C	-2.04924500	-1.30515100	-0.00009100
C	-2.58525900	-0.00568800	-0.00008000
C	-1.76093400	1.11695200	-0.00003000
C	-0.39401300	0.88132700	0.00000700
C	0.17335800	-0.40345200	-0.00000300
C	-0.67518500	-1.51866800	-0.00005200
H	-2.72295700	-2.15437300	-0.00013100
H	-3.66110000	0.12584300	-0.00011100
H	-2.15982300	2.12368800	-0.00002000
H	-0.27292100	-2.52550200	-0.00006100
O	0.58926000	1.83069200	0.00005900
C	1.60876700	-0.20375300	0.00004500
C	1.78016300	1.14062100	0.00007500
H	2.66558400	1.75571900	0.00011200
C	2.66852000	-1.25984600	0.00005600
H	2.58653100	-1.90364500	0.88162000
H	2.58660200	-1.90359000	-0.88155400
H	3.66696300	-0.81772200	0.00011100

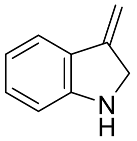

C	2.17152300	-1.17232800	-0.03734900
C	2.57937200	0.16584300	-0.00160200
C	1.65173400	1.20617800	0.04238000
C	0.29486800	0.88033300	0.04374500
C	-0.12756400	-0.46207500	-0.00681300
C	0.81333300	-1.48881400	-0.03940700
H	2.91293100	-1.96175600	-0.06972100
H	3.63800300	0.40143300	-0.00539000
H	1.97852900	2.23940000	0.08240300
H	0.49394400	-2.52457300	-0.07960600
C	-2.02937800	0.97627100	-0.12546100
H	-2.40903800	1.17953700	-1.13573300
C	-1.59516600	-0.48271500	-0.01070800
C	-2.42024100	-1.53125600	0.08326700
H	-3.49740400	-1.40923200	0.08469200
H	-2.04281700	-2.54396400	0.16163900
H	-2.81268200	1.23323400	0.59120000
N	-0.79675000	1.74126900	0.13963200
H	-0.71509900	2.64841900	-0.29521700

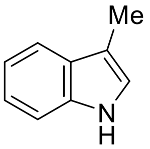

C	-2.04843600	-1.31667800	-0.00005300
C	-2.59907700	-0.02027500	-0.00013600
C	-1.78730100	1.10643700	-0.00011600
C	-0.40484500	0.90690900	-0.00002500
C	0.17327800	-0.39077900	0.00004800
C	-0.67440900	-1.51030100	0.00003900
H	-2.71251900	-2.17360000	-0.00006700
H	-3.67647000	0.10028700	-0.00019400
H	-2.21488800	2.10331100	-0.00014000
H	-0.26175700	-2.51341500	0.00008100
C	1.60610600	-0.22570200	0.00005500
C	1.83643600	1.12422400	-0.00007300
H	2.77383200	1.65947000	-0.00016000
C	2.63201700	-1.31693600	0.00007000
H	2.53517000	-1.96020000	0.88130900
H	2.53512300	-1.96026100	-0.88111800
H	3.64426500	-0.90591300	0.00002800
N	0.63385700	1.81349500	0.00013500
H	0.53762700	2.81445700	0.00046700

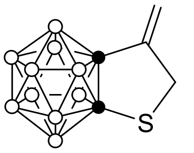

S	-1.74319300	-1.74574200	-0.00188000
C	-0.33519300	0.70098700	0.00018100
C	-0.13973100	-0.90972800	-0.00072200
C	-2.23732600	2.30197600	-0.00193600
C	-1.79035100	1.04973100	0.00019100
C	-2.71445700	-0.16365100	0.00371400
H	-3.35344400	-0.16243400	-0.87996300
B	0.22353600	-0.05019900	-1.47033800
H	-0.55843800	-0.13995700	-2.34360400
B	0.90938400	1.47599100	-0.89097500
H	0.62968200	2.45989500	-1.47784400
B	0.90897800	1.47487700	0.89296800
H	0.62890300	2.45804000	1.48089200

B	0.22285800	-0.05207300	1.47004700
H	-0.55955900	-0.14290800	2.34283200
B	1.23014400	-1.38904300	-0.89618600
H	1.18181100	-2.41083500	-1.48175600
B	1.96077000	0.14846900	-1.44910300
H	2.54551200	0.21577700	-2.47348100
B	2.39773500	1.09304400	0.00108800
H	3.31162200	1.84210300	0.00174800
B	1.96013400	0.14668400	1.44990100
H	2.54438000	0.21274800	2.47464300
B	1.22982500	-1.39011600	0.89483800
H	1.18119200	-2.41260900	1.47914700
B	2.59521700	-0.68769900	0.00003800
H	3.65296100	-1.21418800	-0.00006900
H	-1.55939900	3.14593100	-0.00392200
H	-3.29940200	2.51807500	-0.00177600
H	-3.34528400	-0.16332600	0.89327200

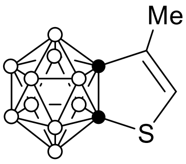

S	1.63880000	-1.85927000	0.00012200
C	0.39442600	0.63724300	0.00006200
C	0.06935800	-0.95951900	0.00000300
C	2.48773900	2.18274000	0.00012200
H	3.57660700	2.12076100	-0.00001800
H	2.17400000	2.75113700	0.88077300
H	2.17378900	2.75111700	-0.88045400
C	1.87908900	0.81300000	0.00009900
C	2.55569500	-0.34479200	-0.00031700
H	3.63372600	-0.43344200	-0.00057200
B	-0.23083900	-0.07485700	1.47395000
H	0.54143600	-0.22699700	2.34687900
B	-0.78606100	1.49946100	0.89510700
H	-0.44562400	2.46398100	1.48256800
B	-0.78598700	1.49954700	-0.89502000
H	-0.44549400	2.46410900	-1.48237200
B	-0.23069600	-0.07475500	-1.47394200
H	0.54164400	-0.22685100	-2.34682600
B	-1.33325600	-1.33416900	0.89688500
H	-1.36125000	-2.35699600	1.48294900
B	-1.94323900	0.25500700	1.44690400
H	-2.52021600	0.36687800	2.47194800
B	-2.30003700	1.23577200	-0.00001900
H	-3.15387600	2.05277900	-0.00002000
B	-1.94307900	0.25511500	-1.44695700
H	-2.51998300	0.36709500	-2.47203200
B	-1.33316900	-1.33412700	-0.89703100
H	-1.36112100	-2.35691600	-1.48316600
B	-2.64081400	-0.52543700	-0.00011800
H	-3.73640200	-0.96815200	-0.00021200

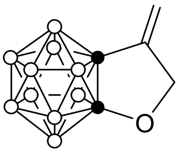

C	0.57386700	-0.53004000	0.00016400
C	0.16256600	1.03118700	-0.00035300
C	2.87431500	-1.54746700	-0.00016900
H	3.95181900	-1.42922900	-0.00031000
C	2.06491100	-0.49563900	0.00012500
C	2.49028400	0.97046200	0.00049700
H	3.06882600	1.22378300	0.89151500
B	-0.08469100	0.13029700	-1.47534600
H	0.69266000	0.34264100	-2.33268600
B	-0.53383700	-1.47915600	-0.89490700
H	-0.12028600	-2.41744900	-1.47742500
B	-0.53384400	-1.47854900	0.89591700
H	-0.12027800	-2.41642800	1.47908600
B	-0.08470700	0.13132600	1.47522800
H	0.69261900	0.34421000	2.33246200
B	-1.25493200	1.32245800	-0.89917200
H	-1.35111100	2.34329600	-1.47952000
B	-1.76732000	-0.30928100	-1.45020300
H	-2.34282200	-0.45962200	-2.47081400
B	-2.05969700	-1.30761400	0.00043400
H	-2.86338600	-2.17350600	0.00072000
B	-1.76733600	-0.30828100	1.45038900
H	-2.34285000	-0.45793600	2.47109400
B	-1.25496300	1.32308100	0.89825900
H	-1.35114200	2.34431400	1.47790900
B	-2.50494600	0.43188200	-0.00015800
H	-3.62694800	0.80205300	-0.00029700
O	1.29905500	1.80451400	-0.00070700
H	2.48556900	-2.55794400	-0.00055700

H	3.07059800	1.22387100	-0.88932200

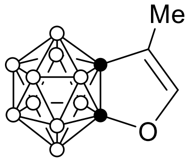

C	0.61002900	-0.44919600	0.00061400
C	0.07090700	1.06783100	-0.00016100
C	3.09071000	-1.34758100	-0.00031600
H	4.10754400	-0.95145300	-0.00087600
H	2.97003300	-1.98437200	-0.88212900
H	2.97131200	-1.98584400	0.88065800
C	2.08988000	-0.23980600	0.00096300
C	2.31260300	1.08088300	-0.00000800
H	3.24290600	1.62711700	-0.00008200
B	-0.11303200	0.15876100	-1.48422800
H	0.64555900	0.42466500	-2.34206700
B	-0.42754500	-1.47486500	-0.89767200
H	0.03935800	-2.38799200	-1.48053100
B	-0.42785700	-1.47467500	0.89822300
H	0.03882300	-2.38771100	1.48138900
B	-0.11351000	0.15922200	1.48452500
H	0.64492800	0.42541200	2.34244600
B	-1.36323400	1.26359500	-0.90186800
H	-1.53125400	2.27724600	-1.47895500
B	-1.75486100	-0.39917000	-1.44737500
H	-2.31642000	-0.59248400	-2.46865200
B	-1.96283900	-1.42136700	-0.00005100
H	-2.69961000	-2.34483100	-0.00006900
B	-1.75521300	-0.39867200	1.44720500
H	-2.31697400	-0.59161800	2.46844000
B	-1.36349200	1.26391400	0.90128600
H	-1.53166100	2.27787800	1.47779900
B	-2.54299600	0.28307100	-0.00035300
H	-3.68966000	0.56801800	-0.00055400
O	1.18790500	1.89426500	-0.00022900

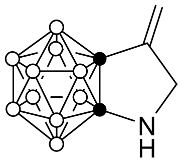

C	0.57045100	-0.53566100	0.01270600
C	0.15624500	1.05316100	-0.04577900
C	2.84839400	-1.58907900	-0.06984300
H	3.92835500	-1.49415500	-0.07191300
C	2.06485800	-0.52078200	0.01839300
C	2.51759200	0.93594000	0.16772000
H	2.88002100	1.10039700	1.18647400
B	-0.09084600	0.04979600	-1.46897200
H	0.67344500	0.22789400	-2.34767400
B	-0.54886700	-1.52681800	-0.82048400
H	-0.14190200	-2.49359000	-1.36045000
B	-0.53046200	-1.42416900	0.96190300
H	-0.11512800	-2.32314500	1.60214200
B	-0.08123700	0.22464000	1.44619200
H	0.68449900	0.45976700	2.30728000
B	-1.26101200	1.27766100	-0.94920000
H	-1.36398400	2.26351900	-1.59049400
B	-1.77433500	-0.37756400	-1.42915900
H	-2.35249900	-0.56843000	-2.44186400
B	-2.06757100	-1.30606000	0.07209100
H	-2.86938000	-2.17277700	0.11852200
B	-1.76923400	-0.23334200	1.46526000
H	-2.33154000	-0.34036400	2.49876400
B	-1.27907100	1.36645400	0.83713100
H	-1.37668900	2.41257700	1.37056300
B	-2.51918900	0.42691600	-0.01441500
H	-3.64278500	0.79212300	-0.03869100
H	2.43870500	-2.58942100	-0.13731400
H	3.33046400	1.18102900	-0.51750100
N	1.35529800	1.82848500	-0.08555300
H	1.43520800	2.29612900	-0.97988900

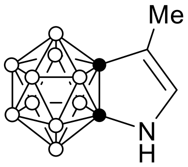

C	0.60937200	-0.43761000	-0.00633800
C	0.06760500	1.08661800	0.03031700
C	3.05239000	-1.41135100	0.00314200
H	4.08321800	-1.05426200	-0.03295100
H	2.88864500	-2.07183100	-0.85413400
H	2.93438600	-2.01927600	0.90613400
C	2.09523800	-0.26488300	-0.00980300
C	2.36827700	1.05335100	-0.00293900
H	3.34775900	1.51071000	-0.01257400
B	-0.11627900	0.18514100	-1.47606500

H	0.63553100	0.47239700	-2.33444200
B	-0.43485000	-1.45446300	-0.92717600
H	0.03495900	-2.35737100	-1.52406600
B	-0.42842200	-1.47974300	0.86643900
H	0.03975400	-2.40180900	1.43451300
B	-0.12622000	0.13934100	1.48818100
H	0.62297400	0.38457000	2.36025500
B	-1.36459000	1.27828800	-0.86974500
H	-1.54006600	2.30314100	-1.43020400
B	-1.75809500	-0.37118400	-1.45566000
H	-2.31711000	-0.53830300	-2.48346800
B	-1.96846000	-1.42359300	-0.03126300
H	-2.70066800	-2.35088400	-0.04989600
B	-1.76810500	-0.43157000	1.43523000
H	-2.32937000	-0.65034500	2.45188200
B	-1.38380600	1.23995100	0.93030200
H	-1.56299300	2.24157800	1.52707000
B	-2.55442200	0.28005800	0.00324900
H	-3.70187600	0.56318200	0.00145600
N	1.24173700	1.89237100	0.08314500
H	1.23165700	2.75403300	-0.44533000

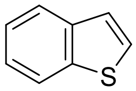

C	-0.06979100	-0.55093300	0.00004700
C	0.10908800	0.85552000	-0.00001400
C	1.41880600	1.37567100	-0.00007600
C	2.49854400	0.50558500	-0.00009300
C	2.30211400	-0.89004500	-0.00004700
C	1.02251800	-1.42969000	0.00001400
C	-2.21368200	0.69974300	0.00003300
C	-1.14535200	1.55014700	-0.00004600
H	1.57508900	2.45274600	-0.00011600
H	3.51082800	0.90245900	-0.00015800
H	3.16315200	-1.55402400	-0.00007500
H	0.87271100	-2.50704600	0.00006100
H	-3.26474300	0.96329100	0.00007200
H	-1.24258400	2.63171700	-0.00010500
S	-1.75924500	-0.97407100	0.00008800

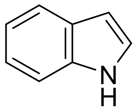

C	-0.24789400	-0.67063700	-0.00000100
C	-0.25231400	0.75245400	-0.00003600
C	0.98114000	1.43179400	0.00006300
C	2.15530100	0.69238300	0.00002600
C	2.13287600	-0.72006200	0.00002000
C	0.93470900	-1.42196000	-0.00004400
C	-2.38893000	0.02665000	-0.00003800
C	-1.62087800	1.16772700	-0.00002400
H	1.01134700	2.51976200	0.00003900
H	3.11375000	1.20607600	0.00009300
H	3.07208200	-1.26821000	-0.00000700
H	0.91891100	-2.51021900	-0.00003500
H	-3.46501000	-0.09120600	-0.00003500
H	-1.99677100	2.18282400	-0.00008900
N	-1.56305600	-1.07849600	0.00002600
H	-1.87698500	-2.03964300	0.00007700

Table S5. Selected experimental and calculated structural parameters of **8** (calculated at the B3LYP/6-311+G(d,p) level of theory)

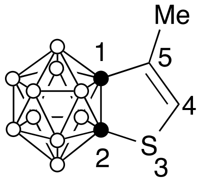

8

Bond Length (Å)	Experimental	Computational
C(1)-C(2)	1.634(2)	1.629
C(2)-S(3)	1.786(2)	1.809
S(3)-C(4)	1.756(2)	1.770
C(4)-C(5)	1.349(3)	1.341
C(5)-C(1)	1.490(2)	1.495
Bond Angle (^{o})	Experimental	Computational
C(1)-C(2)-S(3)	108.3(2)	108.3
C(2)-S(3)-C(4)	92.0(1)	91.4
S(3)-C(4)-C(5)	118.5(2)	118.5
C(4)-C(5)-C(1)	113.0(2)	113.6
C(5)-C(1)-C(2)	108.2(2)	108.3

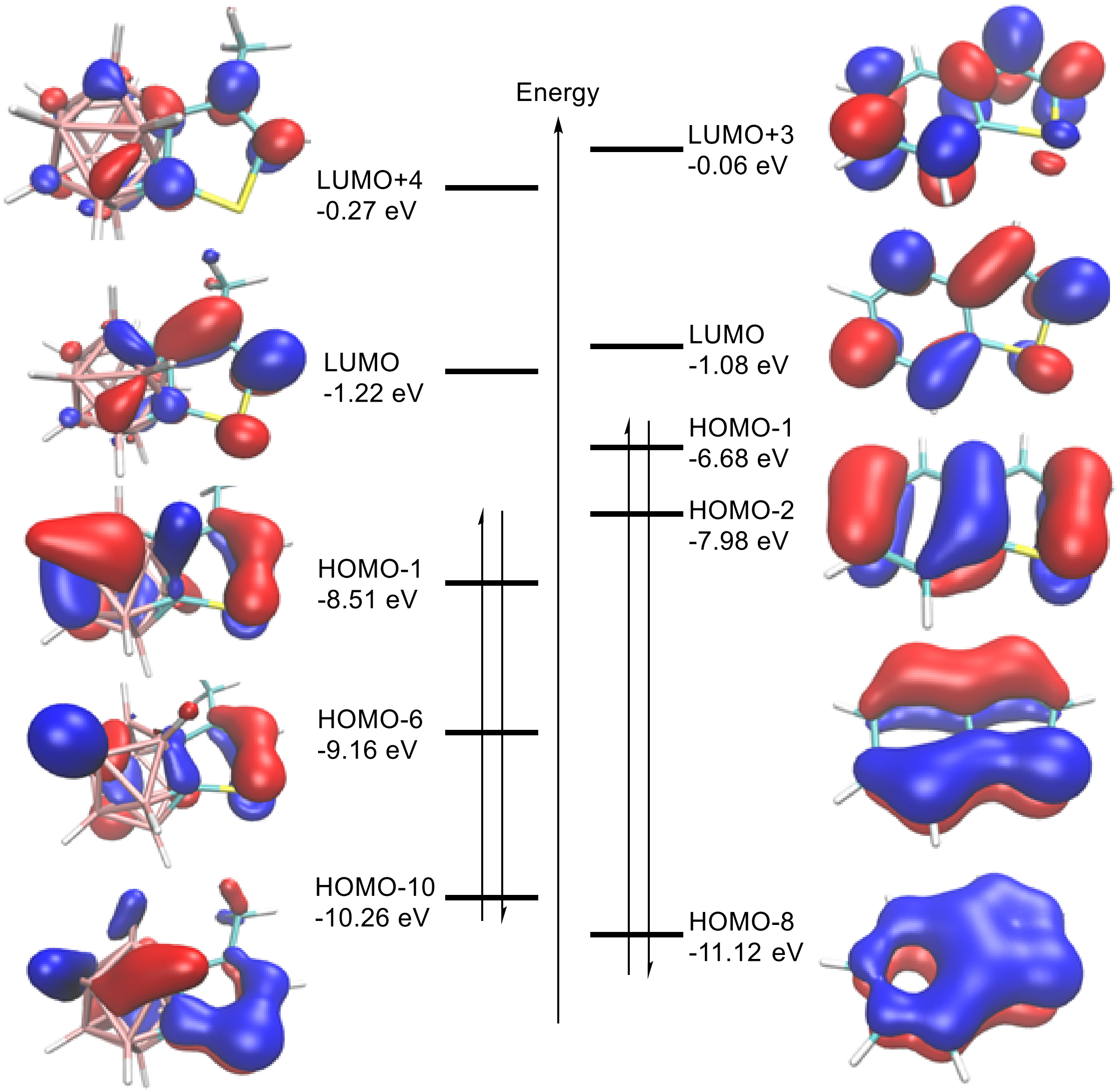

Figure S11. Plots of the LUMO+4, LUMO, HOMO-1, HOMO-6 and HOMO-8 orbitals of **8** (left) and the LUMO+3, LUMO, HOMO-1, HOMO-2 and HOMO-8 of benzothiophene (right) calculated at the B3LYP/6-311++G(d,p) level of theory, where red and blue indicate different phases of the wave functions.

References

1. H. Nakamura, K. Aoyagi, and Y. Yamamoto, *J. Am. Chem. Soc.* 1998,**120**, 1167-1171.
2. A.-R. Popescu, A. D. Musteti, A. Ferrer-Ugalde, C. Viñas, R. Núñez, and F. Teixidor, *Chem. Eur. J.* 2012, **18**, 3174-3184.
3. K. Ohta, T. Goto, H. Yamazaki, F. Pichierri, and Y. Endo, *Inorg. Chem.* 2007, **46**, 3966-3970.
4. R. Cheng, J. Zhang, J. Zhang, Z. Qiu and Z. Xie, *Angew., Chem. Int. Ed.* 2016, 55, 1751-1754.
5. G. M. Sheldrick, SADABS: Program for Empirical Absorption Correction of Area Detector Data. University of Göttingen: Germany, 1996.
6. G. M. Sheldrick, SHELXTL 5.10 for Windows NT: Structure Determination Software Programs. Bruker Analytical X-ray Systems, Inc., Madison, Wisconsin, USA, 1997.
7. M. J. Frisch, G. W. Trucks, H. B. Schlegel, G. E. Scuseria, M. A. Robb, J. R. Cheeseman, G. Scalmani, V. Barone, B. Mennucci, G. A. Petersson, H. Nakatsuji, M. Caricato, X. Li, H. P. Hratchian, A. F. Izmaylov, J. Bloino, G. Zheng, J. L. Sonnenberg, M. Hada, M. Ehara, K. Toyota, R. Fukuda, J. Hasegawa, M. Ishida, T. Nakajima, Y. Honda, O. Kitao, H. Nakai, T. Vreven, J. A. Montgomery, Jr., J. E. Peralta, F. Ogliaro, M. Bearpark, J. J. Heyd, E. Brothers, K. N. Kudin, V. N. Staroverov, R. Kobayashi, J. Normand, K. Raghavachari, A. Rendell, J. C. Burant, S. S. Iyengar, J. Tomasi, M. Cossi, N. Rega, J. M. Millam, M. Klene, J. E. Knox, J. B. Cross,V. Bakken, C. Adamo, J. Jaramillo, R. Gomperts, R. E. Stratmann, O.Yazyev, A. J. Austin, R. Cammi, C. Pomelli, J. W. Ochterski, R. L. Martin,K. Morokuma, V. G. Zakrzewski, G. A. Voth, P. Salvador, J. J.Dannenberg, S. Dapprich, A. D. Daniels, Ö. Farkas, J. B. Foresman, J. V. Ortiz, J. Cioslowski and D. J. Fox, Gaussian 09, Revision D.01; Gaussian, Inc.: Pittsburgh, PA, 2009.
8. (a) A. D. Becke, *J. Chem. Phys.*, 1993, **98**, 5648-5652; (b) C. Lee, W. Yang and R. G. Parr, *Phys. Rev. B,* 1988, **37**, 785-789.
9. (a) J. Zhu, K. An and P. v. R. Schleyer, *Org. Lett.* 2013, **15**, 2442-2445; (b) C. Zhu, S. Li, M. Luo, X. Zhou, Y. Niu, M. Lin, J. Zhu, Z. Cao, X. Lu, T. Wen, Z. Xie, P. v. R. Schleyer, and H. Xia, *Nat. Chem.* 2013, **5**, 698-703.
10. (a) E. Runge, and E. K. U. Gross, *Phys. Rev. Lett.* 1984, **52**, 997-1000; b) E. K. U. Gross and W. Kohn, *Adv. Quantum Chem.* 1990, **21**, 255-291; c) E. K. U. Gross, C. A. Ullrich and U. J. Gossmann, in *Density Functional Theory*, ed. E. K. U. Gross and R. M. Driezler, Plenum Press, New York, 1995, 149-171; d) M. E. Casida, in *Recent Advances in Density Functional Methods, Part I*, ed. D. P. Chong, World Scientific, Singapore, 1995, pp. 155-193.

11. W. Humphrey, A. Dalke and K. Schulten, *J. Mol. Graphics* 1996, **14**, 33-38.

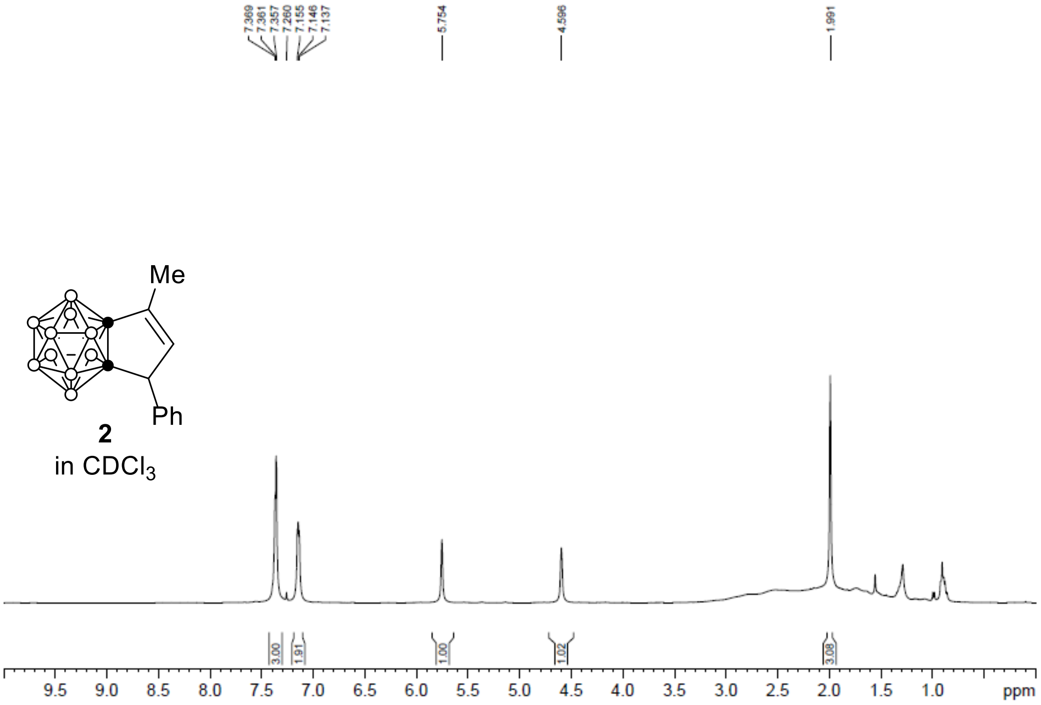

Figure S12. ^{1}H NMR spectrum of **2** in $CDCl_3$.

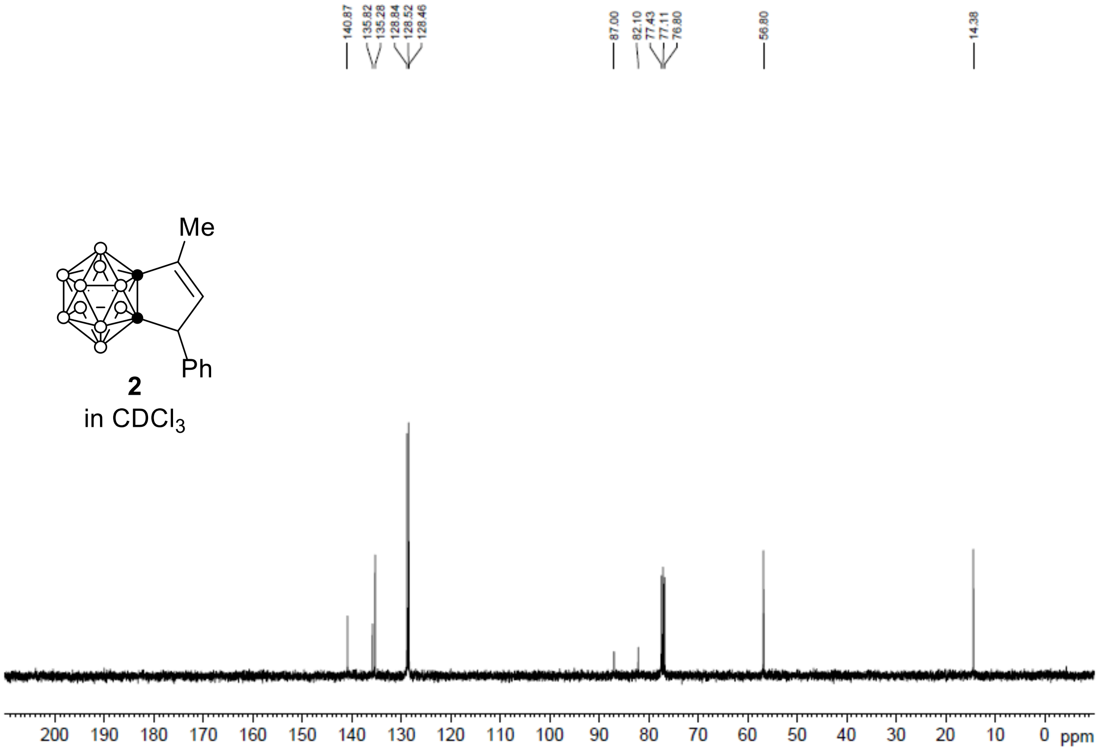

Figure S13. ^{13}C{^{1}H} NMR spectrum of **2** in $CDCl_3$.

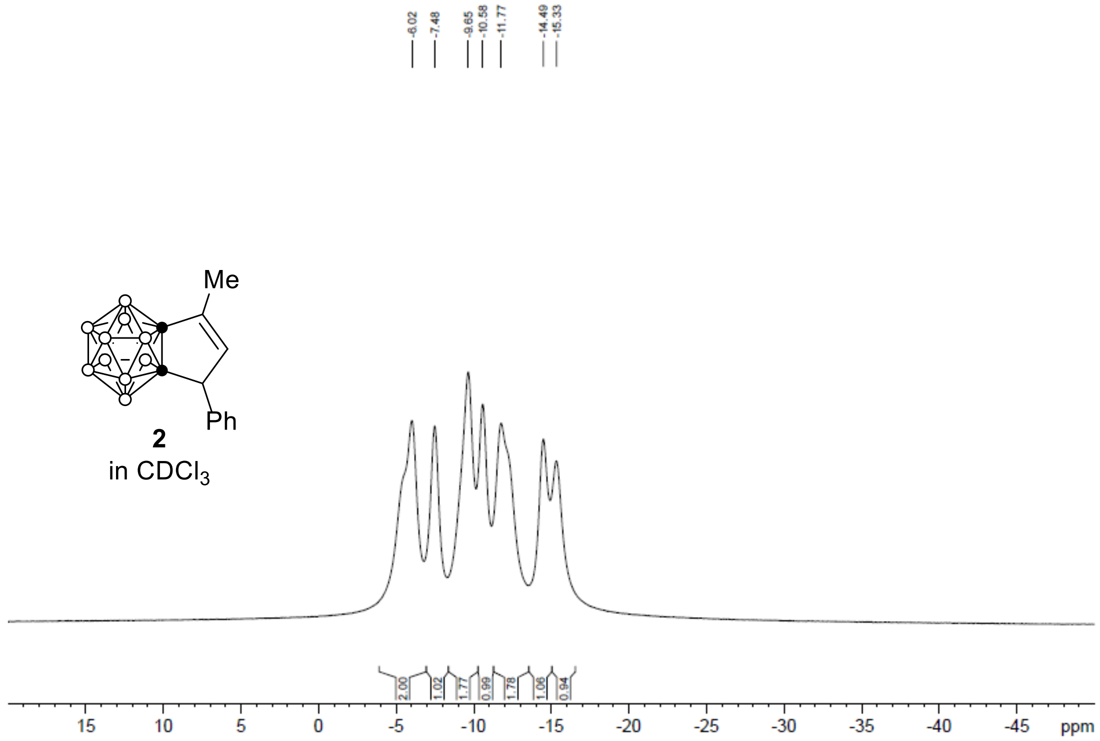

Figure S14. $^{11}B\{^{1}H\}$ NMR spectrum of **2** in $CDCl_3$.

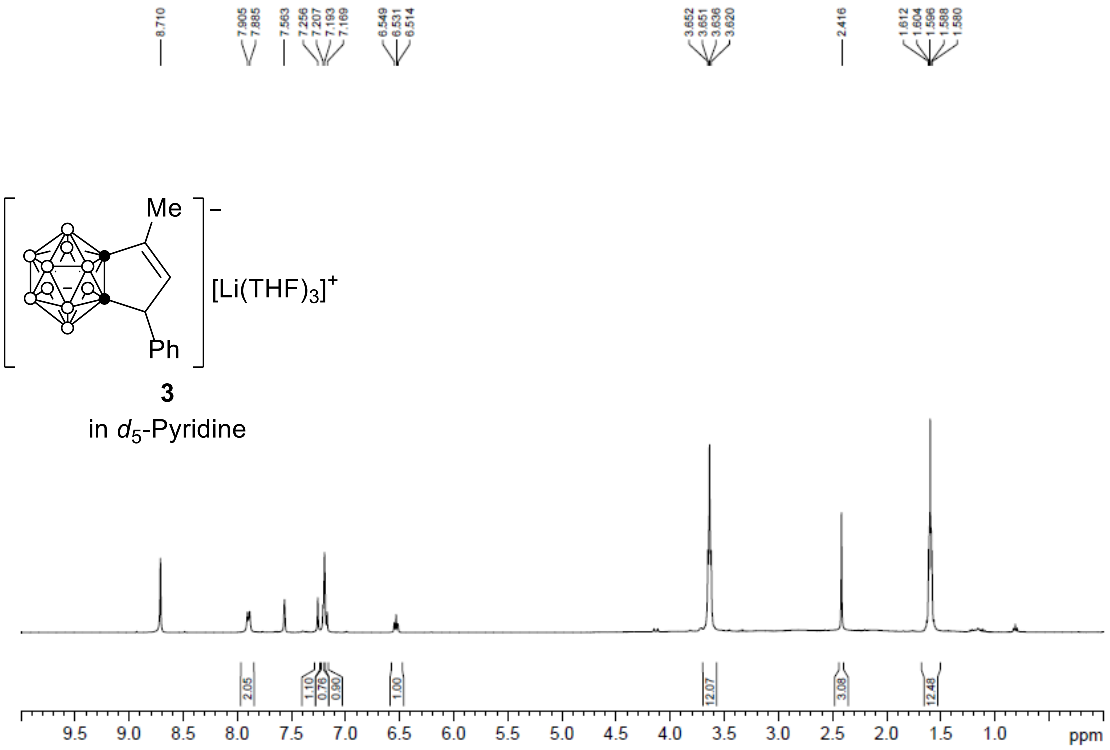

Figure S15. ^{1}H NMR spectrum of **3** in d_5-pyridine.

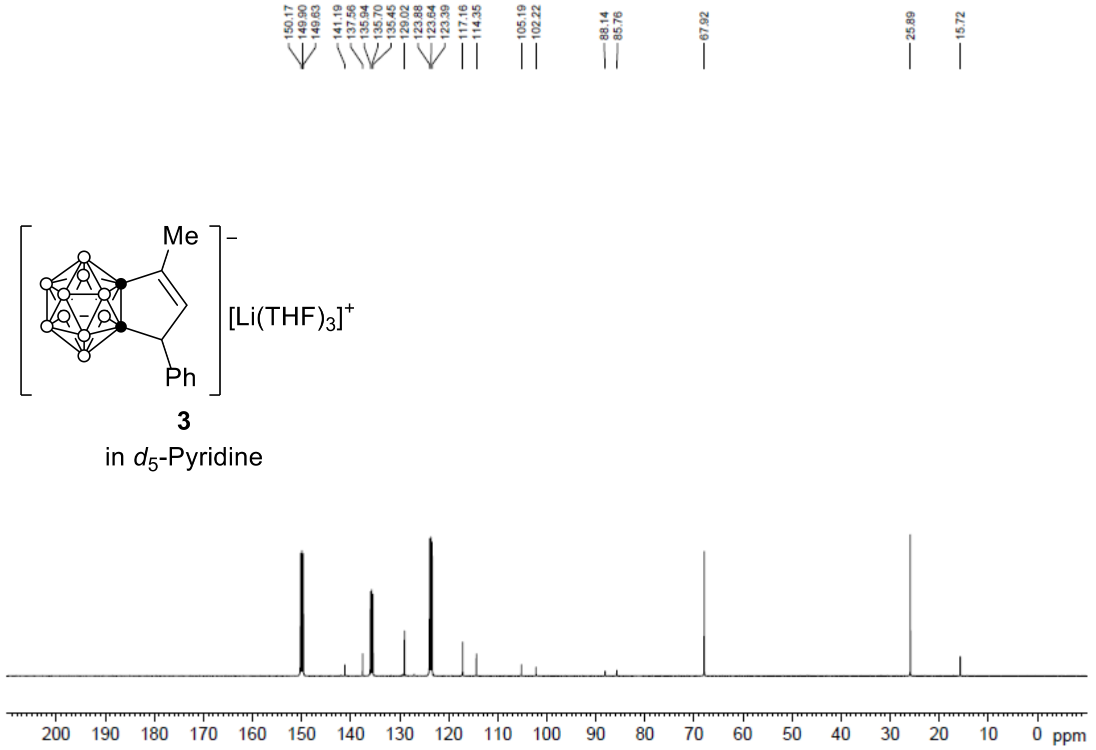

Figure S16. $^{13}C\{^{1}H\}$ NMR spectrum of **3** in d_5-pyridine.

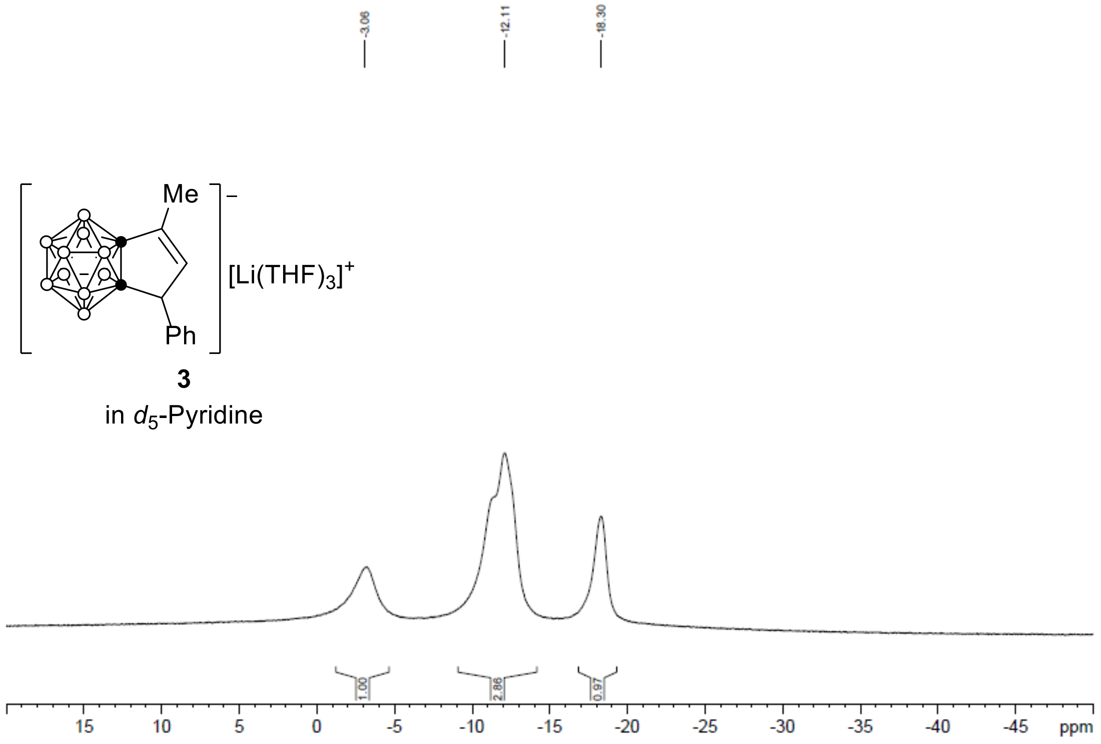

Figure S17. $^{11}B\{^{1}H\}$ NMR spectrum of **3** in d_5-pyridine.

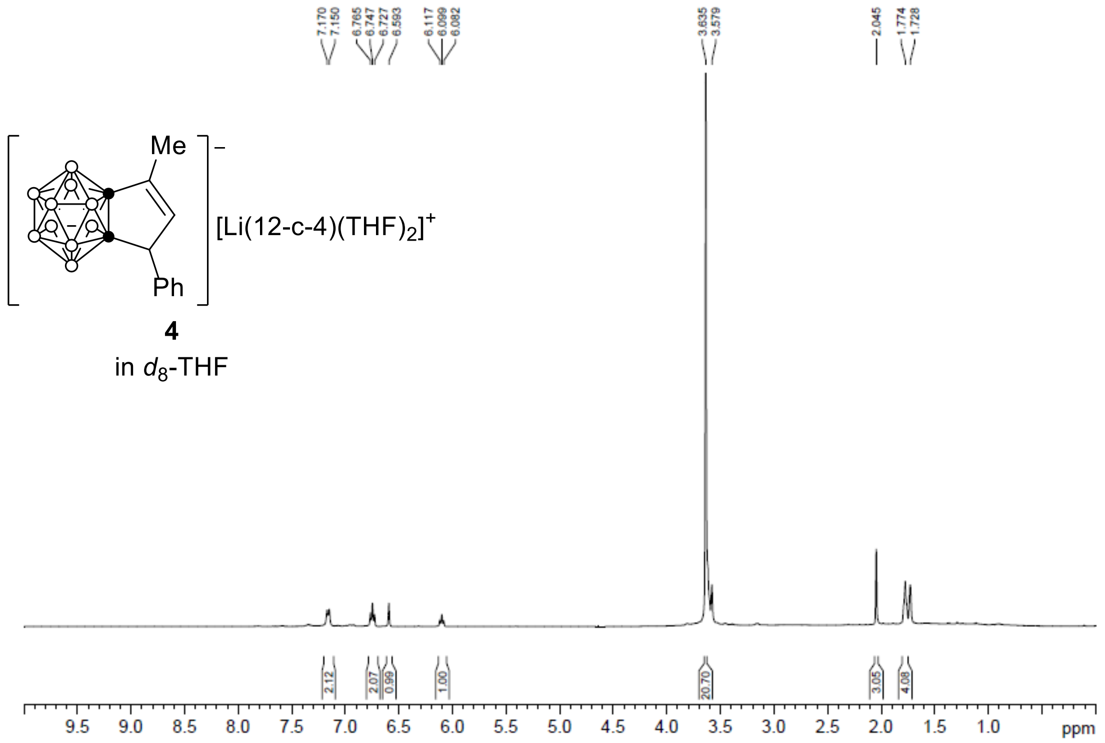

Figure S18. ^{1}H NMR spectrum of **4** in d_8-THF.

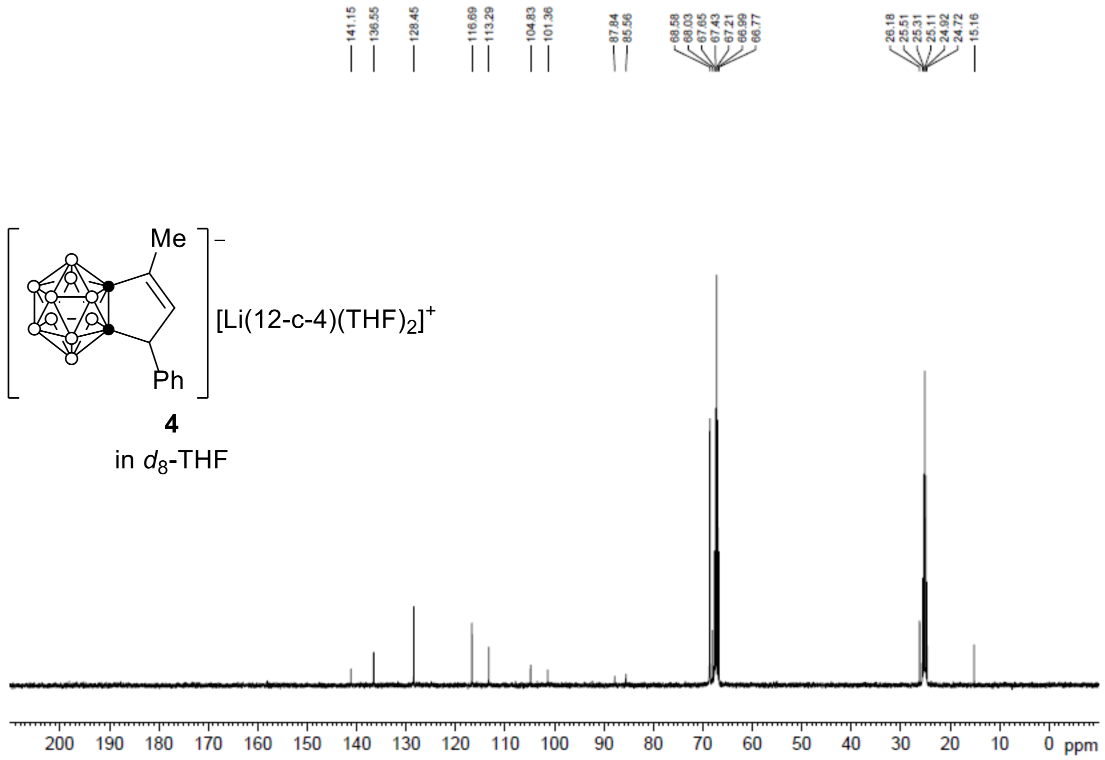

Figure S19. $^{13}C\{^{1}H\}$ NMR spectrum of **4** in d_8-THF.

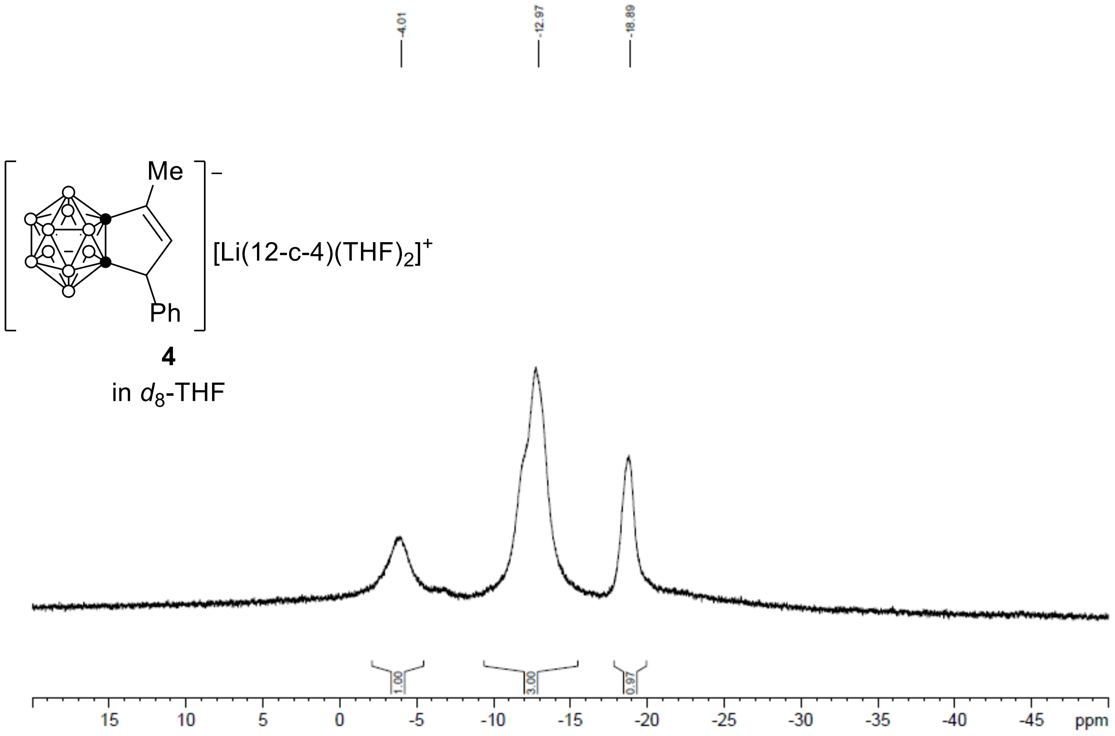

Figure S20. $^{11}B\{^{1}H\}$ NMR spectrum of **4** in d_8-THF.

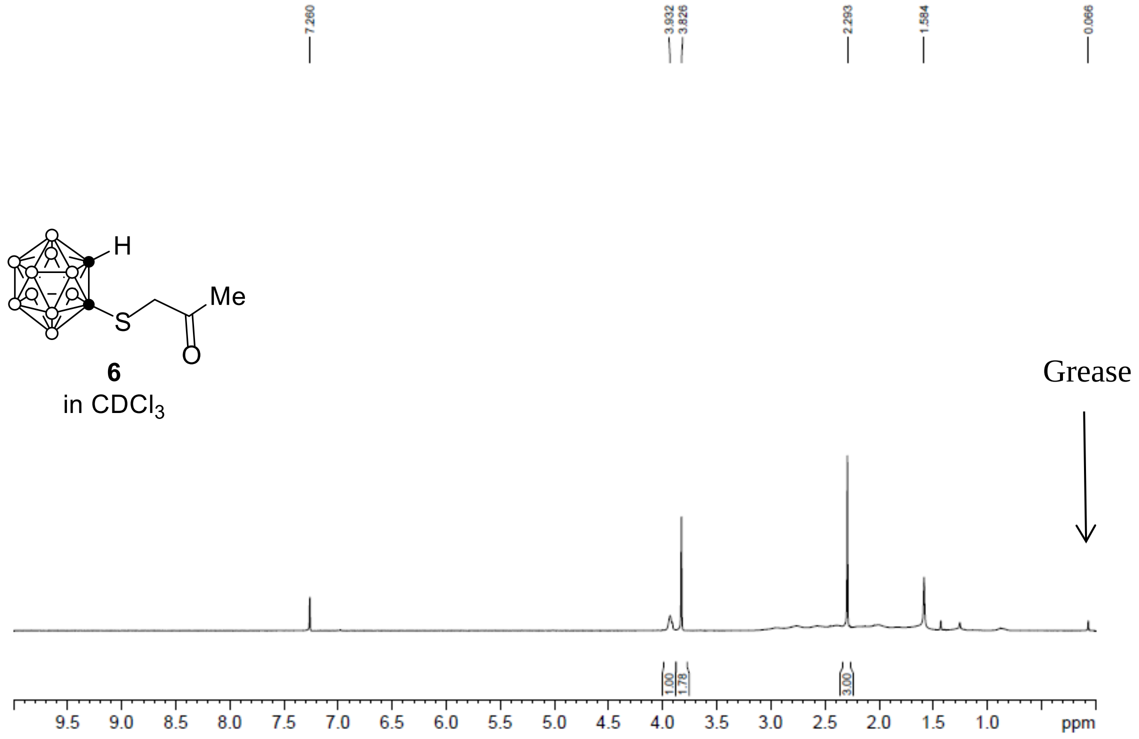

Figure S21. ^{1}H NMR spectrum of **6** in $CDCl_3$.

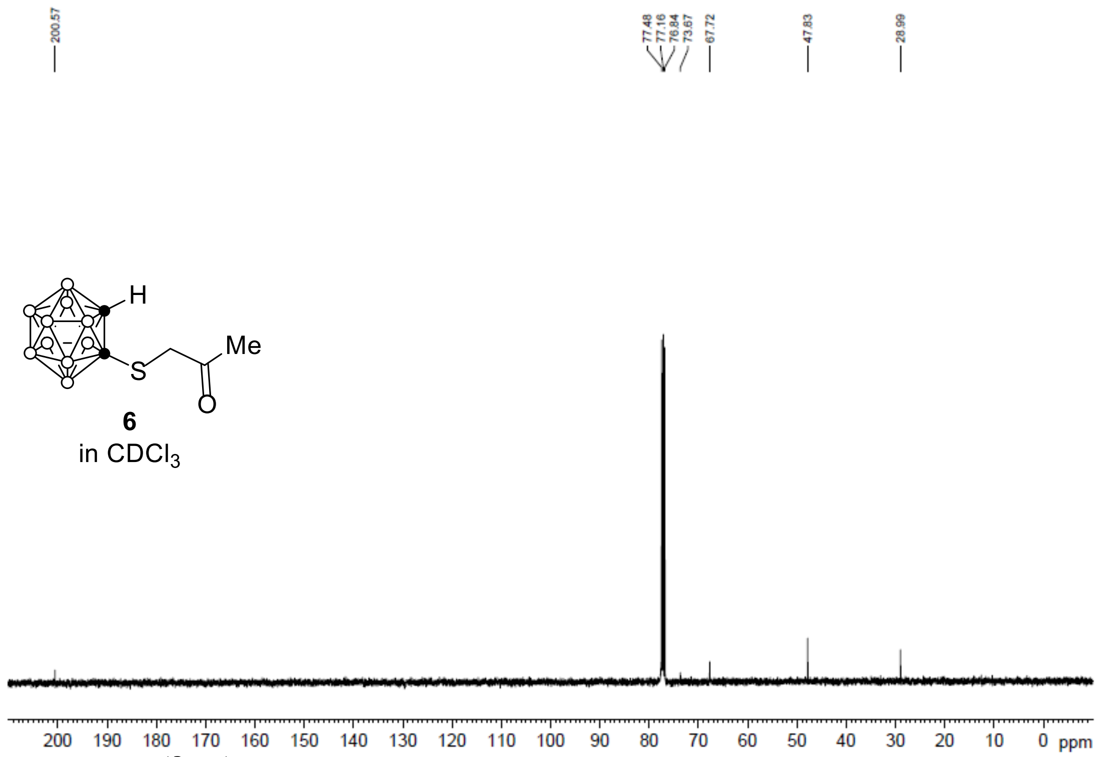

Figure S22. $^{13}C\{^1H\}$ NMR spectrum of **6** in $CDCl_3$.

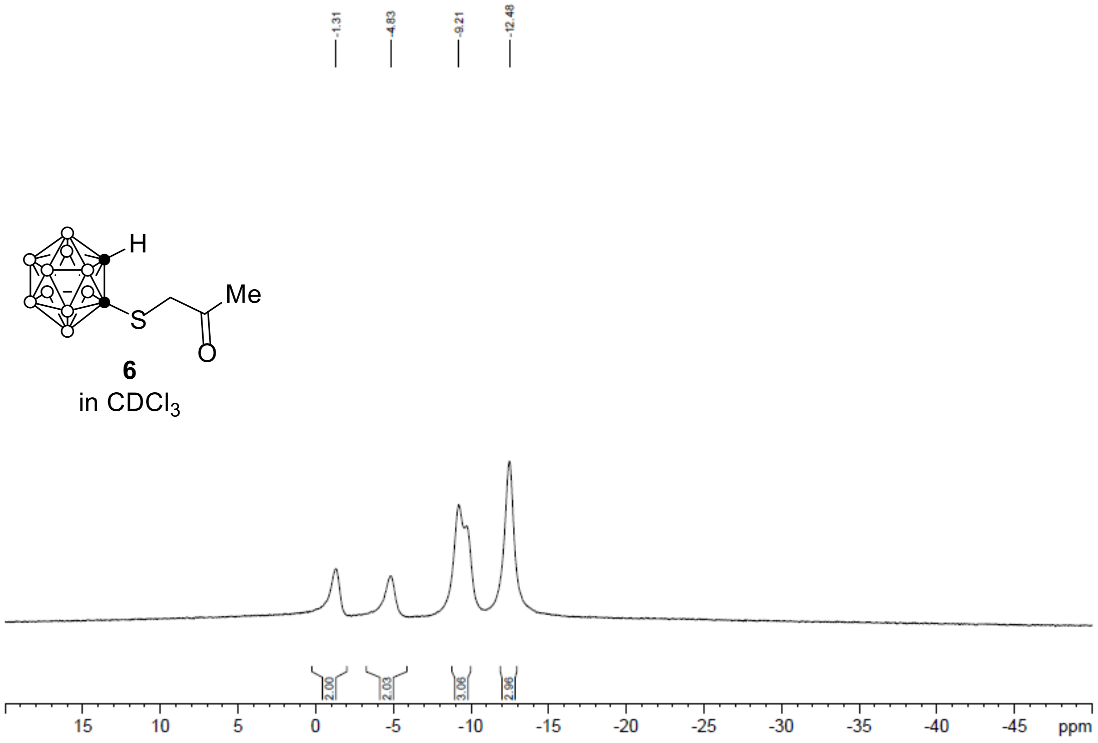

Figure S23. $^{11}B\{^1H\}$ NMR spectrum of **6** in $CDCl_3$.

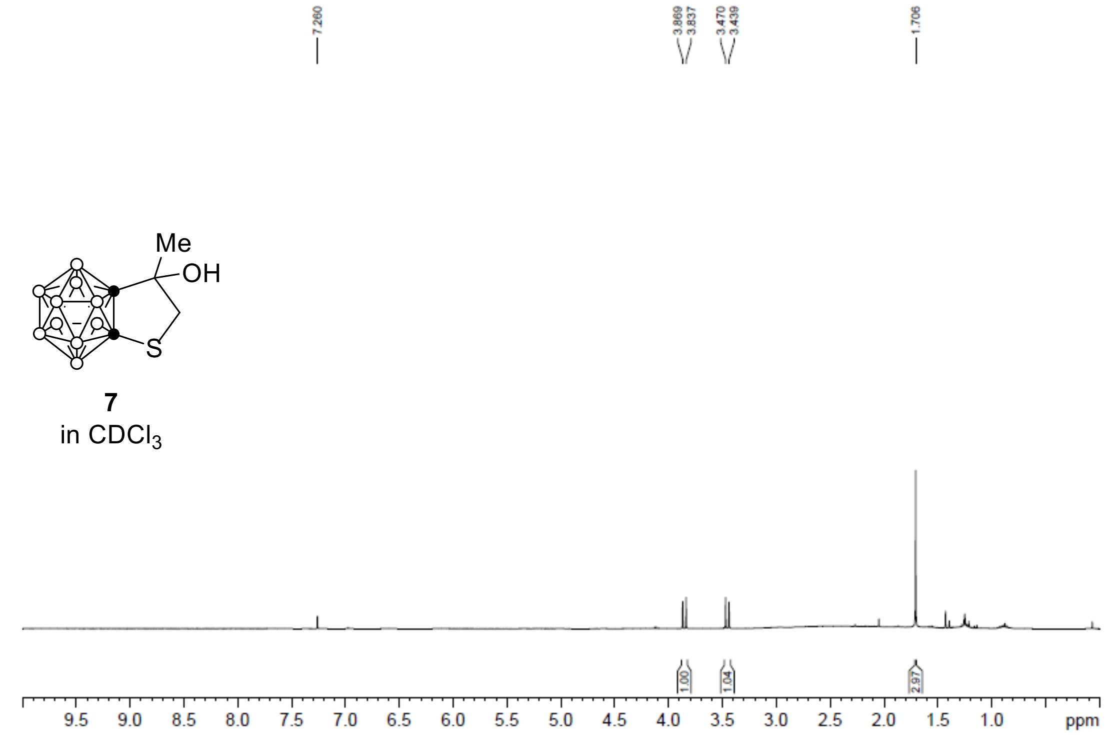

Figure S24. ^{1}H NMR spectrum of **7** in $CDCl_3$.

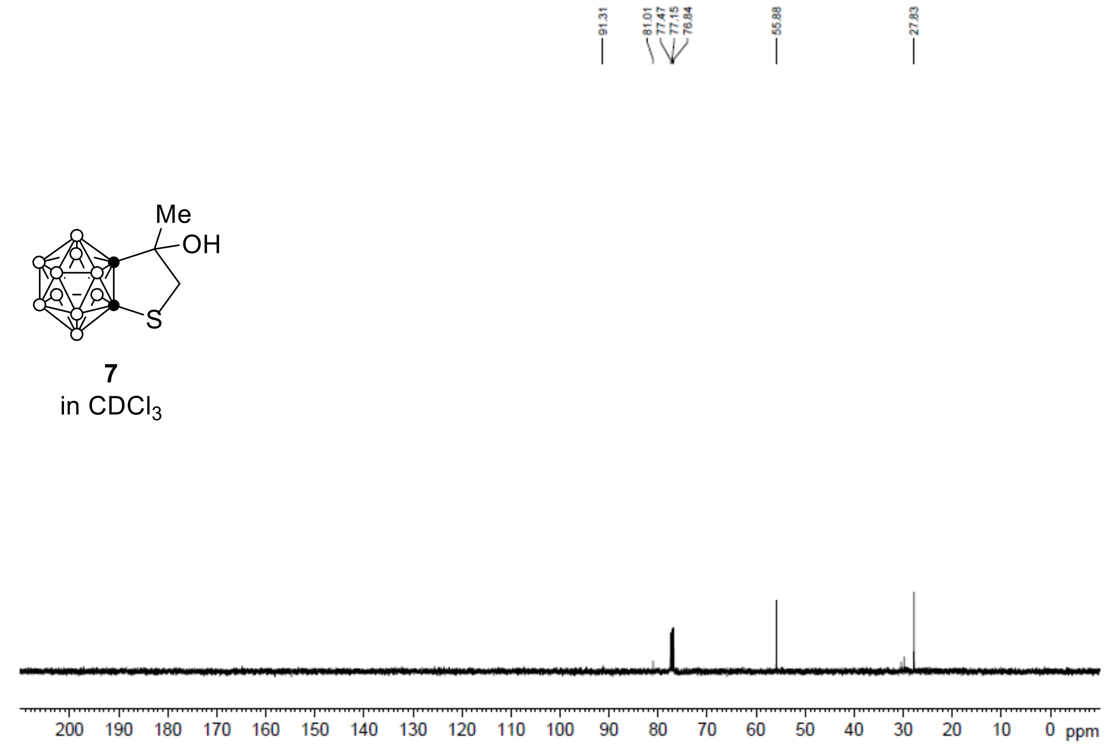

Figure S25. ^{13}C{^{1}H} NMR spectrum of **7** in $CDCl_3$.

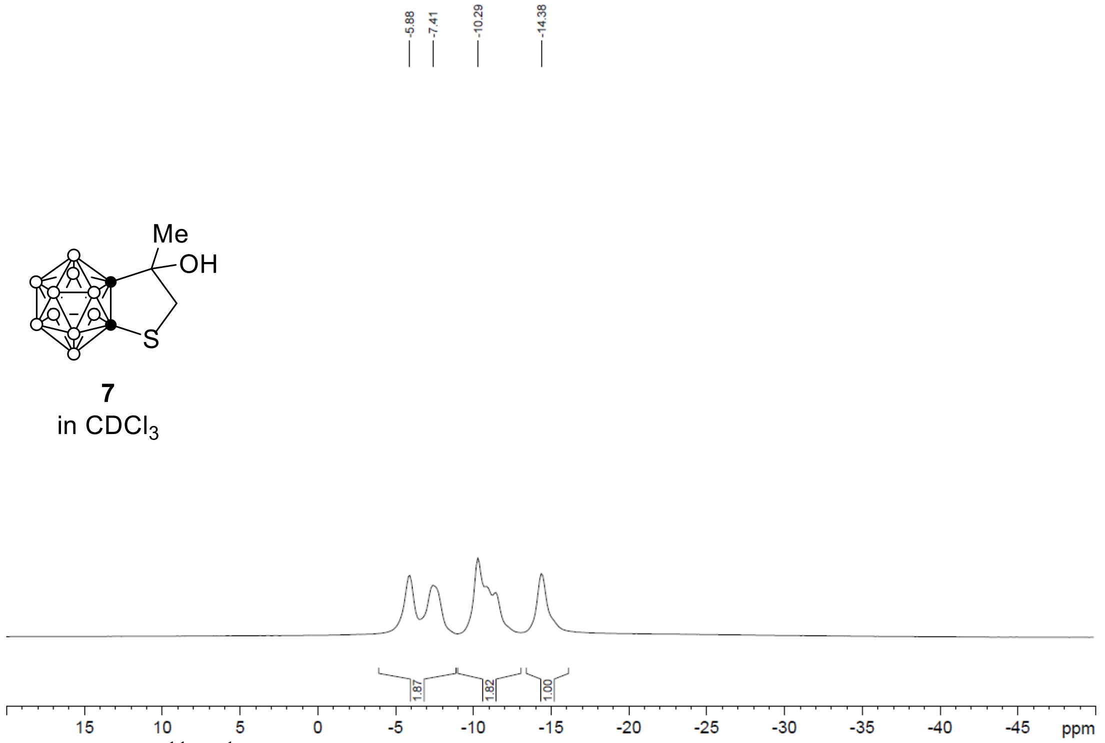

Figure S26. $^{11}B\{^{1}H\}$ NMR spectrum of **7** in $CDCl_3$.

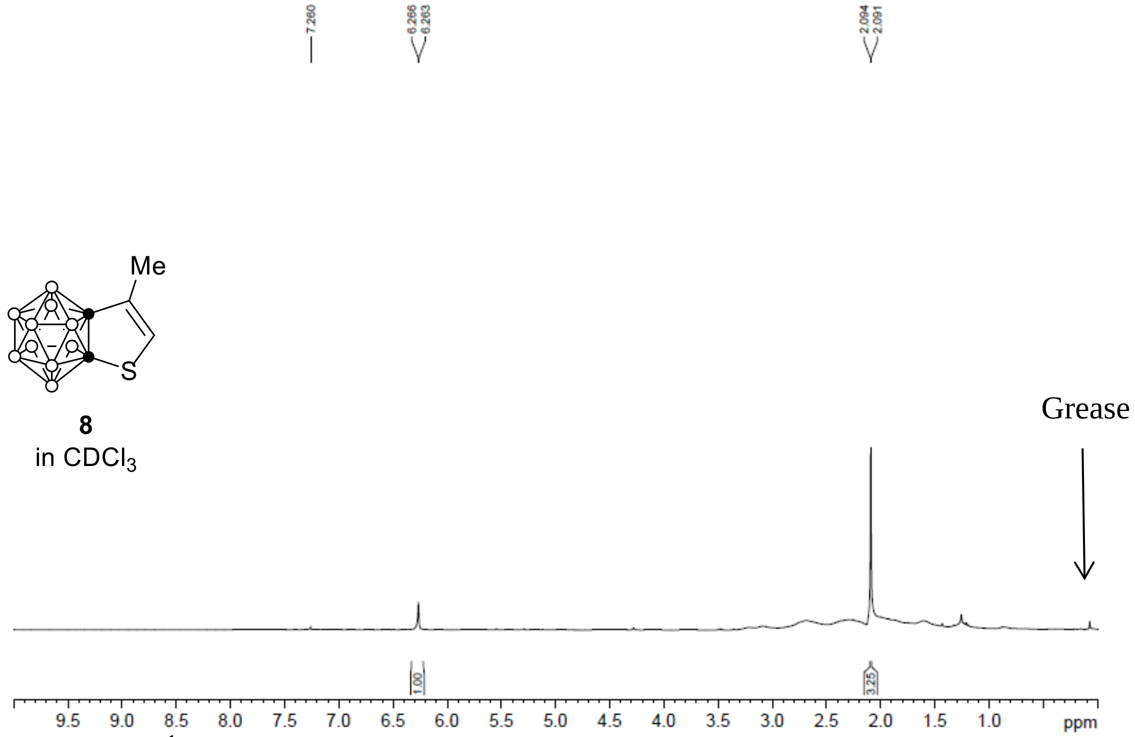

Figure S27. ^{1}H NMR spectrum of **8** in $CDCl_3$.

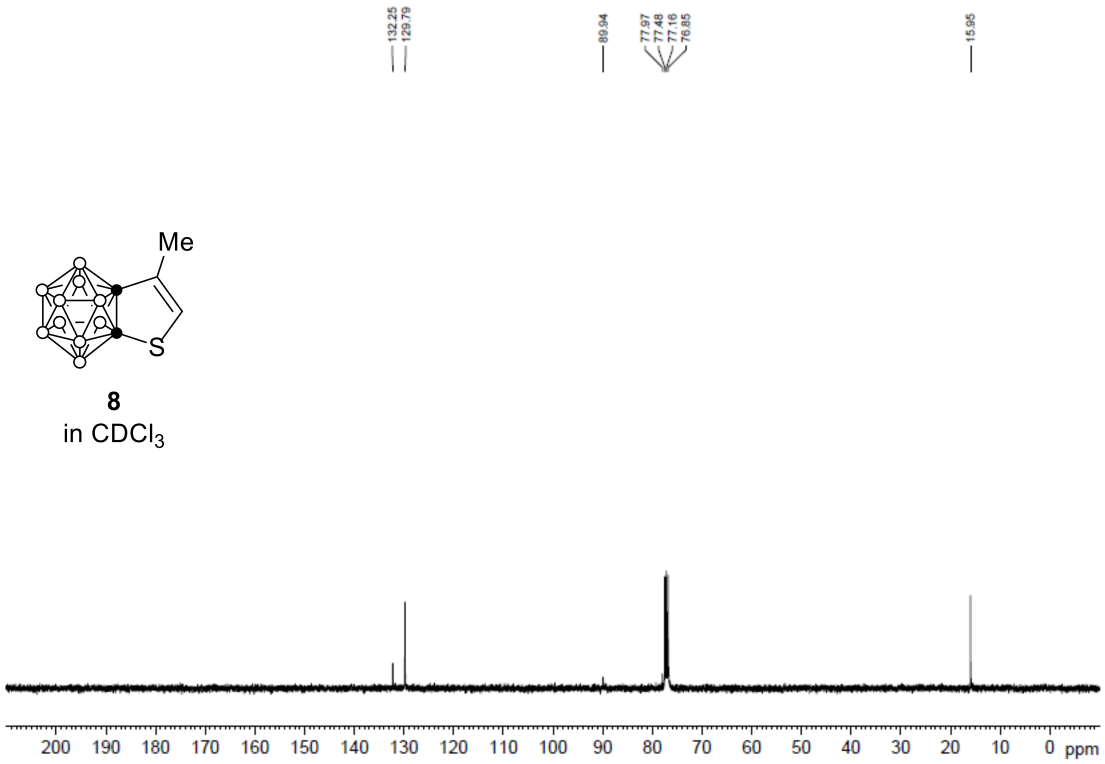

Figure S28. $^{13}C\{^1H\}$ NMR spectrum of **8** in $CDCl_3$.

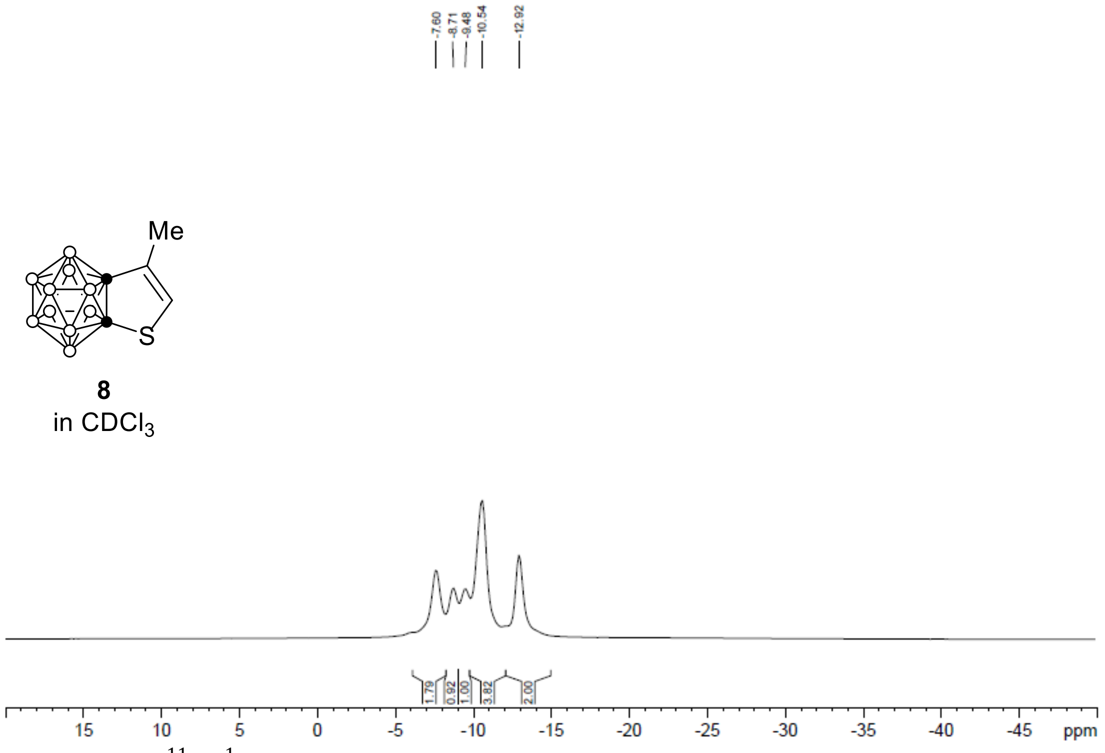

Figure S29. $^{11}B\{^1H\}$ NMR spectrum of **8** in $CDCl_3$.

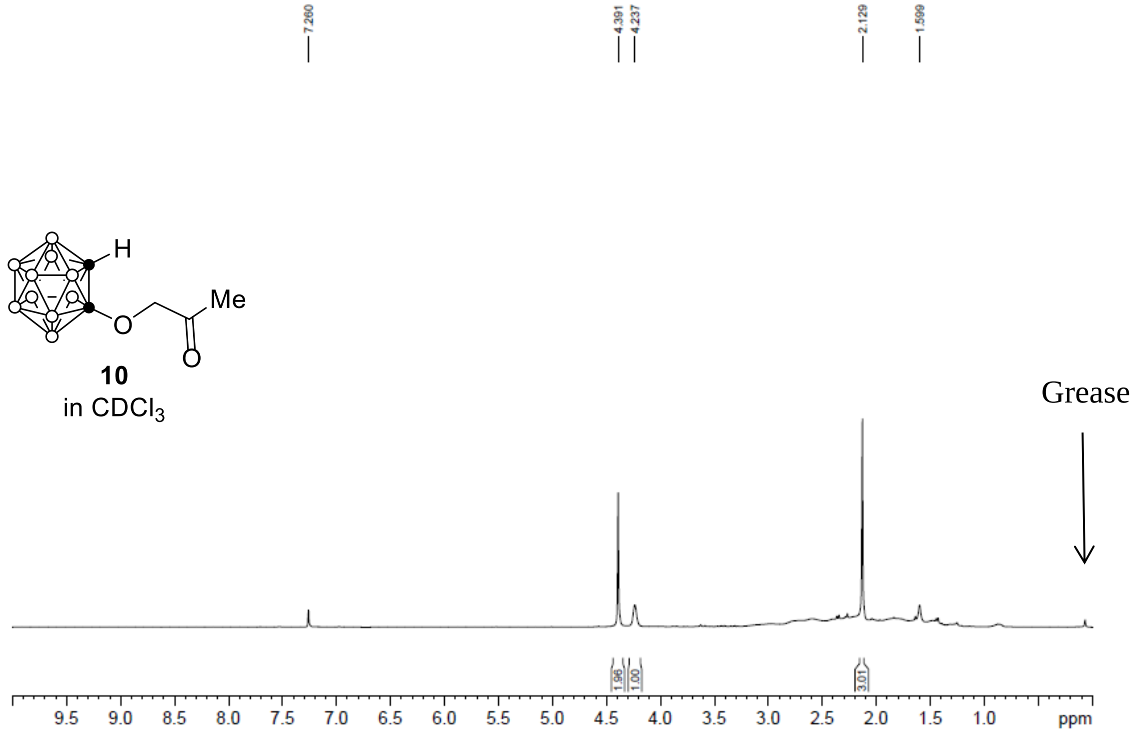

Figure S30. ^{1}H NMR spectrum of **10** in $CDCl_3$.

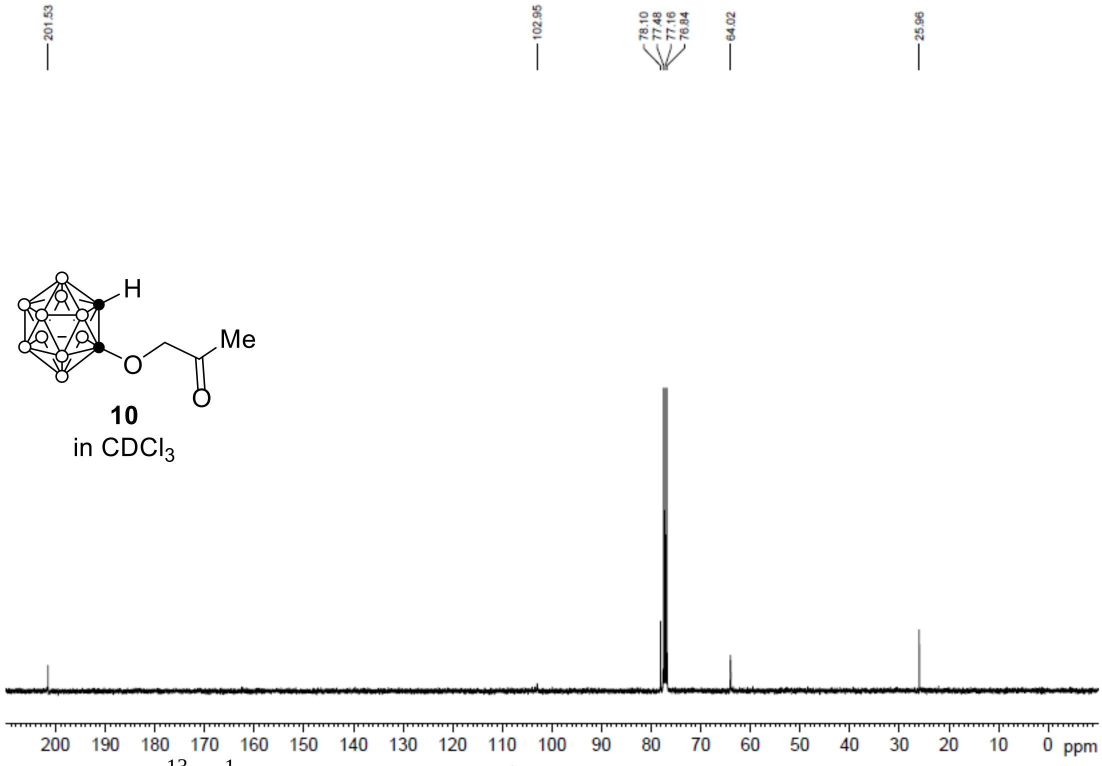

Figure S31. ^{13}C{^{1}H} NMR spectrum of **10** in $CDCl_3$.

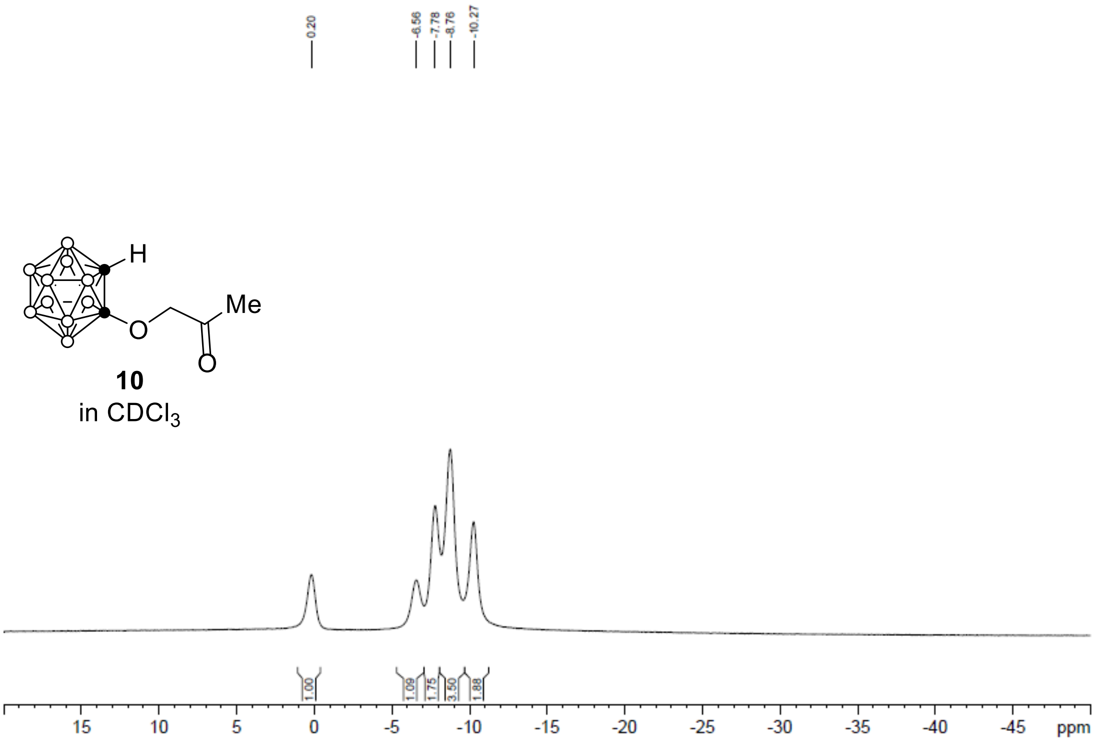

Figure S32. $^{11}B\{^{1}H\}$ NMR spectrum of **10** in $CDCl_3$.

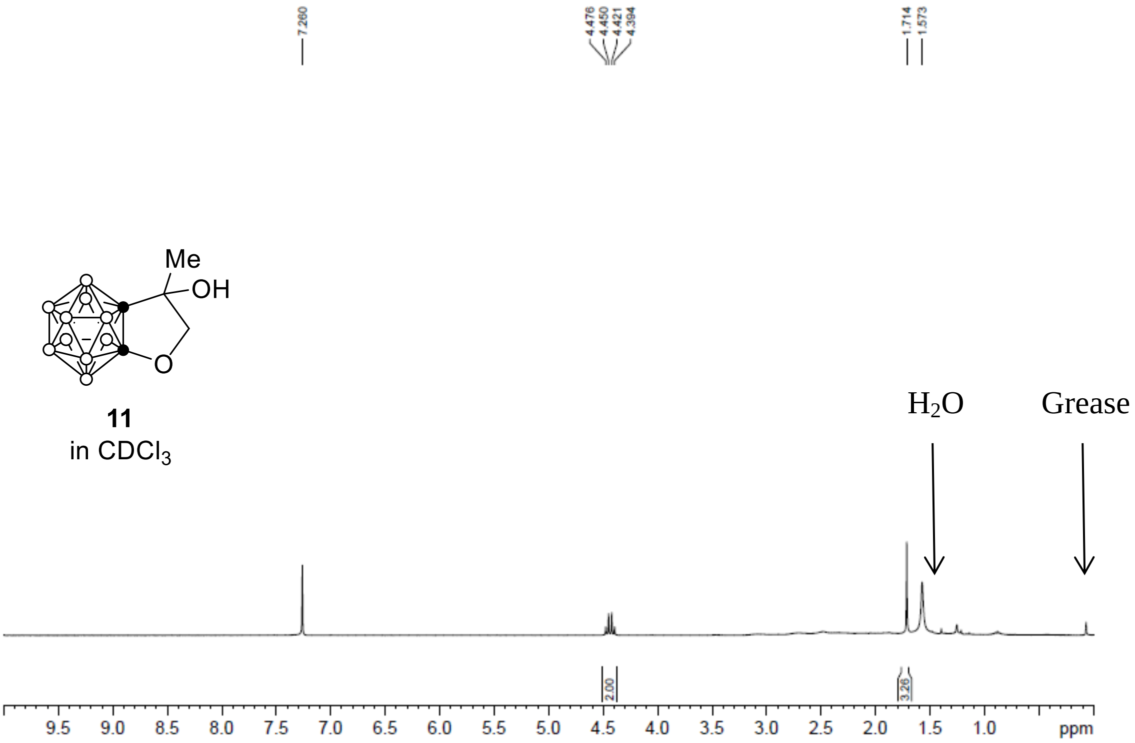

Figure S33. ^{1}H NMR spectrum of **11** in $CDCl_3$.

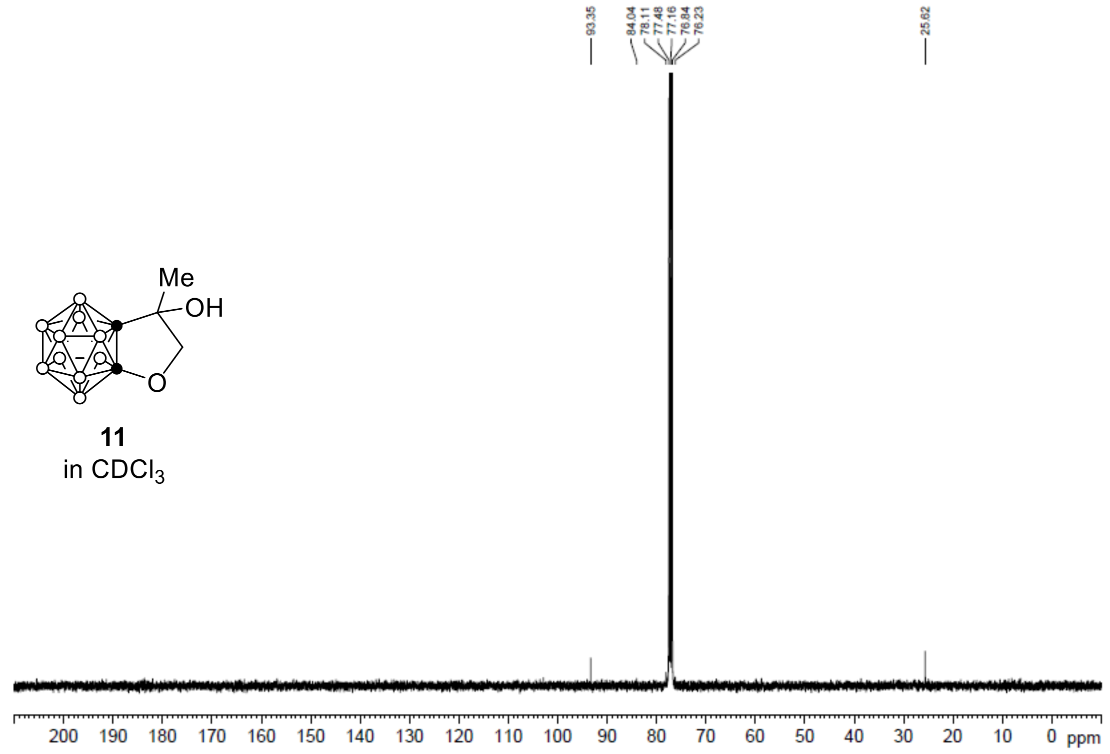

Figure S34. $^{13}C\{^{1}H\}$ NMR spectrum of **11** in $CDCl_3$.

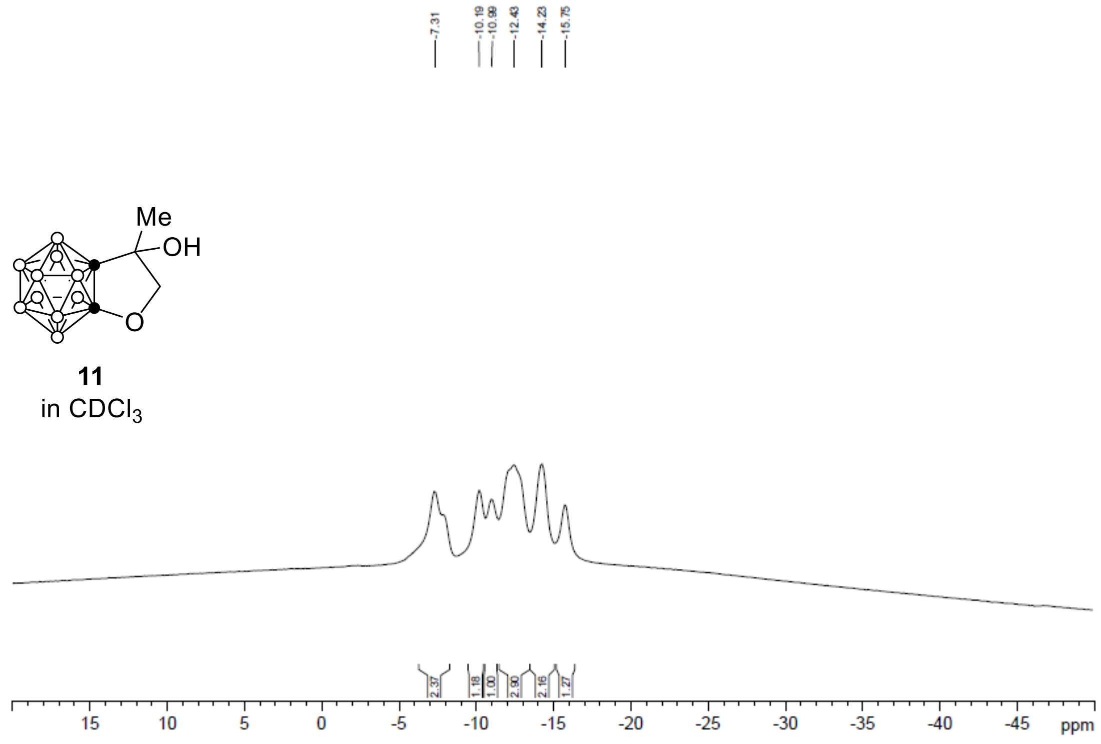

Figure S35. $^{11}B\{^{1}H\}$ NMR spectrum of **11** in $CDCl_3$.

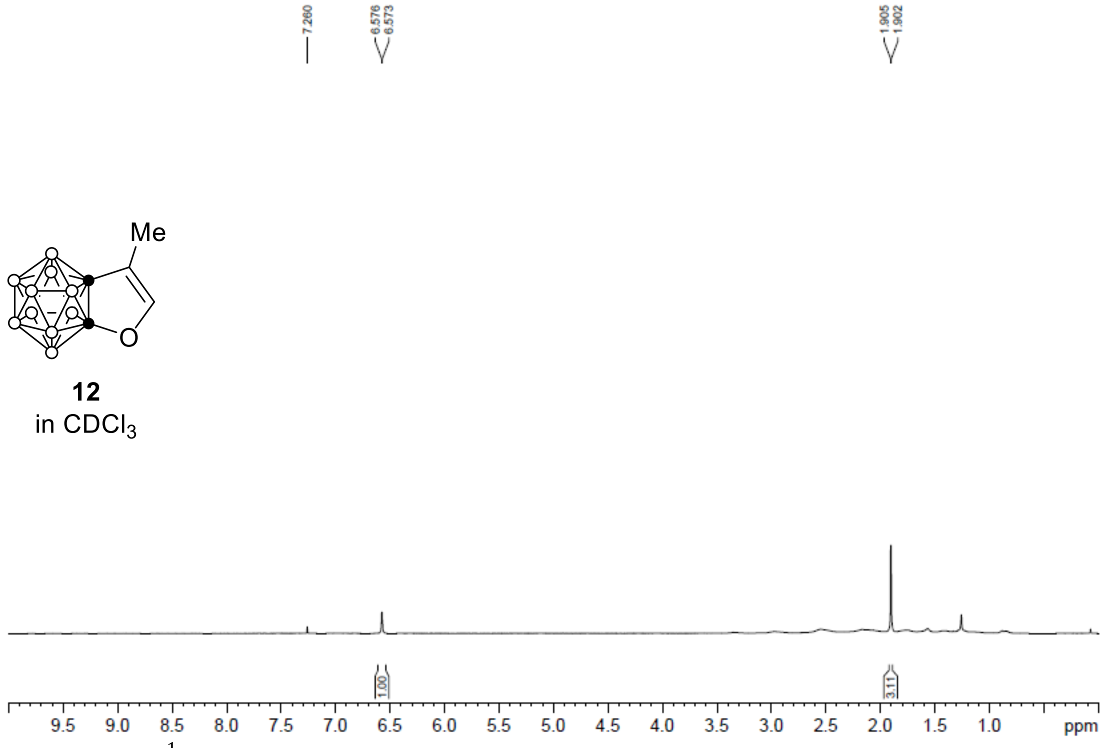

Figure S36. ^{1}H NMR spectrum of **12** in $CDCl_3$.

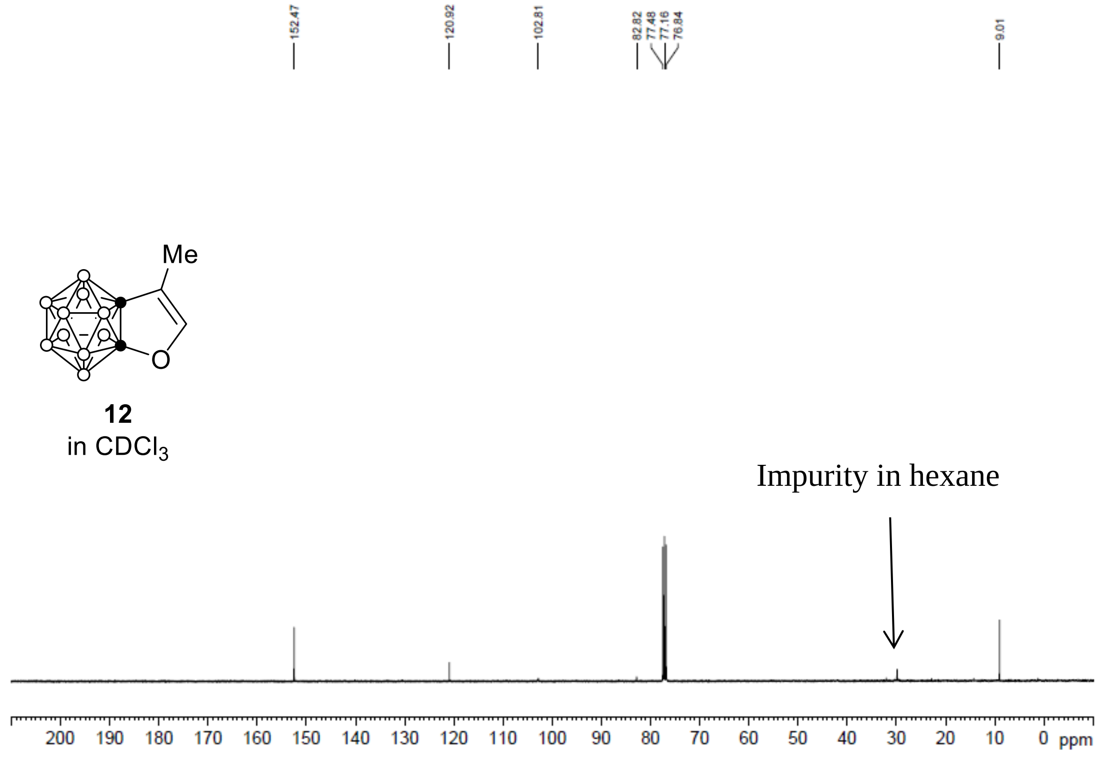

Figure S37. $^{13}C\{^{1}H\}$ NMR spectrum of **12** in $CDCl_3$.

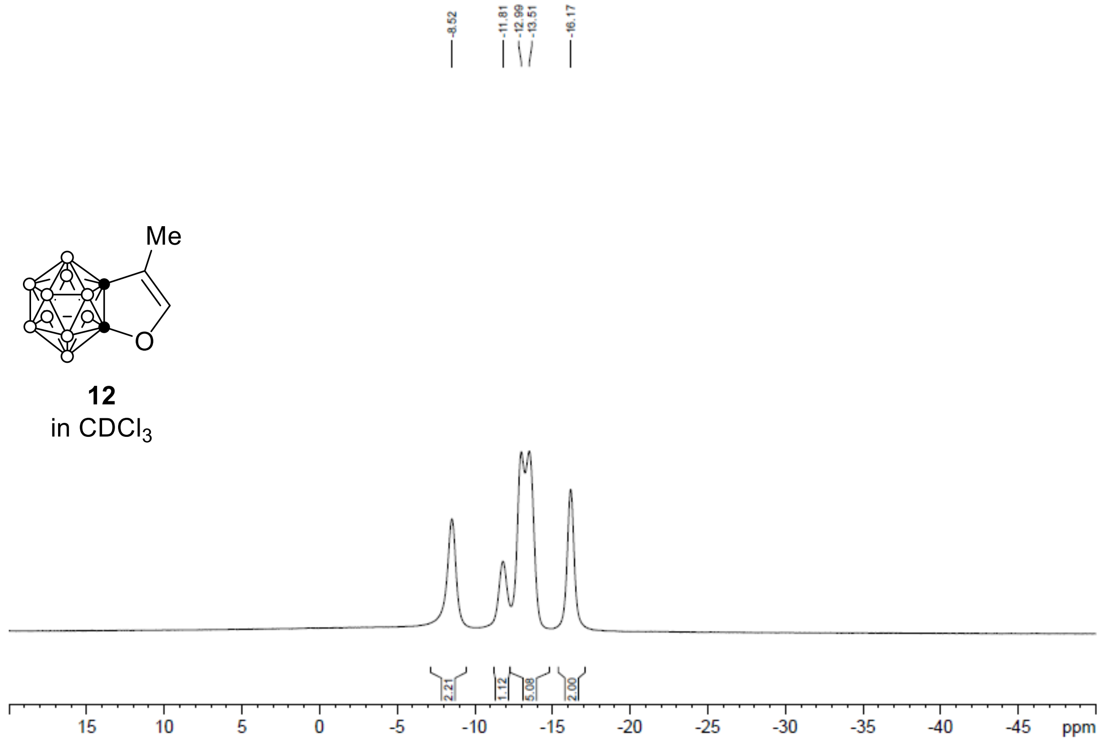

Figure S38. $^{11}B\{^{1}H\}$ NMR spectrum of **12** in $CDCl_3$.

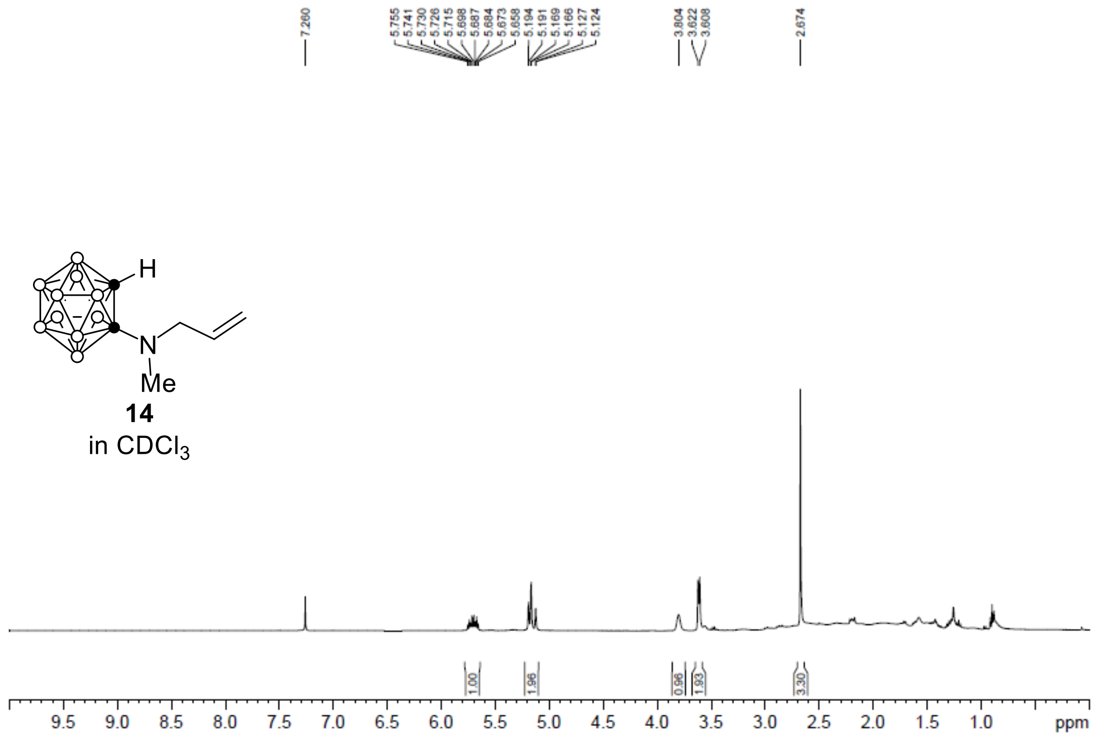

Figure S39. ^{1}H NMR spectrum of **14** in $CDCl_3$.

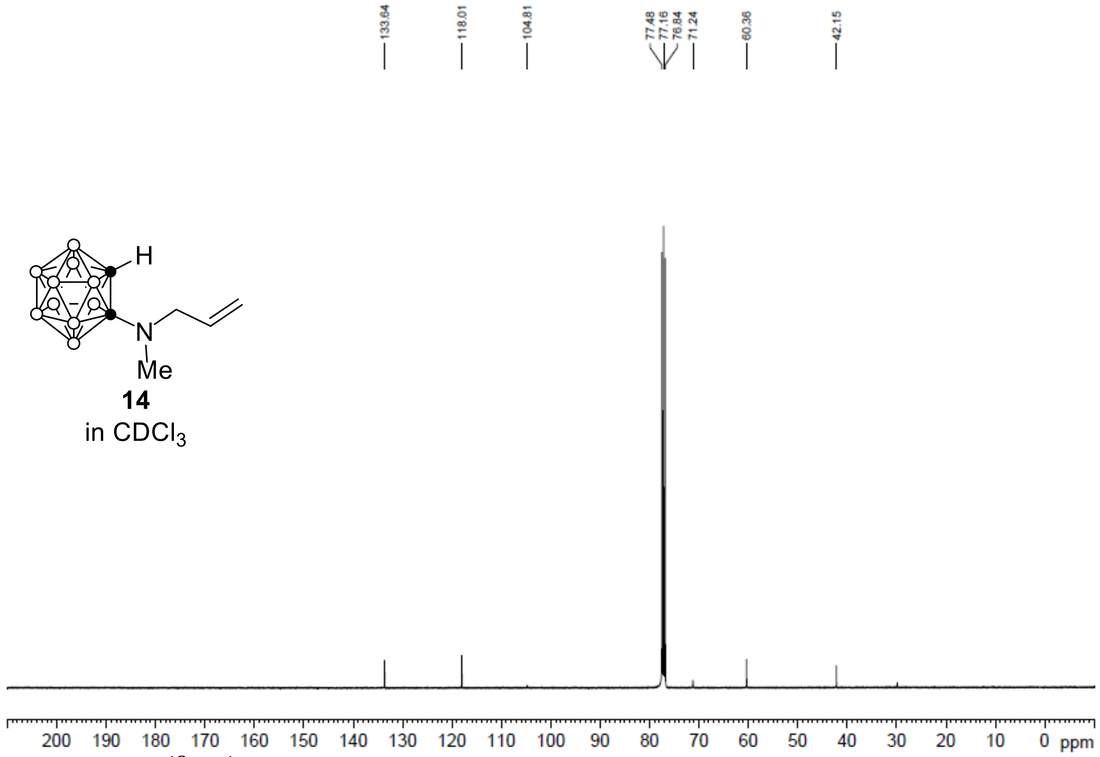

Figure S40. $^{13}C\{^{1}H\}$ NMR spectrum of **14** in $CDCl_3$.

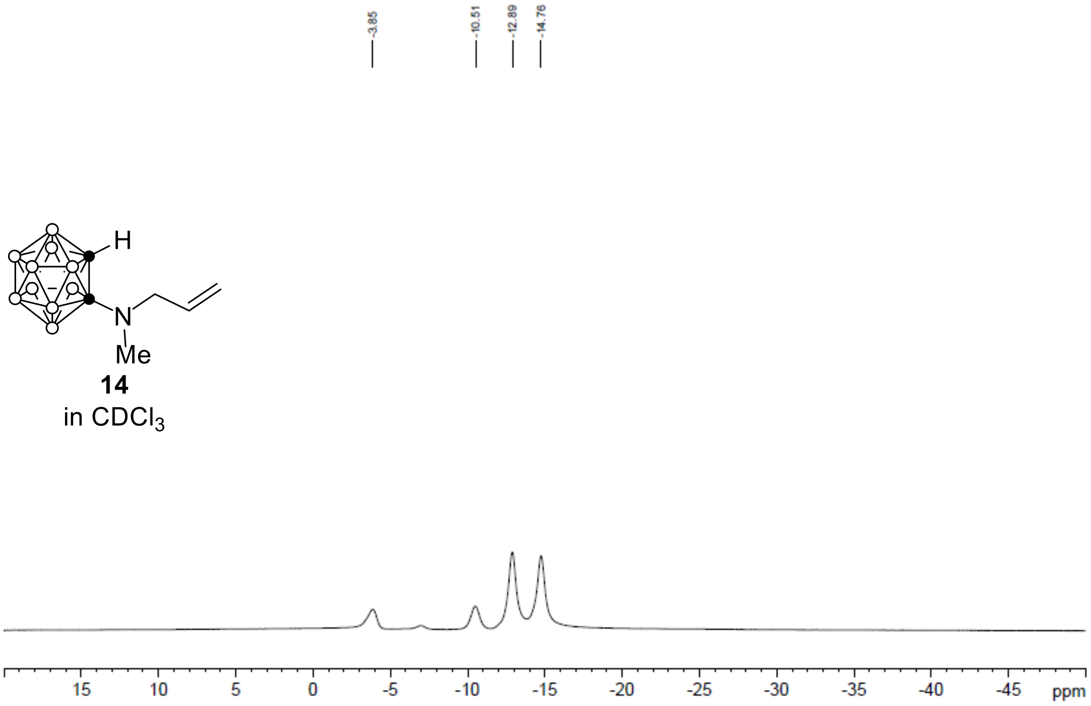

Figure S41. $^{11}B\{^{1}H\}$ NMR spectrum of **14** in $CDCl_3$.

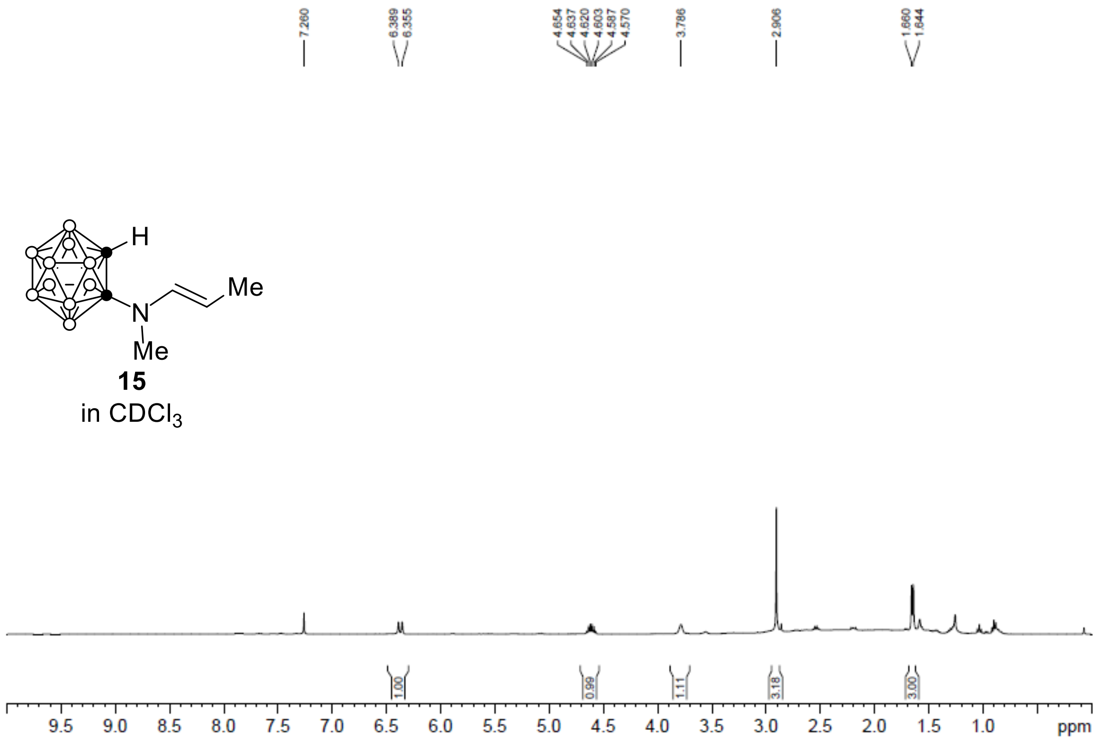

Figure S42. ^{1}H NMR spectrum of **15** in $CDCl_3$.

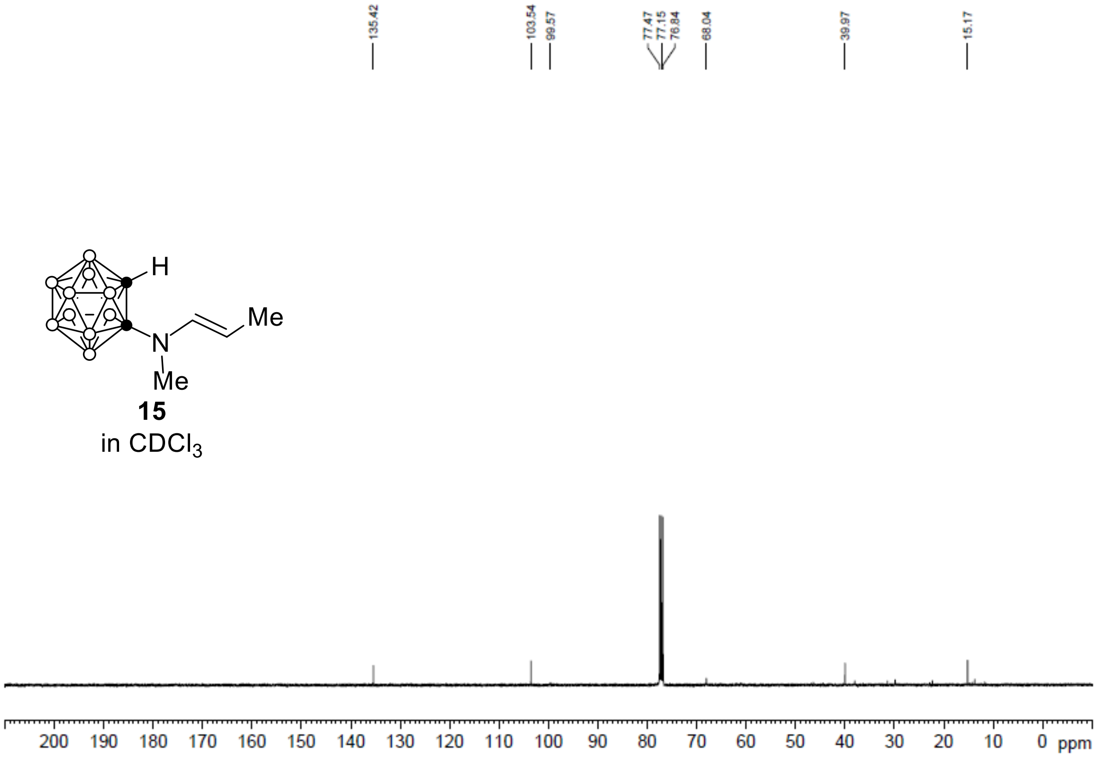

Figure S43. ^{13}C{^{1}H} NMR spectrum of **15** in $CDCl_3$.

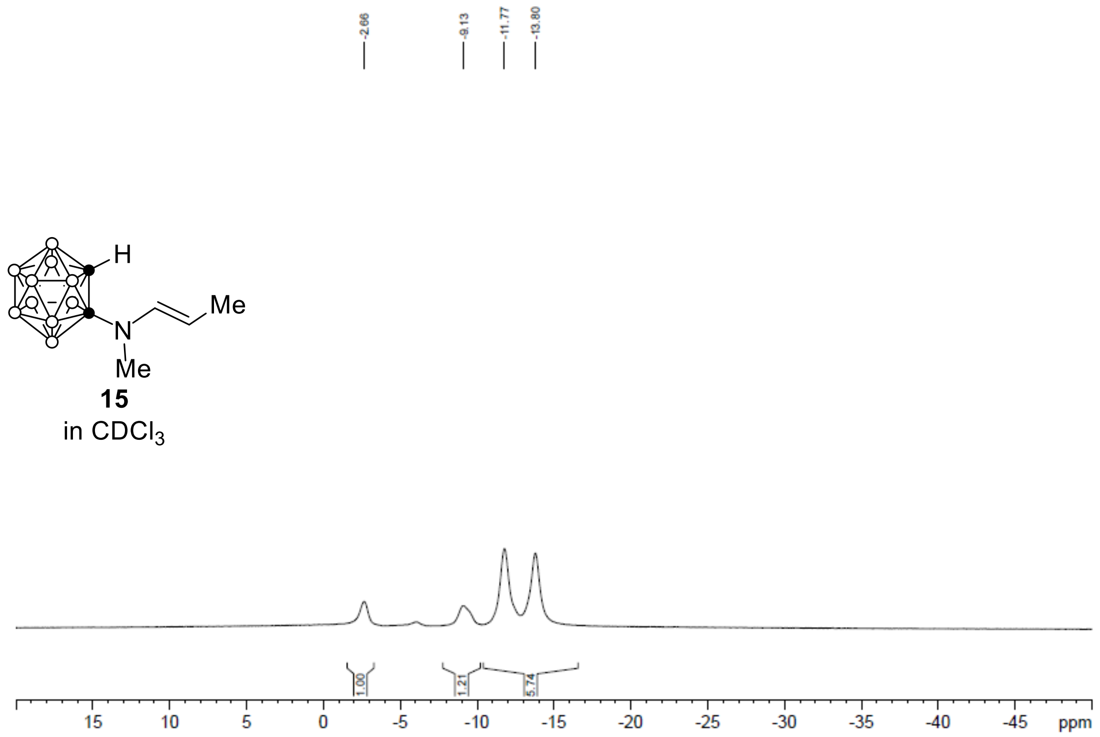

Figure S44. $^{11}B\{^{1}H\}$ NMR spectrum of **15** in $CDCl_3$.

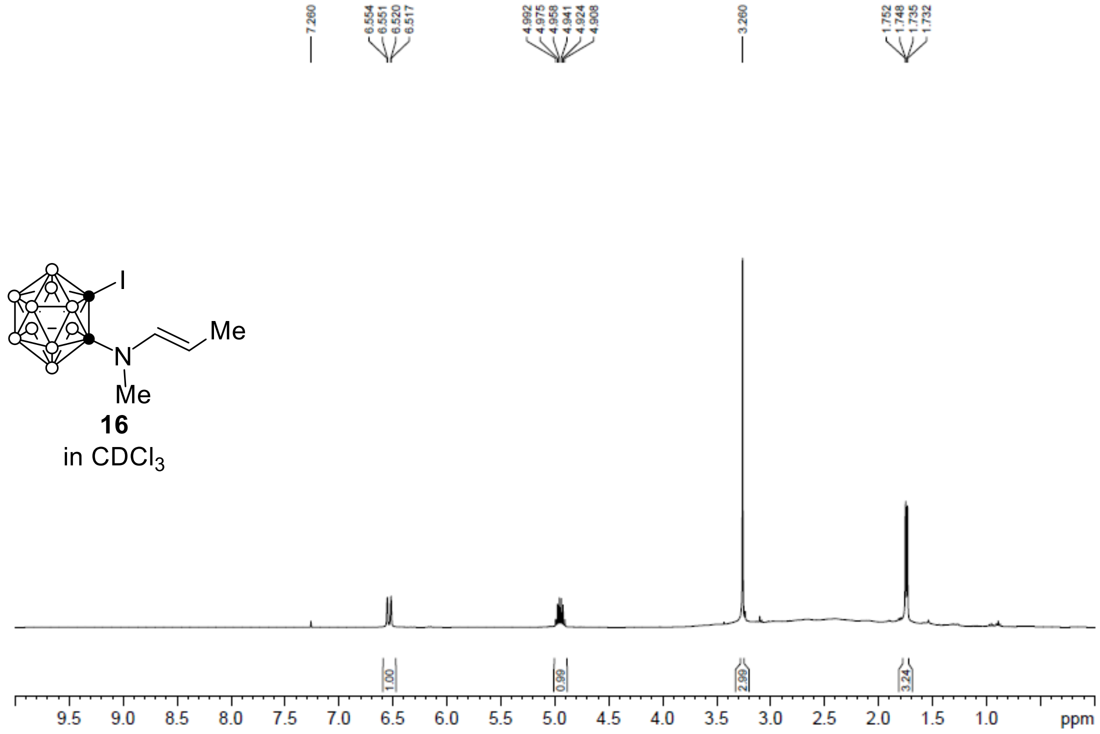

Figure S45. ^{1}H NMR spectrum of **16** in $CDCl_3$.

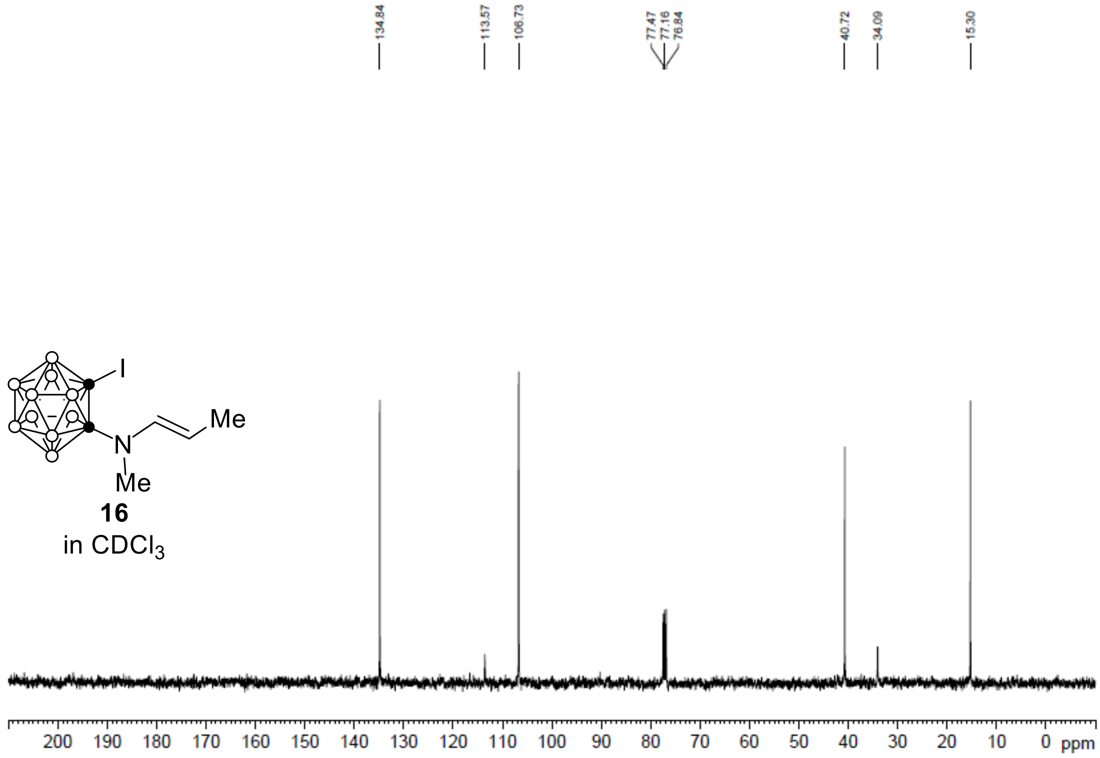

Figure S46. $^{13}C\{^{1}H\}$ NMR spectrum of **16** in $CDCl_3$.

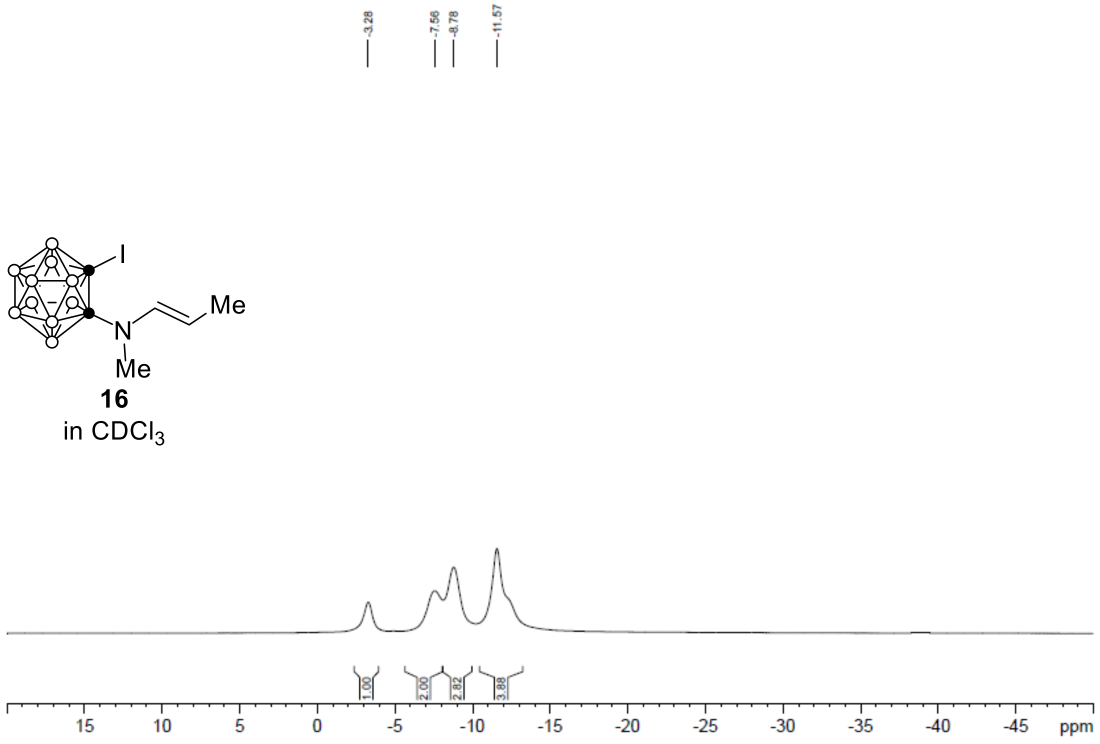

Figure S47. $^{11}B\{^{1}H\}$ NMR spectrum of **16** in $CDCl_3$.

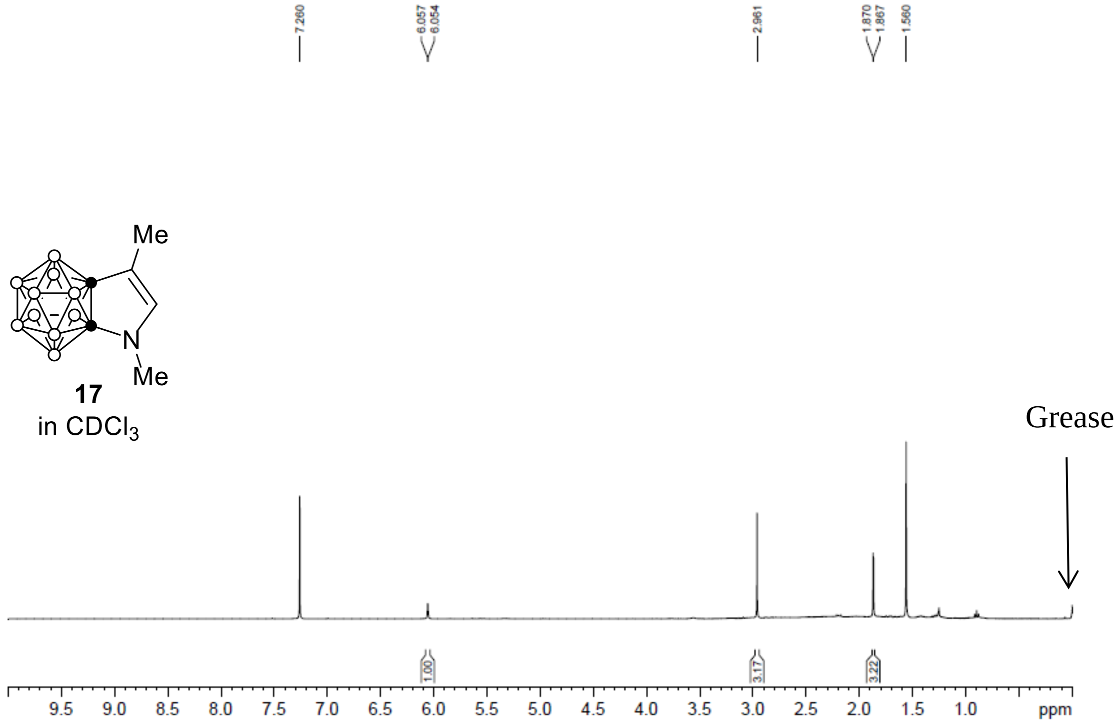

Figure S48. ^{1}H NMR spectrum of **17** in $CDCl_3$.

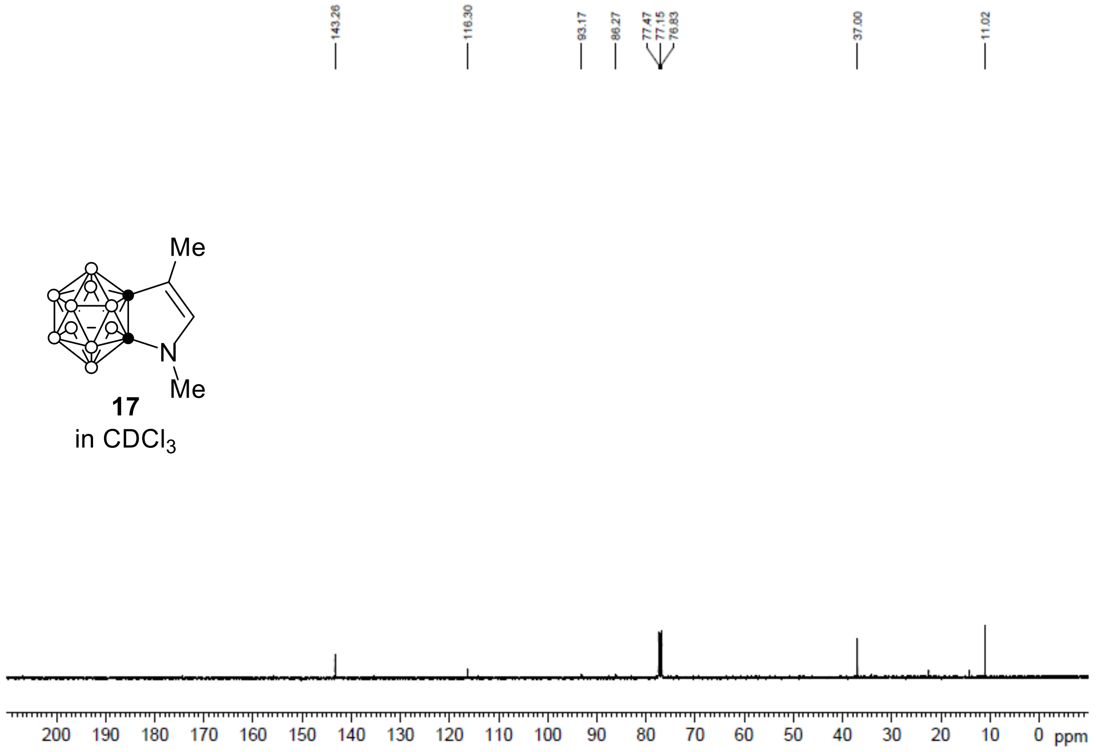

Figure S49. $^{13}C\{^{1}H\}$ NMR spectrum of **17** in $CDCl_3$.

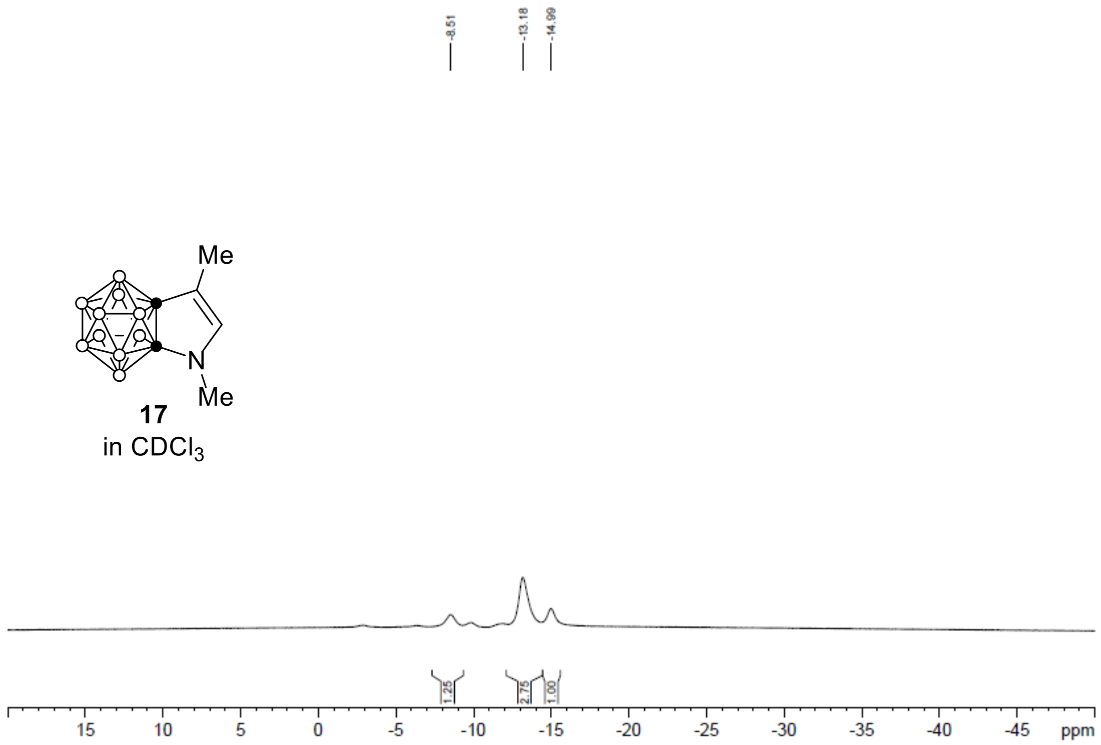

Figure S50. $^{11}B\{^{1}H\}$ NMR spectrum of **17** in $CDCl_3$.

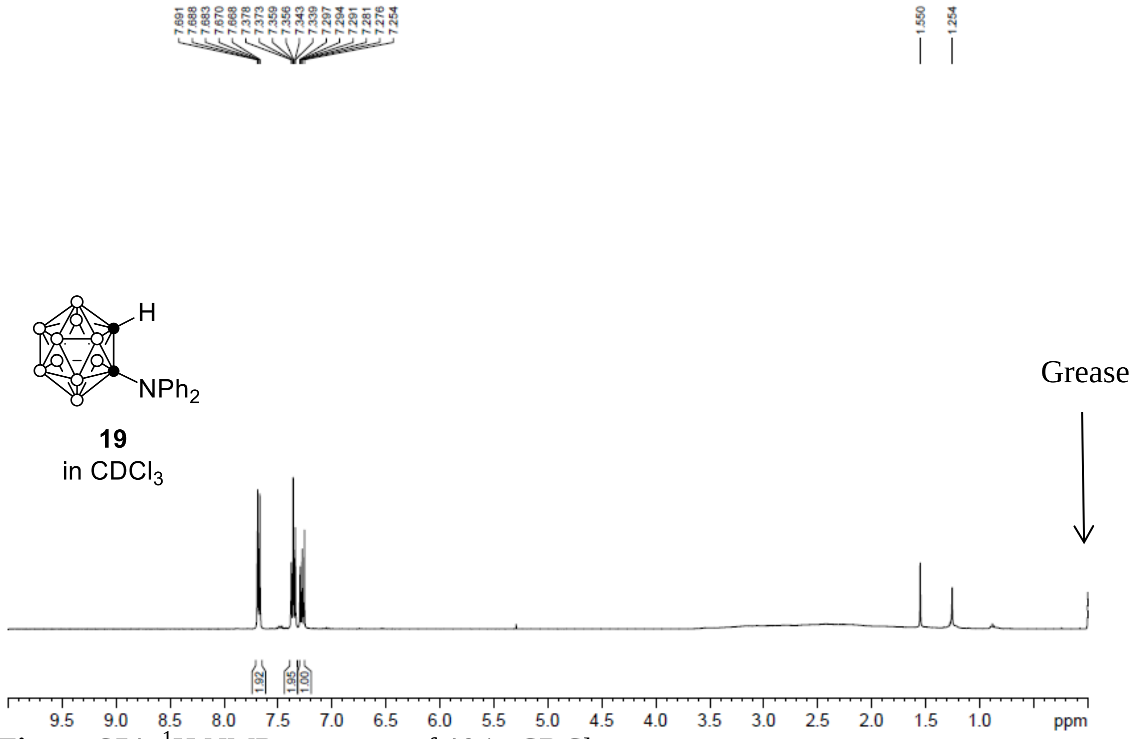

Figure S51. ^{1}H NMR spectrum of **19** in $CDCl_3$.

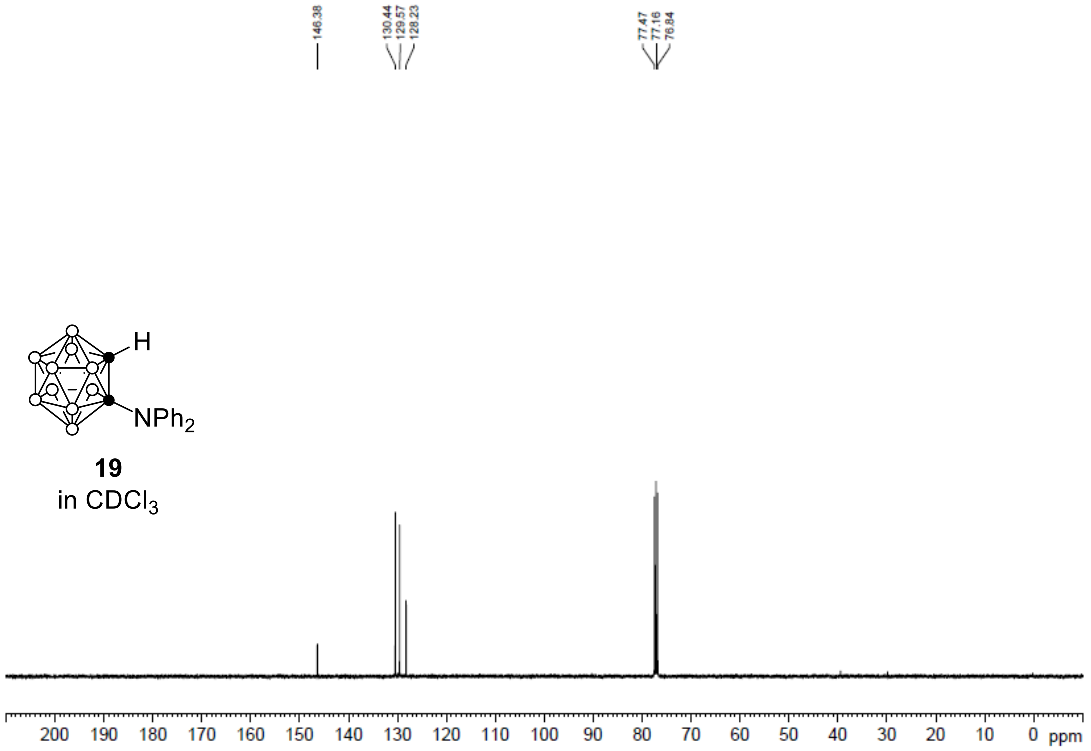

Figure S52. $^{13}C\{^1H\}$ NMR spectrum of **19** in $CDCl_3$.

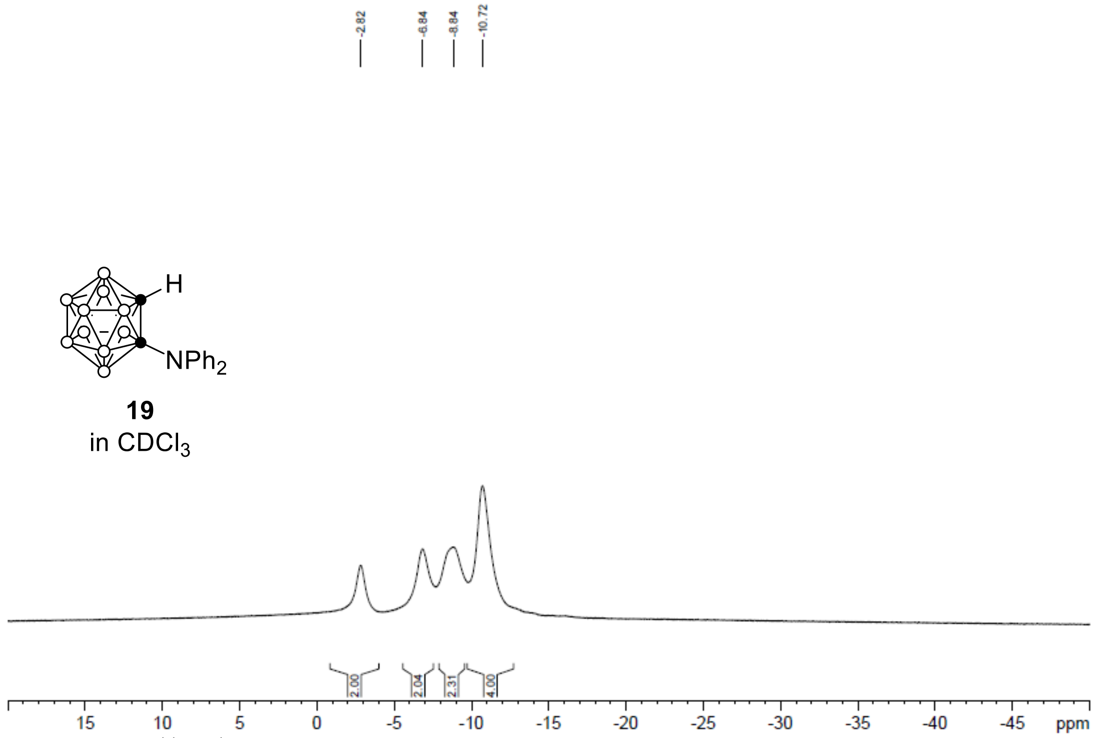

Figure S53. $^{11}B\{^1H\}$ NMR spectrum of **19** in $CDCl_3$.

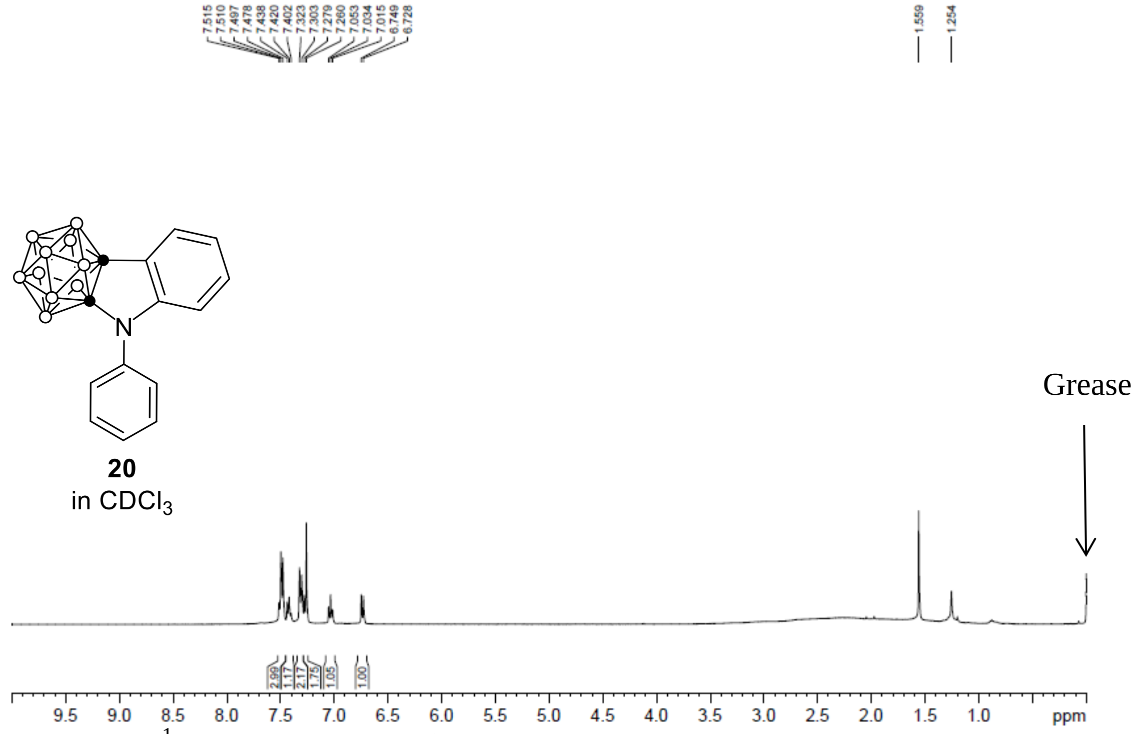

Figure S54. ^{1}H NMR spectrum of **20** in $CDCl_3$.

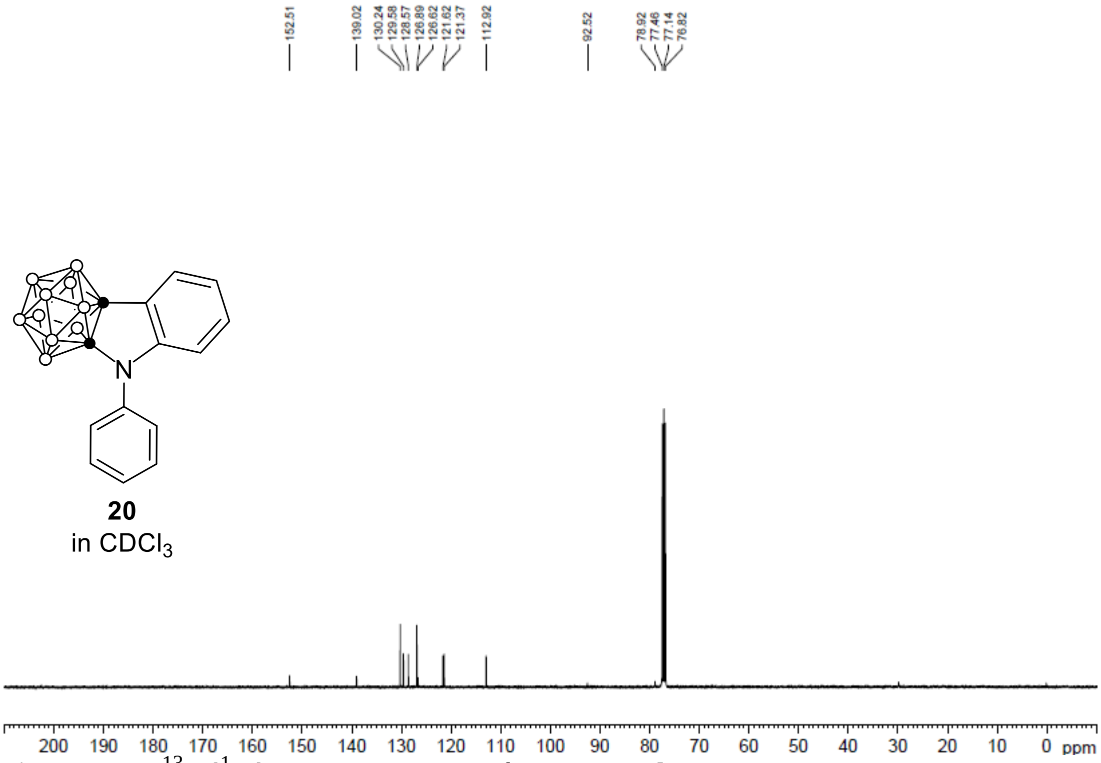

Figure S55. ^{13}C{^{1}H} NMR spectrum of **20** in $CDCl_3$.

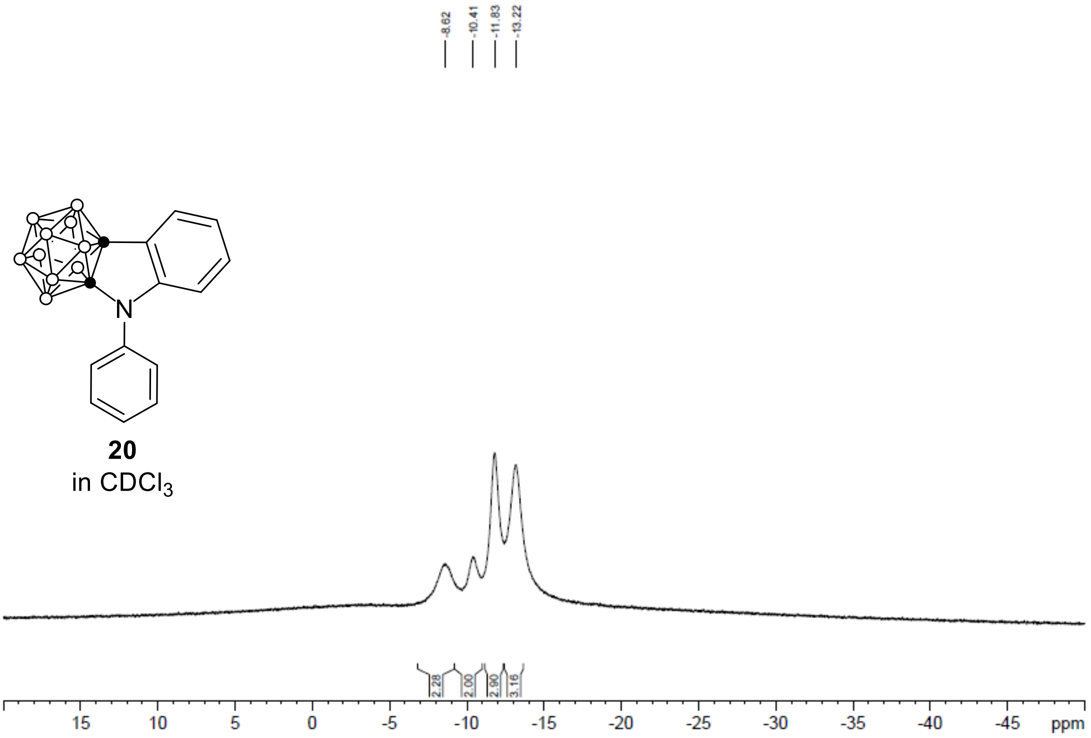

Figure S56. $^{11}B\{^1H\}$ NMR spectrum of **20** in $CDCl_3$.
